# Elucidating the Mechanisms of Ion Permeation through Sub-Nanometer Graphene Pores: Uncovering Free Energy Barriers via High-Throughput Molecular Simulations

**DOI:** 10.1021/acsnano.5c13306

**Published:** 2025-10-17

**Authors:** Andres F. Ordorica, Peifu Cheng, Pavan Chaturvedi, Peter T. Cummings, Piran R. Kidambi

**Affiliations:** † Department of Chemical and Biomolecular Engineering, 5718 Vanderbilt University, Nashville, Tennessee 37212, United States; ‡ School of Engineering & Physical Sciences, 3120 Heriot-Watt University, Edinburgh EH14 4AS, U.K.; § Department of Mechanical and Aerospace Engineering, 3463 University of Florida, Gainesville, Florida 32611, United States

**Keywords:** graphene, nanopores, molecular dynamics simulation, constant potential method, ion selectivity

## Abstract

Understanding ion transport through subnanometer graphene nanopores is critical for advancing nanoscale filtration technologies and uncovering the molecular mechanisms underlying selective ion permeation. Owing to their atomic thickness and tunable pore sizes, nanoporous graphene membranes serve as a model system for probing ion selectivity and hydration behavior under spatial confinement. This work investigates the transport of Na^+^, Cl^–^, K^+^, and water through graphene nanopores to elucidate their ion-sieving characteristics. Free energy barriers associated with ion and water permeation are quantified, offering insight into the energetic costs of dehydration and translocation through nanopores. Selective ion transport is further examined using the constant potential method (CPM), which more accurately reflects experimental electrochemical conditions, and allows for the selective permeation of K^+^ over Na^+^ within nanoporous graphene membranes. The role of externally applied electric fields is also explored to assess their impact on ion hydration and transport dynamics. Together, these results contribute to a deeper mechanistic understanding of ion confinement, hydration, and selective permeation in nanoporous atomically thin membranes.

## Graphical abstract

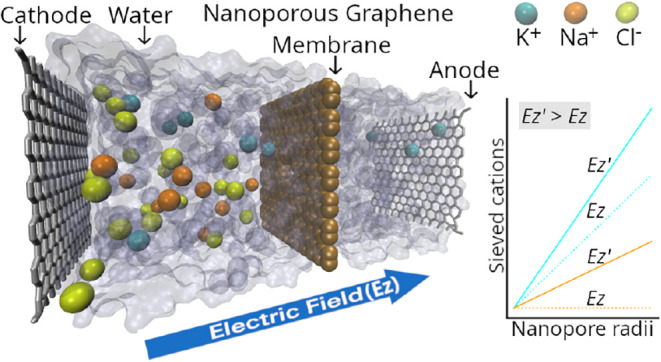

Understanding transport at the nanoscale and subnanometer scales is central to a range of membrane-based separation processes, where controlling molecular and ionic motion through confined environments is fundamental to achieving selective and efficient separations. One example of such processes is desalination, a technology essential for addressing global water scarcity that has spurred efforts to improve efficiency,^1,2^ particularly for seawater treatment in coastal regions.^3^ Most current desalination plants employ reverse osmosis (RO) and nanofiltration using polymeric membranes.^4^ However, limitations such as high energy consumption and low water flux have motivated efforts to develop advanced membrane materials with enhanced performance.^5–7^ Thin two-dimensional (2D) membranes have emerged as promising alternatives due to their atomic-scale thickness and ability to form subnanometer pores that enhance water permeability and salt rejection,^8–10^ with nanoporous materials offering additional advantages through mechanisms such as size exclusion,^11–13^ charge repulsion,^14–16^ chemically specific interactions,^17,18^ and ion dehydration.^19–24^ Graphene, a 3.4 Å-thick, carbon-based ultrathin membrane, has been extensively studied as an alternative to polymeric membranes,^25^ and has demonstrated superior performance over zeolites and thin-film composite (TFC) membranes,^20^ offering 2–3 orders of magnitude higher permeability due to its nanoporous structure and atomic-scale thickness.^26^ Nanopores in such two-dimensional materials have gained considerable attention not only for their potential in separation technologies but also as simplified, robust analogues of biological ion channels,^27^ where their minimal resistance to ion translocation enables detailed investigation of molecular-scale transport mechanisms.^14,19^ Recent advancements in nanoporous graphene membrane fabrication—such as the creation of subnanometer pores via size-selective interfacial polymerization (IP) following high-density nanopore formation,^28^ oxygen plasma etching,^29^ and ion bombardment combined with chemical oxidative etching^30^ are essential for achieving efficient molecular sieving.^5^ Subnanometer pores enable selective ion permeation by potentially allowing dehydration,^24^ as hydrated ions encounter substantial energy barriers dependent on their hydration radius and interactions with surrounding water.^31,32^ Atomically precise pores could function as molecular sieves, discriminating between molecules with minute size and shape differences.^15,33^ Ion-selectivity and transport studies have demonstrated partial ion dehydration through graphene’s subnanometer pores (see Figure 1), providing compelling experimental evidence of dehydration-driven selectivity,^23^ while free-energy barriers arising from dehydration effects and steric hindrance at graphene nanopore edges significantly influence ion transport.^11,34^ Molecular dynamics (MD) simulations demonstrate that steric hindrance and dehydration effects govern ion exclusion in sub-0.55 nm graphene nanopores,^18,23,26^ with energy barriers for K^+^, Na^+^, and Cl^–^ rising sharply as pore size decreases, while water faces minimal resistance.^35^ While the choice of water model may appear to have limited impact on the translocation of water itself,^36^ it significantly influences the energetic barrier associated with ion transport, as the model’s parameters affect ion hydration behavior and, consequently, permeation through the nanopore.^15,21,23,35^ Reported ion permeation barriers range from ∼20 to 25 kJ/mol in pores with a radius of 0.45 nm,^15^ and external electric fields further modulate transport by enhancing ion mobility,^37^ breaking ion clusters,^38,39^ and enabling ultrafast, selective translocation akin to biological channels.^14,27,40,41^ MD simulations confirm that fields in the 0.01–0.4 V/Å range increase water flux,^42^ favor K^+^ over other ions,^43^ and improve both permeate flow and salt rejection.^23,44,45^ While electric fields applied perpendicular to the membrane can enhance anion rejection by opposing their transport, they may simultaneously reduce cation rejection due to electrostatic forces that promote cation migration through functionalized nanopores.^27^ Ion-sieving under electric fields is primarily driven by hydration shell disruption,^46^ with weak fields having minimal impact, and strong fields inducing significant structural changes,^40,46^ including electrofreezing.^47^ Water dissociation occurs near 3.5 V/nm,^48^ a novel ice phase forms around 2.3 V/nm,^40^ and coordination structure changes emerge at ∼1 V/nm.^49^ Therefore, fields below 1 V/nm are recommended to preserve hydration structure in subnanometer pores.

**Figure 1:**
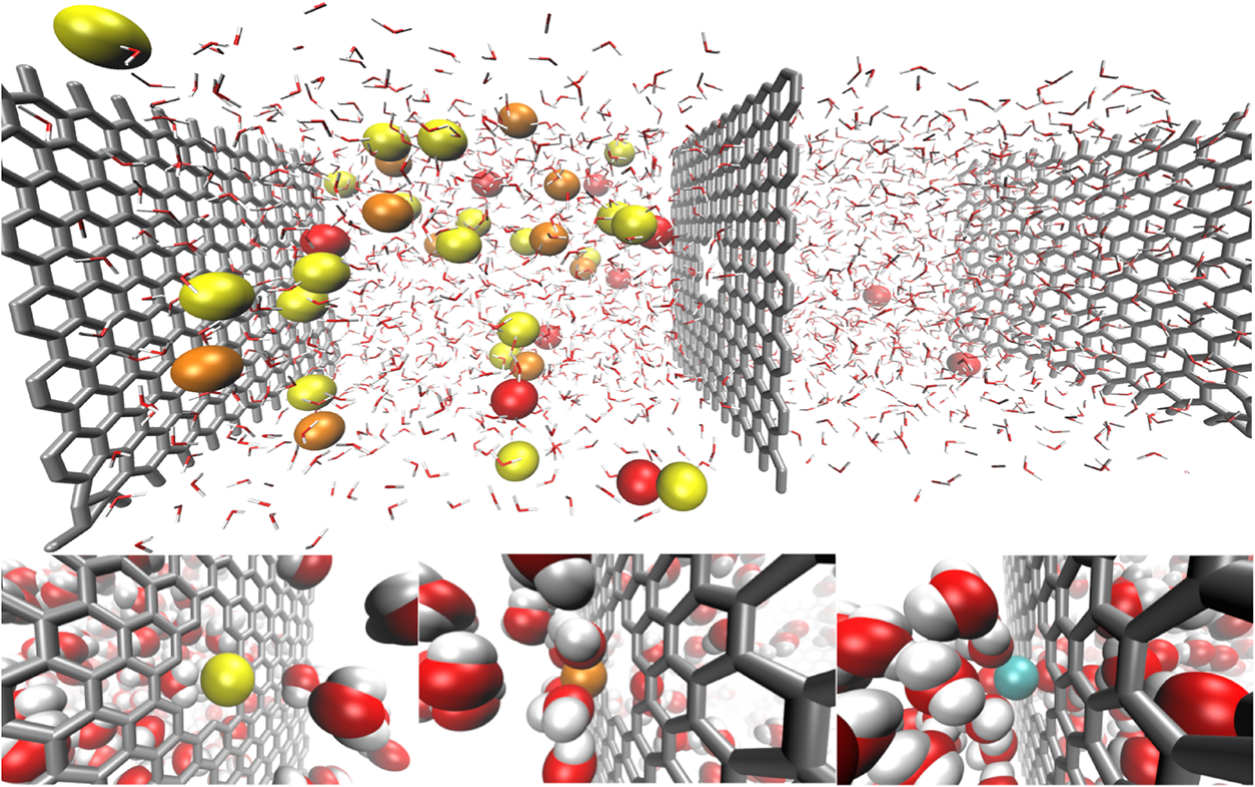
Top panel: Snapshot of the conclusion of a CPM simulation. Bottom panel: Translocation of various ions through the graphene nanopore.

The most common approach to account for the electric field in such systems is the constant field method (CFM),^23,38,42–44,50^ which adds a Coulomb-type force to every atom in the simulation. However, the CFM method has limitations in capturing the electric field modification that occurs in experimental configurations, where the field weakens as it penetrates the liquid sample.^51^ The constant potential method (CPM)^52,52^ introduces two electrodes in direct contact with the system, maintaining fluctuating charges to uphold a constant potential and capturing variations in coordination number and local electric field. In contrast, the CFM method imposes a uniform electric field, whereas CPM generates a self-consistent, nonuniform electric field within the simulation cell.^53,54^ This makes CPM especially suitable for comparing MD simulations to continuum models^55^ and for studying electrochemical effects.^53,56^ Simulations of a NaCl aqueous solution by Bonakala and Hasan^51^ showed that the CPM method yields a broad distribution of water OH–field angles (0–104°, with a preferred orientation near 30°), unlike the rigid 0 or 180° alignments seen in CFM, reflecting the presence of spatially varying electric fields; when taking into account that strong fields can disrupt hydration shells through dipole reorientation and enhanced hydrogen bonding,^46^ and that ion translocation through nanopores depends on the shedding of water molecules from the solvation shell,^19–24^ such a field description is essential for accurately capturing the molecular mechanisms of electric field-driven ion translocation across nanopores.

In this study, we investigate the transport of Na^+^, Cl^–^, K^+^ and water through subnanometer graphene nanopores, with a focus on the molecular-level mechanisms governing ion selectivity. A multistep approach is employed: free energy barriers are first quantified using umbrella sampling (US), revealing the dehydration penalties for ion and water permeation. These results are then integrated into CPM simulations to examine ion transport under applied electric fields and evaluate the preferential permeation of K^+^ over Na^+^. In parallel, simulation outcomes are compared to both diffusion-driven and potential-driven experimental observations conducted through nanopores of the same size, to further contextualize the results. By coupling enhanced sampling with electric field-driven simulations and experimental benchmarks, this work aims to provide a deeper mechanistic understanding and establish a framework for ion dehydration, nanoscale confinement, and selective transport through atomically thin nanoporous membranes.

## Results and Discussion

### Experimental Characterization through Water and Ion Transport across the Membrane

Graphene (Gr) was synthesized at 900 °C and transferred onto a polycarbonate track-etched (PCTE) support, then treated with ultraviolet light (UV)/ozone and followed by size-selective interfacial polymerization (IP) using octa-ammonium polyhedral oligomeric silsesquioxane (POSS) and trimesoyl chloride (TMC) (see Membrane Fabrication section for synthesis details). As shown in Figure 2B, this process preserves small nanopores (<0.5 nm), seals intermediate ones (∼0.5–1.8 nm), and closes larger pores or tears (>1.8 nm) with POSS, enabling the resulting membrane (see Figure 2A) to exhibit high water permeance and effective salt rejection.^28^

**Figure 2:**
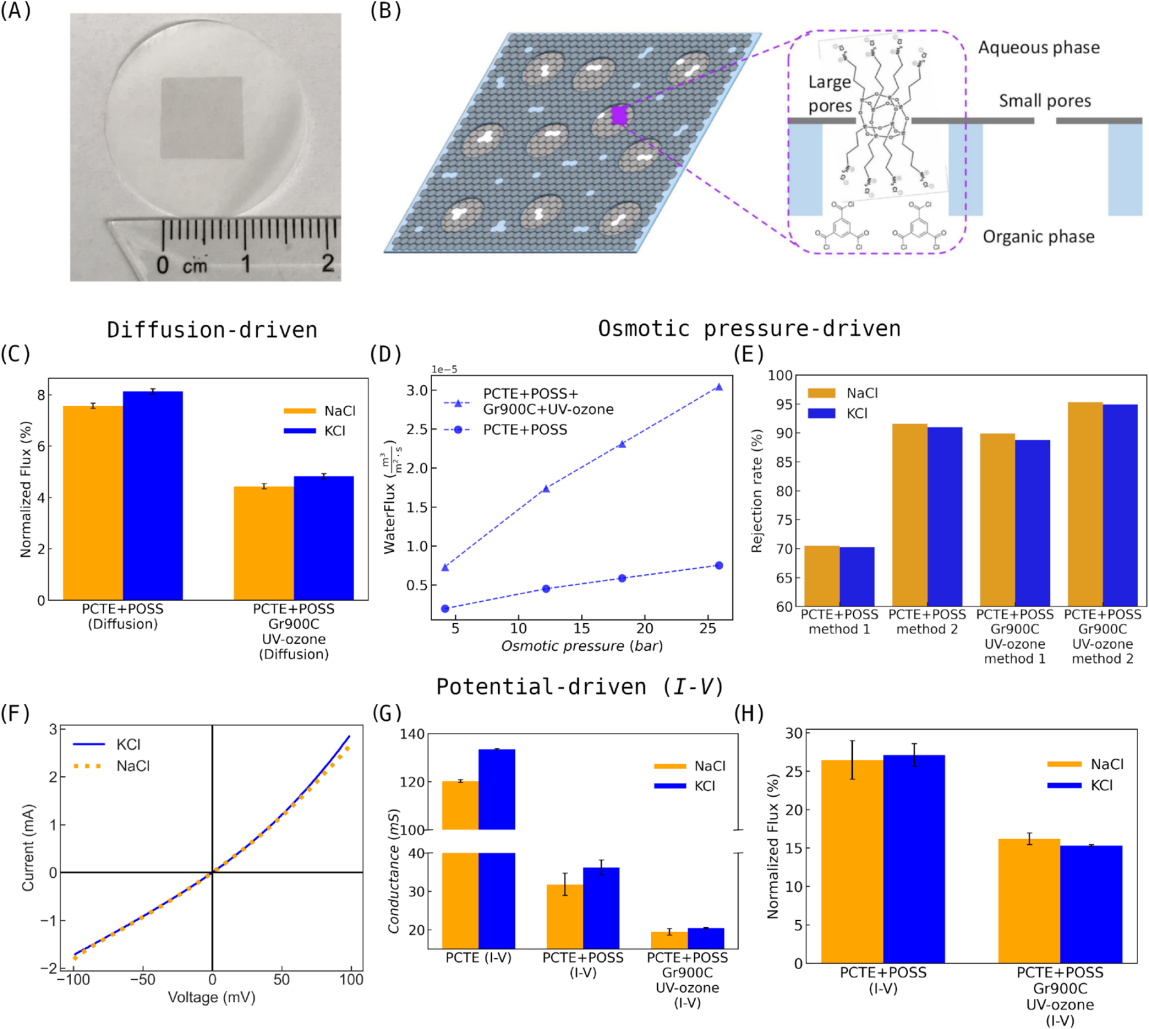
(A) Optical image of graphene transferred onto PCTE support. The dark region represents graphene. (B) Illustration depicting the selective sealing of nanopores in graphene lattice. (C) Normalized flux of diffusion-driven transport through the nanoporous graphene membrane (Gr_900°C_ + UV/ozone + POSS). (D) Water transport as a function of applied osmotic pressure through the nanoporous graphene membrane (Gr_900°C_ + UV/ozone + POSS), with PCTE + POSS as a reference. (E) Salt rejections calculated using two methods for the nanoporous graphene membrane (Gr_900°C_ + UV/ozone + POSS), with PCTE + POSS as a reference. (F) Representative current–voltage (*I–V*) plots for the Gr_900°C_ + UV/ozone + POSS membrane. (G) Conductance (extracted from the linear portion of *I–V* plots) plots for all the membranes. Measurements are performed in 0.1 M KCl and 0.1 M NaCl electrolytes. (H) Normalized flux of potential-driven transport through the nanoporous graphene membrane (Gr_900°C_ + UV/ozone + POSS), with PCTE + POSS as a reference.

The resulting nanoporous graphene membrane (PCTE + Gr_900°C_ + UV/ozone + POSS) was tested by measuring the rejection of two analytes, NaCl and KCl. The solute rejection was computed using two approaches, referred to as method 1 and method 2 (for additional details on the measurements and procedures, see Solute Rejection Measurements section). The NaCl and KCl rejection rates are shown in Figure 2C. Water permeance was calculated by dividing the representative water flux by the corresponding osmotic pressure (see Water Transport Measurements section for experimental details), and the resulting water transport values are shown in Figure 2D. The fast water transport (see Figure 2D) and high salt rejection (see Figure 2C) suggest that a majority of the pores in the graphene membrane are subnanometer in size. Solvated ions face significant transport resistance, particularly when considering the van der Waals diameter of water (0.28 nm) and the hydrated diameters of K^+^ (0.662 nm), Cl^–^ (0.664 nm), and Na^+^ (0.716 nm).^28^

Salt transport experiments measuring via diffusion-driven (Diffusion) and potential-driven (*I*–*V*) experiments revealed a difference, as shown in in the left panel of Figure 3 (see Ionic Conductance Measurements section for experimental details).

**Figure 3:**
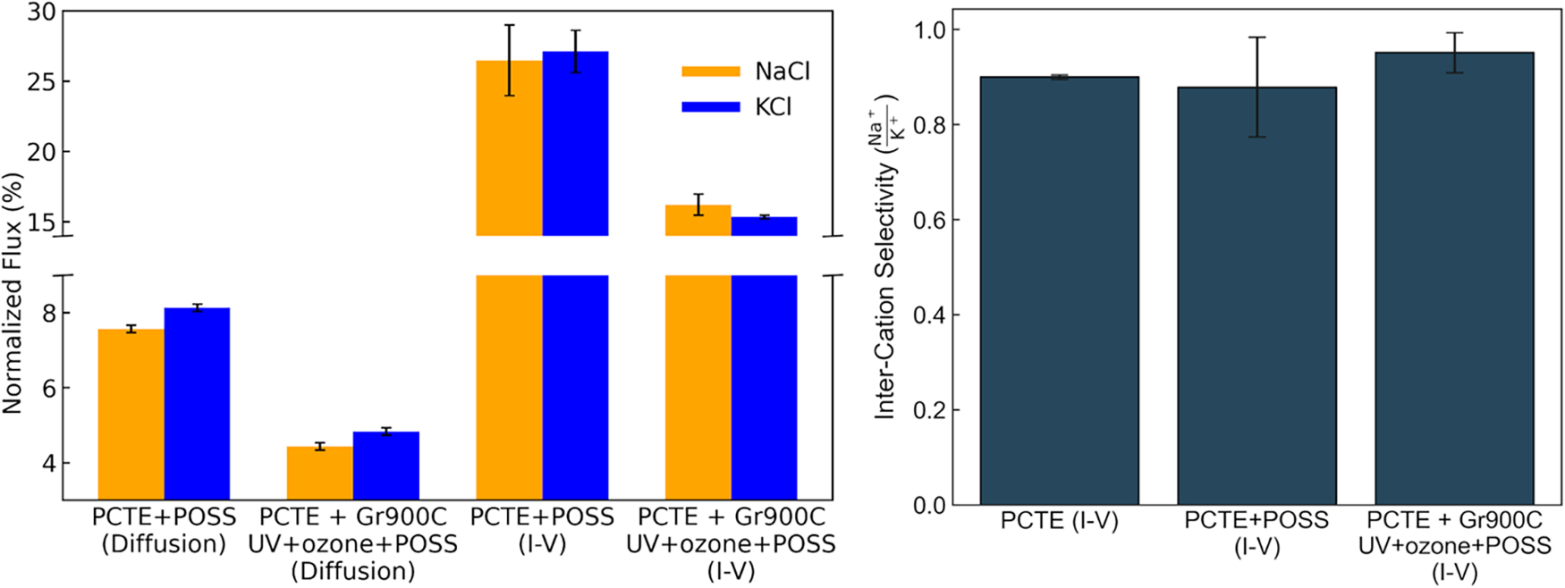
Normalized flux (%, left) values for the diffusion (Diffusion) and potential-driven (*I*–*V*) salt transport experiments of NaCl (orange) and KCl (blue) and intercation selectivity (right) values for the potential driven (*I*–*V*) salt transport experiments of NaCl and KCl.

The elevated normalized flux (PCTE + Gr_900°C_ + UV/ozone + POSS/PCTE + POSS) underscores a distinction between diffusion-driven and potential-driven transport mechanisms through the nanoporous graphene membrane.

As shown in previous cation separation studies using membranes,^57^ an applied potential difference generates an electric field that drives ion separation, consistent with the observed increase in ion permeation under an external electric field.^24^ Electric field-driven ion transport across graphene nanopores is largely governed by differences in electrophoretic mobility and hydration energy,^24,58^ which together determine each ion’s ability to shed water molecules from its solvation shell and traverse subnanometer pores.^59^ K^+^, which has lower hydration energy and higher electrophoretic mobility than Na^+^,^24,58^ is expected to exhibit comparable normalized flux to Na^+^ when transported through nanopores or defects larger than the hydrated radii of both ions, resulting in an intercation selectivity (ICS, see Ionic Conductance Measurements section) close to 1. In the same study,^60^ the ICS of Na^+^ over K^+^ was found to increase (>1.0) with increasing pore size, suggesting size-dependent preferential transport. On the other hand, Fu et al.^24^ conducted electric field-driven permeation studies across graphene nanopores with an average radius of 0.4 nm and reported an ICS of Na^+^ over K^+^ of 0.9, indicating a weak but measurable ability of the membrane to distinguish between the two ions, with slight preferential transport of K^+^. As shown in Figure 3, the normalized flux (left panel) reveals a small difference of less than 5%, with Na^+^ slightly exceeding K^+^. This is reflected in the ICS of Na^+^ over K^+^ $\left(\mathrm{ICS}(\frac{{\mathrm{Na}}^{+}}{K^{+}})\approx 0.95\right)$ being close to—but slightly less than—1 (see Figure 3, right panel), suggesting slight preferential transport of K^+^ ions. This is likely due to pore size and shape distributions of the nanopores or defects in the graphene membrane (PCTE + Gr_900°C_ + UV/ozone + POSS) that are incapable of differentiating between the cations.

### Dehydration-Driven Permeation Analysis of Ions Using Umbrella Sampling (US)

Using the potential of mean force (PMF) profiles obtained via umbrella sampling molecular dynamics (US-MD) simulations the magnitude of K^+^/Na^+^ selectivity (*S*) of the graphene nanopores can be quantified by the difference between the PMF profiles. These profiles are shown in Figure 4 for K^+^,Na^+^, Cl^–^, and a single water molecule passing through nanopores of different radii. Across all scenarios, the PMF profiles consistently exhibits a distinct energy maximum just before the ion or molecule enters or exits the nanopore, indicating a free energy barrier that must be overcome during translocation.^17,18,24,43^ This barrier reflects the energetic penalty associated with transitioning through the pore’s varying radii and solvation environment, as shown in Figure 5. As observed in previous water permeation simulations through graphene nanopores,^18^ the PMF often features a central energy minimum around ζ ∼ 0 nm, flanked by two entry/exit barriers. This minimum does not represent a conventional barrier but rather a high-energy region where ions or molecules are unlikely to remain stationary and are instead driven to pass through, facilitated by interactions with the nanopore atoms and the surrounding solvent. In cases where this central energy minimum becomes non-negative, it behaves as a saddle point, serving as a transient or metastable location for the permeating species.^24,43^ The emergence and shape of these energetic features underscore the interplay between solvation shell dynamics, nanopore geometry, and electrostatic interactions that collectively govern translocation behavior. The differences in free energy barriers for K^+^, Na^+^, Cl^–^, and a single water molecule traversing graphene nanopores of varying radii arise primarily from two factors: molecular size and hydration strength. Successful translocation through narrow pores requires ions to partially shed water molecules from their first hydration shell (FHS) to reduce steric hindrance with the nanopore walls, as illustrated in Figure 6. However, ion size alone does not fully explain permeability. Prior simulation studies have identified hydration energy—the energy released upon ion dissolution—as a more reliable predictor of ion transport through membranes.^61^ For instance, selective separation of Na^+^ from K^+^ has been shown to depend critically on differences in their hydrated radii and partial desolvation energies, particularly in small-radius nanopores.^11^ Ions with lower hydration energies tend to permeate nanoporous membranes more readily, regardless of their bare ionic radii.^61,62^ This is because the ease with which an ion loses part of its solvation shell depends on the strength of its electrostatic interactions with surrounding water; ions with stronger hydration exhibit higher energetic penalties for dehydration and thus face greater barriers during nanopore translocation. Among the ions examined in this study, the hydrated radius of Na^+^ is the largest, followed by Cl^−^ , and then K^+^.^24,28^ However, Na^+^ has robust electrostatic interactions with water due to its smaller size compared to K^+^,^28^ resulting in stronger interactions between the cation and its FHS.^63^ Throughout the simulations, as each ion or molecule is drawn closer to the nanopore entrance, the number of water molecules in the FHS gradually decreases until it reaches a minimum, as shown in Figure 7. Once the ion or molecule emerges from the nanopore, the FHS water molecule count begins to increase again until it reaches a value similar to the prenanopore state. The observed variations in the free energy barriers can be interpreted using transition state theory, which relates the reaction rate constant for a given species to the free energy barrier, as shown in eq 1 ^43^

1 $$k\left(A\right)\propto e^{-}\frac{\Delta E\left(A\right)}{k_{B}\times T}$$

where *k*_B_ is the Boltzmann constant, *T* is the simulation temperature, and Δ*E* is the maximum energy peak on the PMF curve before traversing the nanopore. With a constant number of atoms/molecules in each simulation, the ion passage rates through nanopores are proportional to their reaction rate constants. Thus, the nanopore’s selectivity for species A over B can be described by the difference in their respective reaction rates, and the natural logarithm of the selectivity, *S*, can be approximated by the logarithm of the ratio *k*(A)/*k*(B), as shown in eq 2 ^43^

2 $$\ln\left(\mathrm{selectivity}\right)=S=\frac{\Delta E\left(A\right)-\Delta E\left(B\right)}{k_{B}\times T}$$

The natural logarithm of the selectivity, *S*, was calculated for K^+^, Na^+^, Cl^–^ ions, and water, and is presented in Figure 8. The results are consistent with previous umbrella sampling (US) simulations,^19,24,27,43^ showing showing difference in transport of K^+^ ions and Na^+^ ions. This is evidenced by the negative values of *S* for K^+^ over Na^+^, and the positive value for K^+^ over water, indicating a preference for water molecules. The nanopore favors K^+^ ions over Na^+^ ions due to the interplay of size, hydration, and electrostatic interactions. This observation suggests that the nanopore’s affinity for water molecules is balanced with a propensity for K^+^ ions, leading to a scenario where K^+^ ions are selectively favored over Na^+^ ions, despite Na^+^.

**Figure 4:**
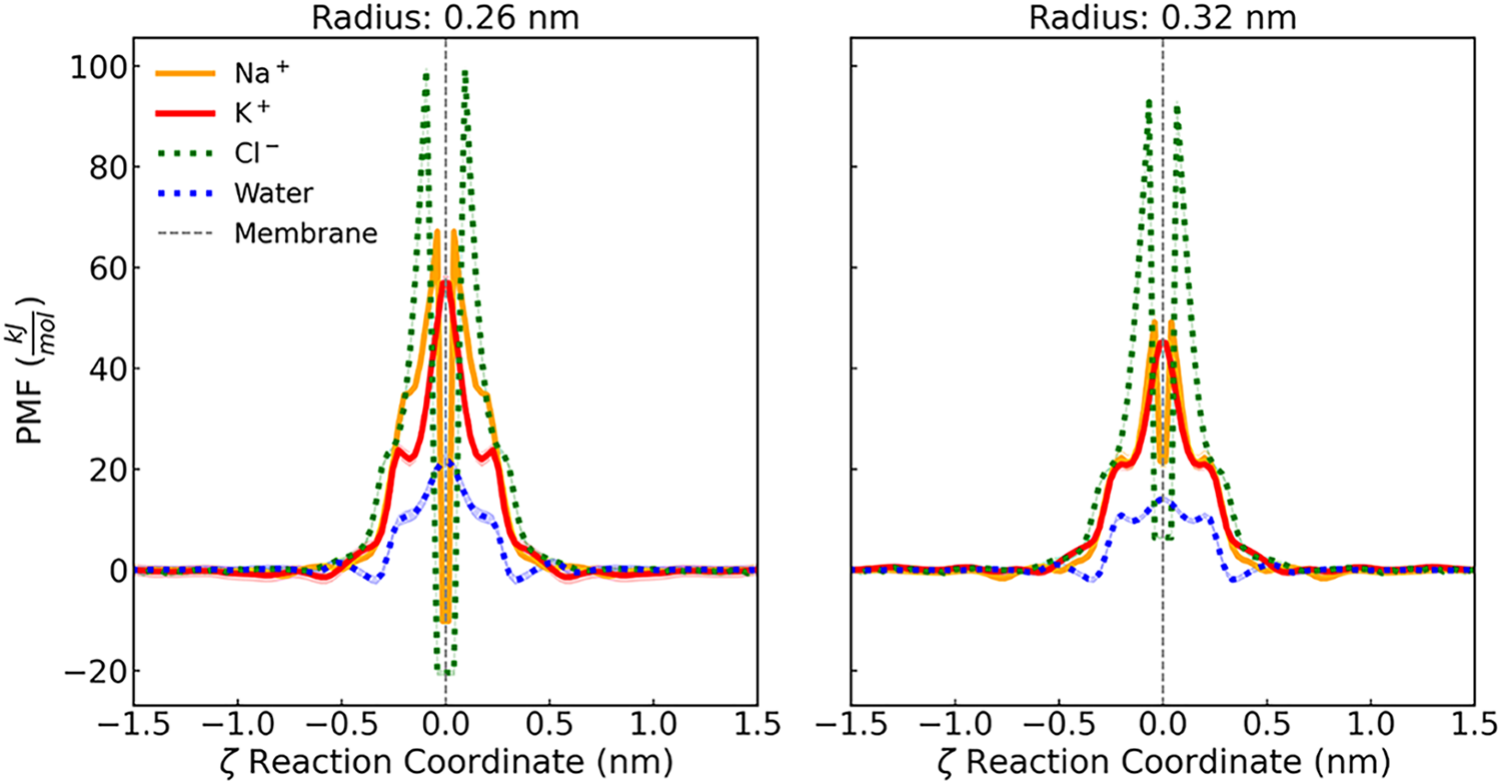
PMFs of K^+^ (red), Na^+^ (orange), Cl^–^ (green), and water molecule (blue) as a function of the reaction coordinate (ζ) for the species gaining passage through a membrane with a graphene nanopore of radii 0.26 and 0.32 nm. For the PMFs of the graphene membrane under all conditions, as well as the comparison of profiles obtained using different water and ion models, refer to the Supporting Information.

**Figure 5:**
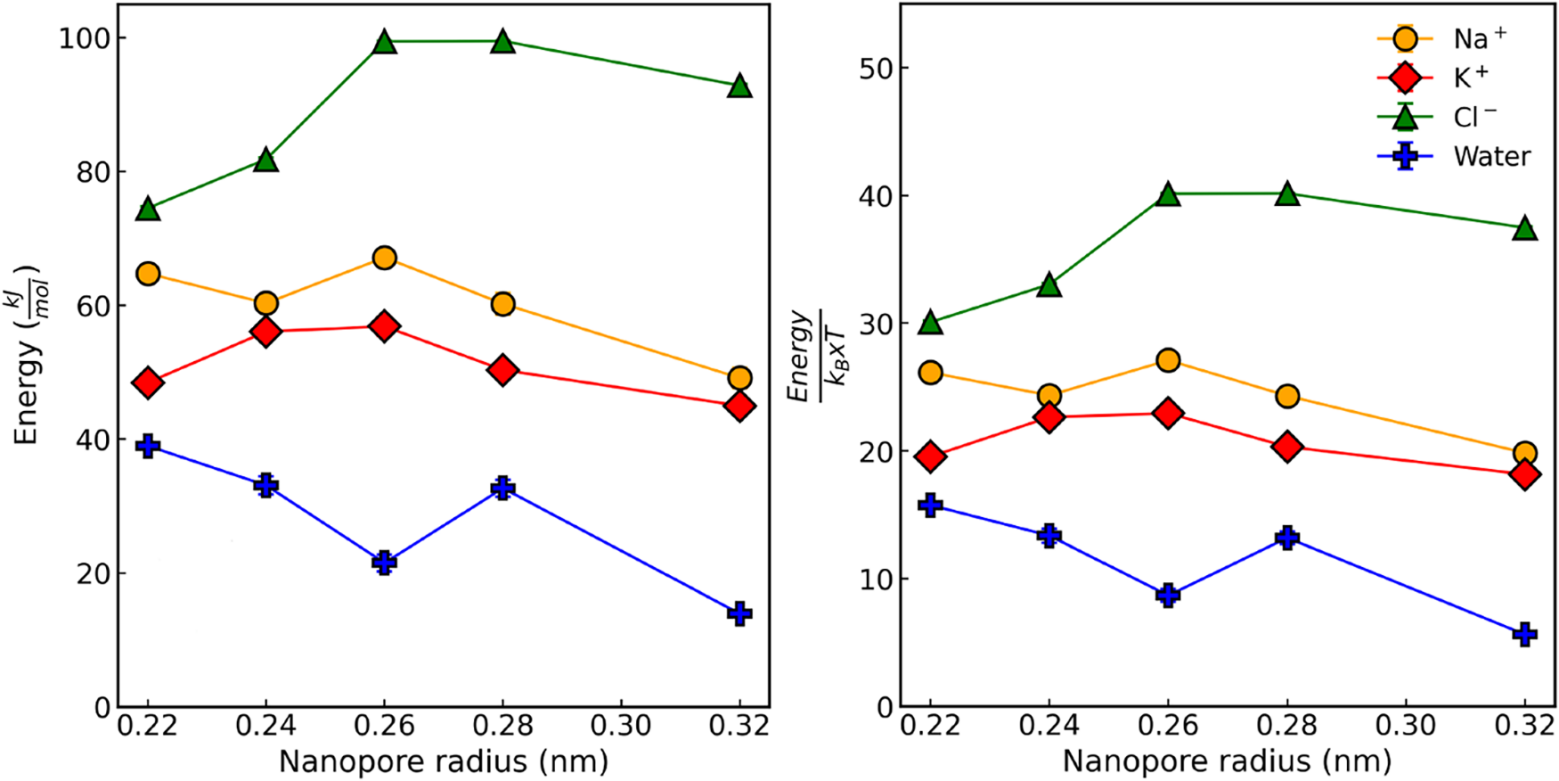
Energy barrier, in kJ/mol, (left) and energy/*k*_B_ × *T* (right), that must be surpassed to achieve passage as a function of the graphene membrane nanopore radii for K^+^ (red), Na^+^ (orange), Cl^–^ (green), and water (blue). For a comparison of the energy barrier results for Na^+^ and K^+^ ions through a membrane with a nanopore radius of 0.26 nm using different water and ion models, refer to the Supporting Information.

**Figure 6:**
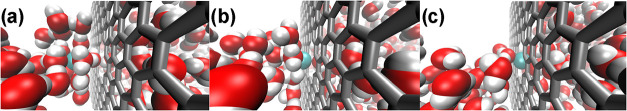
Snapshots capturing the progressive dehydration of the K^+^ ion (cyan) as it translocates through the graphene nanopore, with water oxygen atoms shown in red, hydrogens in white, and graphene carbon atoms in gray.

**Figure 7:**
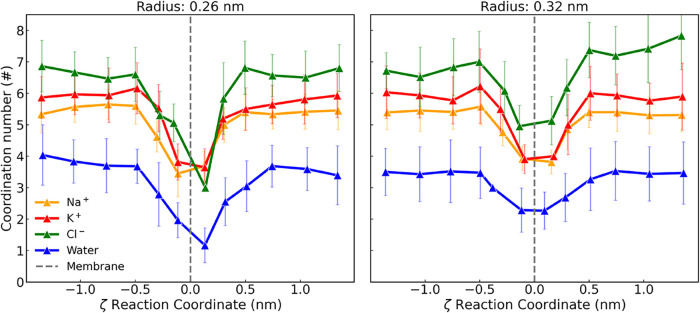
K^+^: O (red) Na^+^:O (orange) Cl^–^:O (green), O:O (blue) coordination numbers in the FHS as a function of the reaction coordinate (ζ) for the species gaining passage through a graphene membrane with a nanopore of radii 0.26 and 0.32 nm. For the coordination number exhibited in a graphene membrane in every case, refer to the Supporting Information.

**Figure 8:**
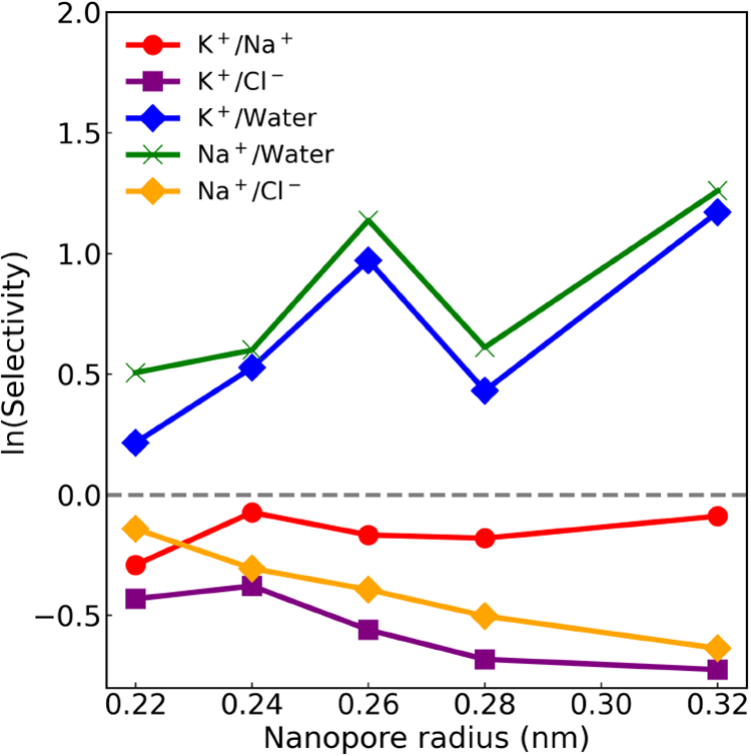
Natural logarithm of the graphene membrane selectivity (*S*) as a function of the nanopore radii for K^+^/Na^+^ (red), K^+^/Cl^–^ (purple), Na^+^/Cl^–^ (orange), K^+^/water (blue), and Na^+^/water (green). For the *S* values without the natural logarithm, refer to the Supporting Information.

### Electric Field Application through the CPM Method

To assess the behavior of the constant potential implementation in the CPM-MD simulations, particularly when simulating the ion solution system near the electrode surface, the calculated 1-dimensional (1D) electrostatic potential profiles were determined using eq 3 ^51^

3 $$\psi \left(z\right)=-\int_{0}^{z}dz^{\prime }\int_{0}^{z\prime }\frac{\rho \left(z^{\prime \prime }\right)}{\epsilon_{0}}dz^{\prime \prime }$$

where ϵ_0_ is the vacuum permittivity and ρ is the charge distribution along the *z*-axis. All the calculated electric potential profiles are shown in Figure 9, depicting the gap between the electrodes, for the full solution of Poisson’s equation in 1D for a slab geometry refer to the Supporting Information. The linear variation of the electric potential in the bulk of the water slab, consistent with the 1D solution of the Laplace equation, confirms that the CPM accurately captures the continuum limit, while the potential exhibits nonlinear behavior at the interface due to surface polarization.^51^ Unlike the uniform electric field (*E*) produced by the CFM, the CPM creates a nonuniform *E*, especially at the electrode and membrane interface. As shown in Figure 9, the electric fields are obtained by computing the derivative of the electrostatic potential along the *z*-direction using the following eq 4.

4 $$E=\frac{\partial \Psi \left(z\right)}{\partial z}$$

The electric field magnitudes generated by the potential difference to drive ion separation in the present study range from 0.0 to 0.63 V/nm. The highest electric field magnitude used in this study (0.63 V/nm) is approximately 10 orders of magnitude smaller than those applied in earlier CFM-MD simulations,^40,64^ yet remains within the range reported in more recent CFM-MD studies of ion translocation across functionalized graphene nanopores.^27^ At the same time, the electric field magnitudes studied here are several orders of magnitude higher than those used in the experiments conducted for this study (electric field generated by a 0.1 V potential difference), as well as in other ion separation experiments using an induced electric field created by a potential difference.^57^ This approach is similar to the use of externally applied pressures that are 10 orders of magnitude larger than those in experiments, aimed at enhancing the signal-to-noise ratio and obtaining well-converged statistics for the time scales involved in MD calculations, particularly in the study of pressure-driven desalination across nanoporous membranes.^20,65^ The electric fields are consistent with the expected values, except in regions near the electrodes and close to the membrane, as shown in Figure 9. This behavior arises due to the presence of the permeate reservoir between the cathode and the membrane. Consequently, Cl^–^ anions tend to migrate closer to the cathode due to electrostatic attraction. Meanwhile, the graphene membrane atoms were modeled with a charge of 0 (*q*_c_ = 0), ensuring neutrality and avoiding charge-related interactions between the sieved species. The slight perturbations observed in both potential and electric field primarily occur in the context of cations (K^+^ or Na^+^) that successfully cross the nanopore under the applied electric field, as illustrated in Figure 10.

**Figure 9:**
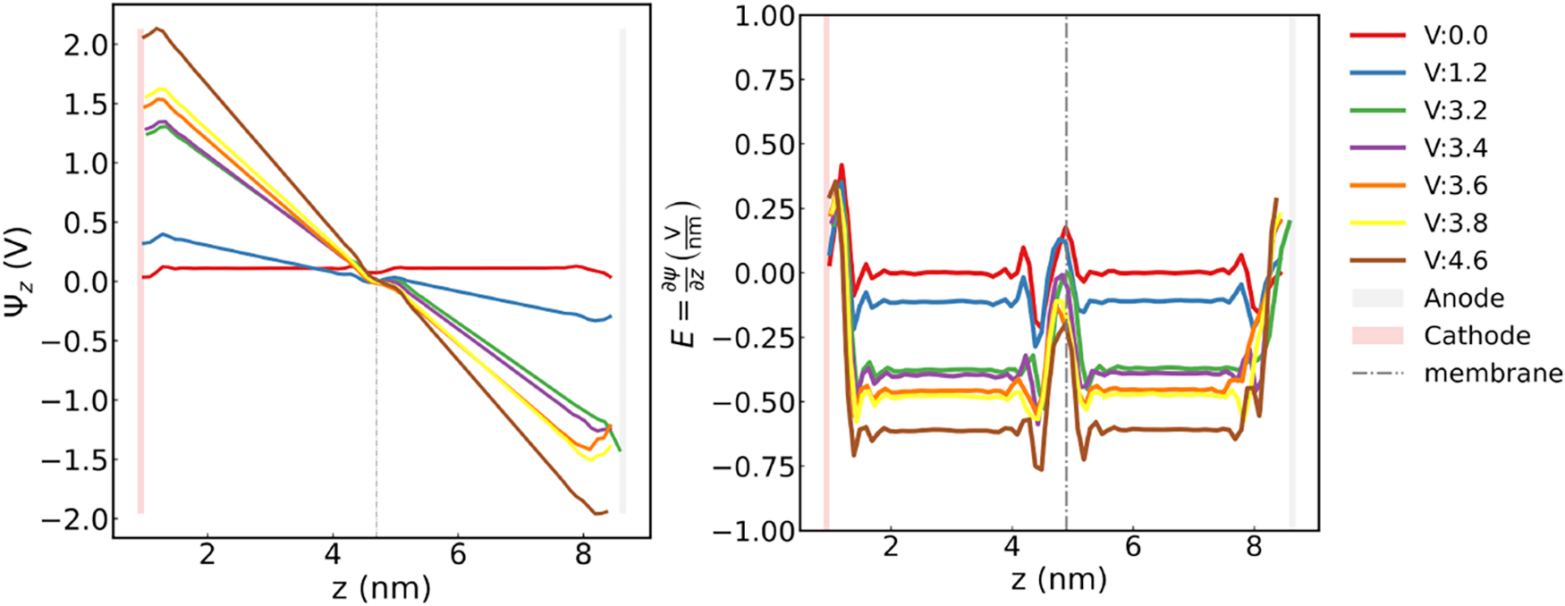
Average electric potential (left) and 1D electric field (*E*, right) profiles along the *z*-direction for the application of 0, 1.2, 3.2, 3.4, 3.6, 3.8, and 4.6 Ψ and potential differences on the graphene electrodes for a graphene membrane with a nanopore radii of 0.26, 0.28, and 0.32 nm. The gray shaded region represents the anode, the red shaded region the cathode, and the dotted vertical line the position of the graphene membrane. For the individual electric potential and electric field profiles in each case and a comparison of these profiles using a different water model, refer to the Supporting Information.

**Figure 10:**
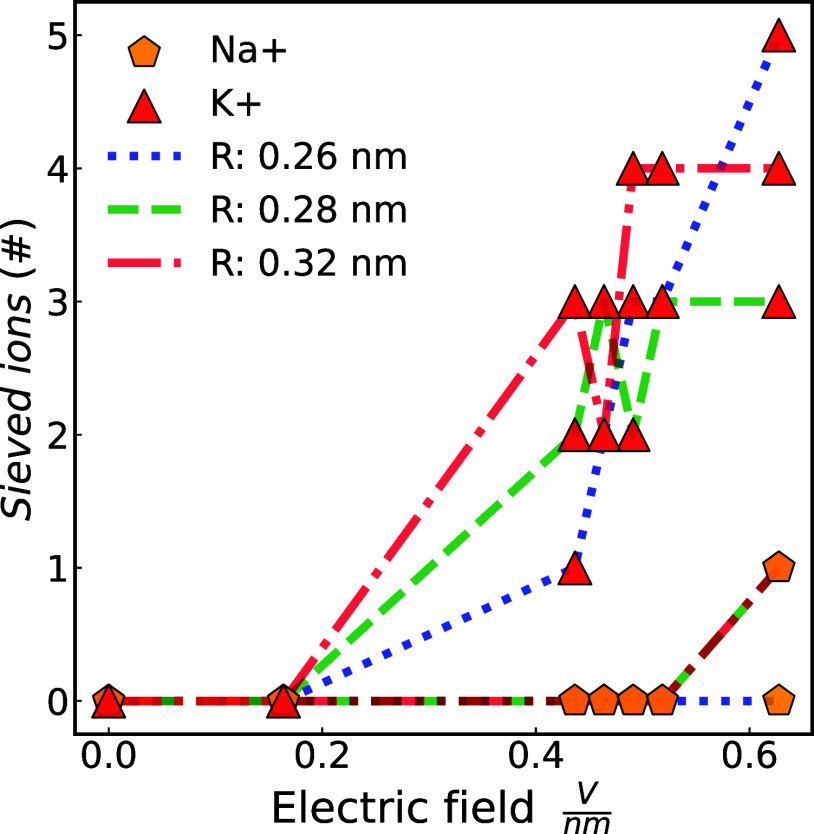
Number of sieved K^+^ (red pentagon) and Na^+^ (orange triangle) ions as a function of the electric field intensity for a graphene membrane with a nanopore of radii: 0.26 (blue), 0.28 (green), and 0.32 (red) nm. For a comparison of filtration results for a membrane with a nanopore radius of 0.26 nm under applied potentials of 3.2, 3.8, and 4.6 V using a different water model, refer to the Supporting Information.

When one or more cations traverse the membrane, they are drawn toward the anode by electrostatic attraction, resulting in changes to the electric field and potential difference. CPM-MD simulations (see Figure 10) revealed that graphene membranes with a nanopore size of 0.26, 0.28, and 0.32 nm completely rejected Cl^–^ anions (100%). The graphene membrane with a 0.26 nm nanopore radius showed high selectivity for K^+^ over Na^+^, sifting out 5 K^+^ ions without any Na^+^ leakage under a 0.63 V/nm electric field. Na^+^ ions struggled to permeate through any of the 0.26, 0.28, and 0.32 nm nanopores under electric fields weaker than 0.42 V/nm. PMF results showed the lowest energy barrier for K^+^ in the 0.32 nm nanopore membrane. The electric field-driven simulations indicated greater K^+^ ion flux for the 0.32 nm nanopore membrane under electric fields (*E*) between 0.18 and 0.52 V/nm, with the smallest flux for the 0.28 nm nanopore, consistent with PMF energy barriers. In the context of electric field-driven ion-sieving, the smallest nanopore membrane (0.26 nm) sieved the most K^+^ ions (5), followed by the 0.32 (4), and finally the 0.28 nm (3). Under electric fields lower than 0.42 V/nm, the membranes are largely ineffective, as both Na^+^ and K^+^ ions are unable to pass through the nanopore. When the electric field magnitude surpasses 0.52 V/nm, membranes with a nanopore radii greater than 0.26 nm, such as 0.28 and 0.32 nm, become ineffective in sieving K^+^ from Na^+^ ions, as they allow the permeation of both Na^+^ and K^+^ ions. Among all membranes tested *via* CPM-MD, the membrane with 0.26 nm nanopores exhibited the highest K^+^/Na^+^ selectivity under strong electric fields, whereas membranes with larger pores demonstrated enhanced K^+^ sieving efficiency at lower field strengths. The CPM-MD simulation results are consistent with previous CFM-MD studies,^27^ which employed electric field strengths of comparable magnitude and similarly reported that functionalized nanoporous graphene membranes favor the translocation of K^+^ over Na^+^, as K^+^ ions are able to traverse the pore under weaker electric fields.

The findings are in partial agreement with the results presented in Experimental Characterization through Water and Ion Transport across the Membrane section; both the CPM simulation results and the experimental data show an increase in analyte flux with increasing applied potential. Even though these findings are supported by prior experimental studies,^24^ which reported that functionalized graphene membranes with an average pore radius of 0.4 nm exhibit preferential K^+^ transport over Na^+^ at applied potentials of 0.3–0.6 V. This behavior is observed to a lesser degree in the fabricated nanoporous graphene (PCTE + Gr_900°C_ + UV/ozone + POSS) membranes, where partial Na^+^ leakage occurs due to a broader nanopore size and shape distribution, resulting in a slight ICS favoring K^+^ over Na^+^.

### Electric Field Influence on Ion Hydration and Water Molecules Structure

The number, coordination number (CN), and orientation of water molecules in the FHS are crucial factors in ion hydration.^66–68^ Previous studies highlighted ion dehydration as a method to reduce ion hydration and enhance ion transport in nanochannels by lowering ion coordination numbers through the removal of surrounding water molecules.^37,67,69^ To understand the effects of external fields on ion hydration shells, their influence on water structuring was examined. The radial distribution function (RDF) between species *i* and *j*, defined as *g*_*ij*_(*r*), measure how the density of oxygen atoms in water (O) varies as a function of distance from an ion or the oxygen atom in a water molecule. These RDFs provide insights into the spatial arrangement and structure of water molecules around ions, highlighting preferred distances and the degree of ordering within the FHS. As shown in Figure 11, the *g*_*ij*_(*r*) curves remain largely unaltered despite the presence of weak external fields (<1 V/nm), exhibiting negligible changes in peak location and height for both ions and oxygen atoms under an electric field of 0.63 V/nm, consistent with previous studies (see Table 1).

**Figure 11:**
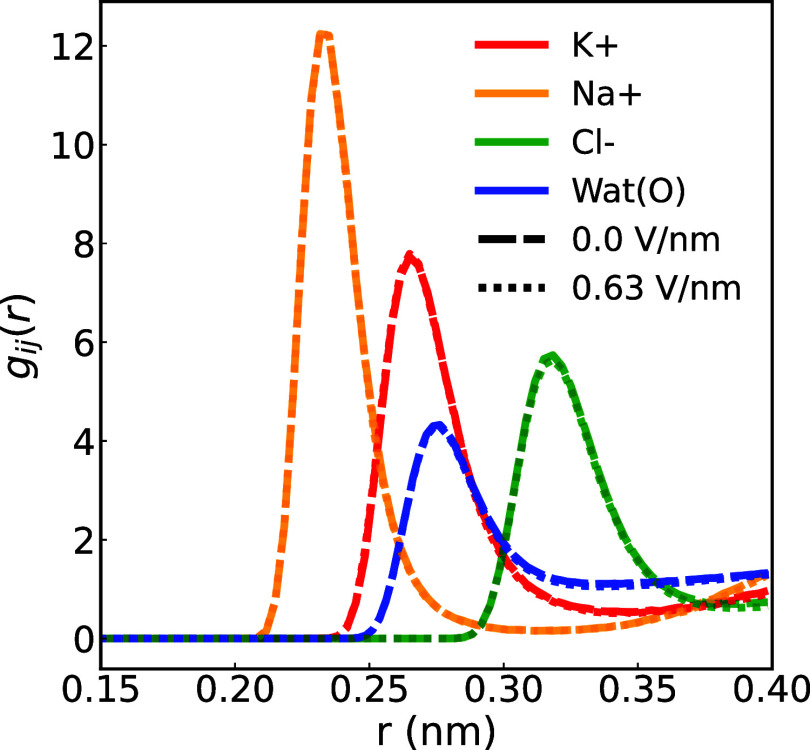
Ion-oxygen RDFs *g*_*ij*_(*r*) under external fields averaged across graphene membranes with radii of 0.26, 0.28, and 0.32 nm for K^+^ (i.e., *g*_OK^+^_ (r), red), Na^+^ (i.e., *g*_ONa^+^_ (r), orange), Cl^–^ (i.e.,., *g*_OCl^–^_ (r), green), and oxygen in water (i.e., *g*_OO_ (r), blue). Note that the curves for external fields 0 and 0.63 V/nm overlap. For the RDF of each ion or the oxygen atom in water molecules in each case and the results using a different water model refer to the Supporting Information.

**Table 1:** Positions (nm) of the First Peaks of the Atomic RDFs for Zero Electric Field^a^

*R*_firstpeak_	CPM-MD^2^	MD^3^	ab initio^4^
O–O	0.276	0.274	0.28
Na–O	0.235	0.238	0.238
K–O	0.268	0.271	0.278
Cl^–^-O	0.318	0.315	0.312

a Column 2: present estimates obtained in this work with CPM-MD; column 3: values obtained through conventional MD;^70–72^ column 4: values obtained through ab initio simulations in the absence of an applied electric field.^73–75^

A perturbation in the FHS of ions would manifest as reduced peak heights and amplified trough depths in the ions’ RDF, accompanied by augmented peak heights and diminished trough depths in the O–O *RDF*. This indicates water molecules transitioning into more densely packed configurations, leading to decreased interactions with ions. This phenomenon has been demonstrated in prior CFM-MD studies involving strong electric fields (>2 V/nm), posited as a mechanism for diminishing ion coordination number.^46^ However, the overlapping *g*_*ij*_(*r*) profiles in the absence and presence of a weak electric field for both oxygen atoms in water and ions suggests an unchanged FHS for the ions. To validate this, the average CN in the ions’ and water’s FHS was examined, with results shown in Figure 12. These findings agree with previous simulation and experimental studies (see Table 2) for ion–water solutions without an applied electric field. The coordination number remains unchanged under a weak electric field (0.63 V/nm), consistent with prior ab initio simulations.^73^

**Figure 12:**
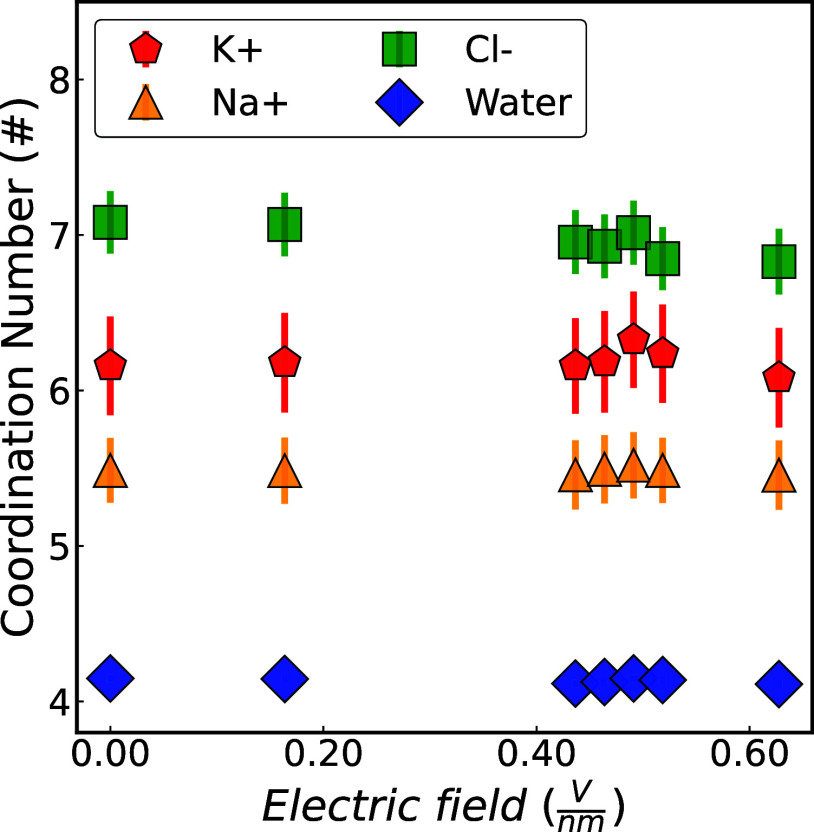
Coordination numbers in the FHS as a function of time averaged across graphene membranes with a radii of 0.26, 0.28, and 0.32 nm and under electric fields of magnitude 0.0, 0.16, 0.44, 0.46, 0.49, 0.52, and 0.63 V/nm for K^+^:O (red), Na^+^:O (orange), Cl^–^:O (green), O:O (blue). For the coordination number within the FHS of each ion or the oxygen atom in water molecules in each case and the results using a different water model, refer to the Supporting Information.

**Table 2:** Coordination Numbers (CN) from Various Methods for Zero Electric Field^a^

CN	CPM-MD^2^	MD^3^	ab initio^4^	Exp.^5^
O–O	4.2	4.2	4.7	4.3
Na–O	5.5	5.5	5.6	4.3–5.3
K–O	6.3	6.5	6.6	5.5–6.4
Cl^–^–O	7.0	5.7–7.0	5.8	5.3–6.9

a Column 2: present estimates obtained in this work with CPM-MD; column 3: values obtained through conventional MD;^70–72^ column 4: values obtained through ab initio simulations;^73,75^ column 5: values extracted from neutron and X-ray diffraction experiments.^71,76–80^

Given the negligible changes in the ions’ FHS, the structure between triplets of water molecules was examined using the triplet correlation function (TCF). The TCF of the oxygen atom in water molecules, *g*(O, O, O), was computed and averaged over applied electric fields (0.0, 0.46, 0.49, and 0.63 V/nm). As shown in Figure 13, the results indicate that water retains its tetrahedral structure even at high electric fields. Peaks at 60° (left panel) and 109° (right panel) correspond to icosahedral *g*(*r*, *r*, *r*) and tetrahedral *g*(*r*, *r*, *s*) triplet correlations, respectively, where the former reflects equilateral or isosceles triangle configurations and the latter involves *r* ≠ *s*. The structure of water molecules remains unperturbed under an electric field, as indicated by the high probability of observing the 109° angle (peak at *r* = 0.28 nm), which is 10 times higher than the 60° angle. This suggests that water avoids close-packed configurations at 0.63 V/nm,^81^ a phenomenon also observed by Dhabal et al.^82^ in water simulations without an electric field. For ions, the triplet correlation function *g*(O, ion, O) for Na^+^ shows a strong peak at 0.23 nm, indicating a preference for tetrahedral configurations due to strong electrostatic interactions with water (Figure 13). Both water and Na^+^ favor tetrahedral angles, while ions like K^+^ and Cl^–^ with weak hydration shells prefer icosahedral angles. Na^+^, with a strong hydration shell, consistently favors tetrahedral configurations, highlighting their significance for ions with semirigid hydration shells. Given the unaltered FHS configurations and water structure, the orientation of water molecules under an electric field was examined. In the presence of an electric field, hydrogen atoms align with the electric field, modifying water dipole orientation (see Figure 14). The analysis of the normalized frequency of each angle, denoted as Φ—the angle between the water dipole and the electric field vector—enabled the determination of the probability distribution characterizing the orientation of water molecules near the electrodes and in the bulk region of the simulation box. This angular distribution, spanning from 0 to 180° and segmented into equally sized 20° bins, is shown in Figure 14. Consistent with previous studies,^46,49,73,83^ the reorientation of water dipoles induced by external electric fields leads to a gradual decrease in the average angle with increasing field strength, while the angular distribution narrows, indicating stronger alignment of water dipoles with the field (see Figure 14). The shift in the Φ distribution from zero to 0.63 V/nm toward lower angles is smaller than that reported in CFM–MD simulations,^46,84^ but consistent with CPM–MD results,^51^ where water reorientation was less pronounced. In addition, the water Φ probability distribution exhibits a secondary feature (peak) centered at ∼90°, consistent with earlier simulations of ions in water under applied electric fields,^46^ whose intensity decreases as the field strength increases. The peak centered at ∼90° has been attributed to the random orientation of water molecules,^84^ which is most pronounced in the absence of an electric field. Furthermore, the orientation of water molecules within the ion’s FHS significantly influences ion hydration—a phenomenon known as anisotropic reorientation—which highlights the distinct dynamics of water molecules in the FHS compared to bulk water.^85^ Anions are surrounded by water molecules with one OH group pointing toward the anion, while for cations, the water oxygen atom faces the ion, with both hydrogen atoms oriented away, as depicted in Figure 15. For ions that exhibit strong electrostatic interactions with surrounding water molecules, a semirigid FHS forms.^85^ To evaluate water reorientation within the FHS, the probability distribution of the angle Φ—defined between the water dipole vector and the line connecting the ion center to the water oxygen atom—was computed, as shown in Figure 15. Weak electric fields minimally affect water molecule orientation within ions’ FHS. However, K^+^ shows more pronounced reorientation due to weaker hydration strength compared to Na^+^.^27^ The Cl^–^ ion shows a subtle shift in reorientation magnitude, indicating that its hydration strength is higher than that of K^+^ but lower than that of Na^+^. This reorientation alters the ionic hydration patterns, as evidenced by the shift in distances between the ions and the oxygen atoms of water in the FHS, as shown in Figure 16. Under a 0.63 V/nm electric field, K^+^ shows slight shifts toward greater distances and reduced peak heights, indicating increased water–water distances within the FHS. Na^+^ probability curves remain unchanged, while Cl^–^ shows a shift toward smaller distances due to closer proximity of the hydroxyl group to the anion. This structural transformation fosters a more pronounced formation of hydrogen bonds among water molecules within the FHS, as indicated by the probability distribution governing the residence time of water molecules, denoted as τ, within the FHS of the ions. The distribution for ions and water oxygen atoms is shown in Figure 17 involved assessing the count of water molecules persisting within the FHS of a specific ion across varying time intervals. This computation was subsequently averaged across all ions of the same type and normalized based on the cumulative frequencies. The probability distribution outlining the residence time for ions shows a reduction in the residence time of water molecules within the FHS shell of K^+^ under the influence of an electric field. In contrast, for the Na^+^ ion, with its amplified electrostatic interactions with water, the residence time decreases with a smaller gradient. Meanwhile, the residence time relating to the hydration shell of water molecules reaches a plateau value.

**Figure 13:**
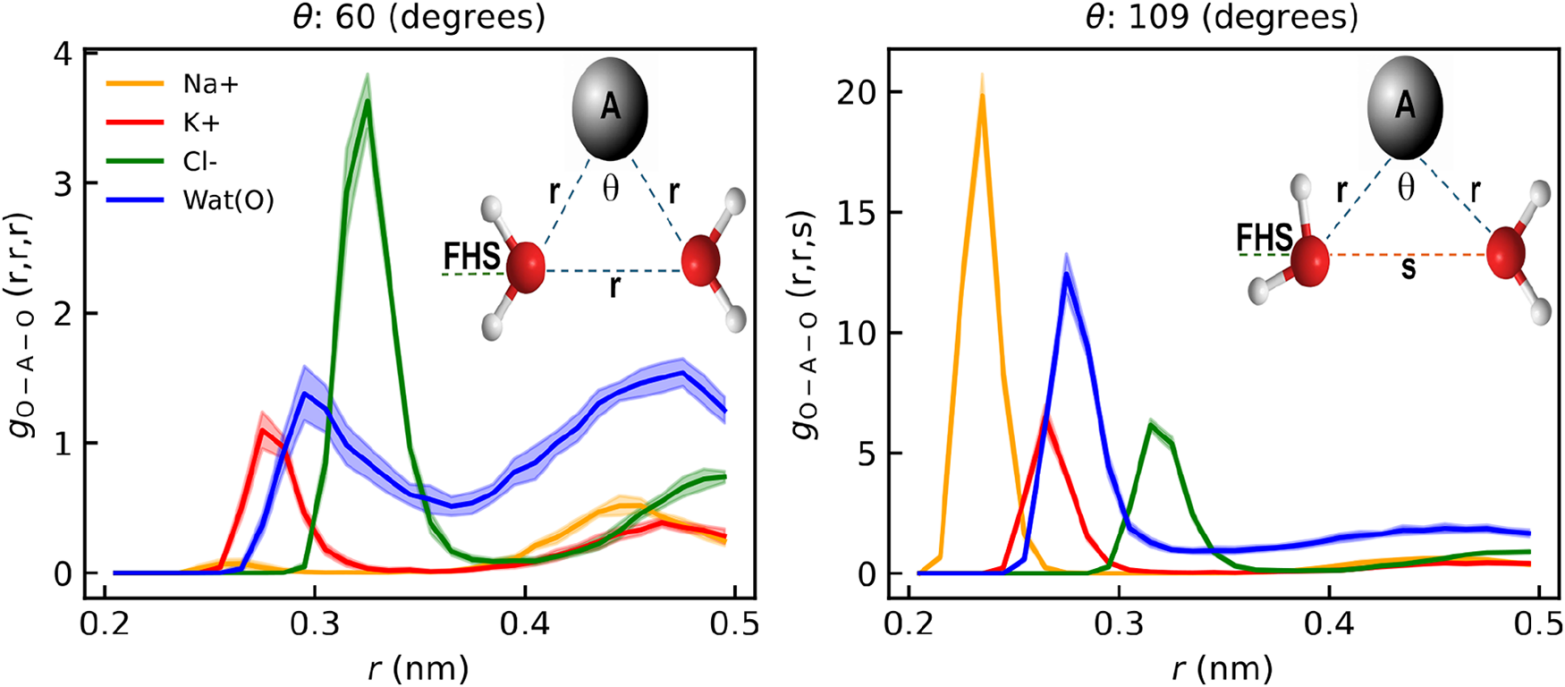
*g*(O, A, O) TCFs averaged across graphene membranes with a radii of 0.26, 0.28, and 0.32 nm and under electric fields of magnitude 0.0, 0.46, 0.49, and 0.63 V/nm, were A stands for Na^+^ (orange), K^+^ (red), Cl^–^ (green) and oxygen in water (blue). For the *g*(O, A, O) triplet correlation function of each ion or water molecule under different electric fields in every case and the TCFs across a range of angles from θ = 20° to θ = 160°, refer to the Supporting Information.

**Figure 14:**
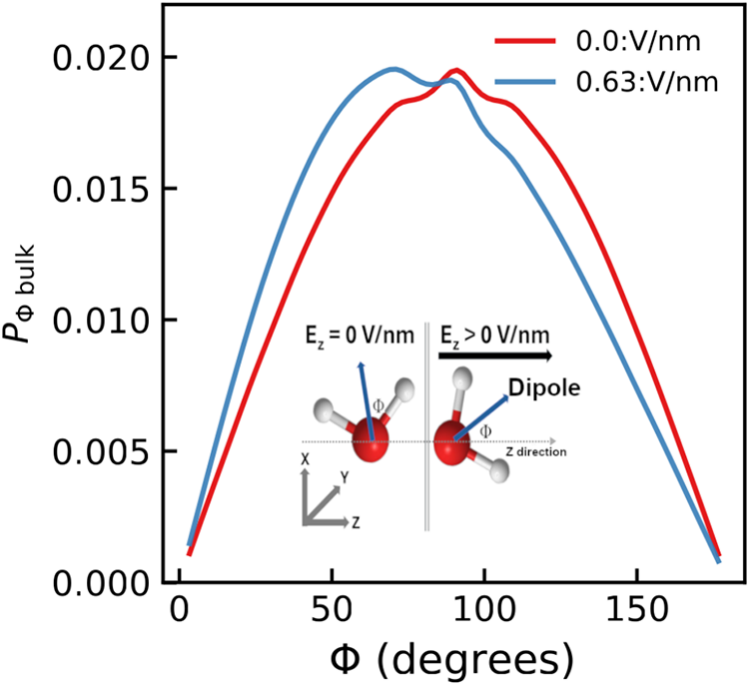
Probability distribution of the angle Φ between the water molecules dipole and the electric field vector (0,0,1) in the bulk of the simulation box (Φ_bulk_) averaged across graphene membranes with a radii of 0.26, 0.28, and 0.32 nm for water molecules under an electric field of magnitude 0.0 (red) and 0.63 (blue) V/nm. A schematic representation in the bottom of the panel shows the definition of angle Φ. For the probability distribution of the angle Φ between the water dipole moment and the electric field vector (0, 0, 1) in the regions near the cathode, anode, and graphene membrane, as well as results for each nanoporous graphene membrane of different radii under the electric fields studied, and further details on the determination of Φ, refer to the Supporting Information.

**Figure 15:**
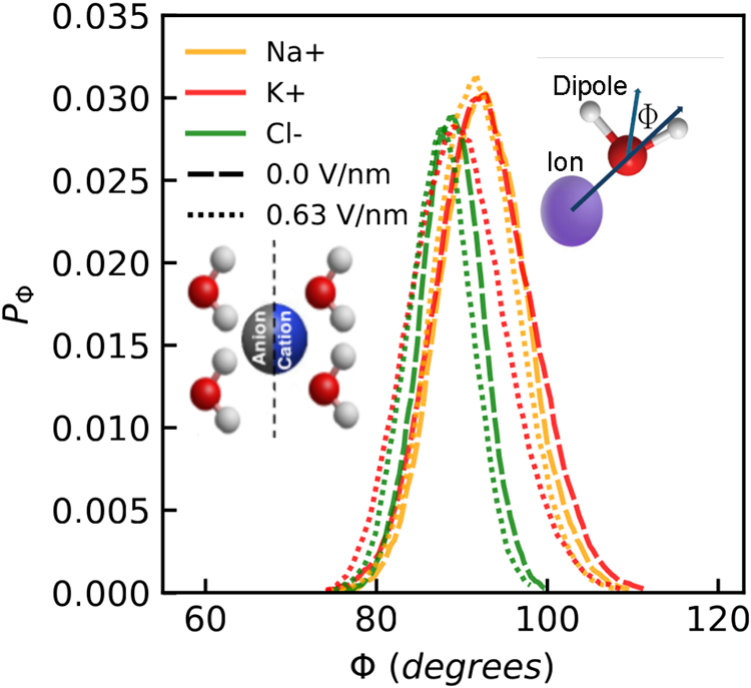
Probability distributions of the orientation angle (Φ) between the water molecules dipole and the vector between the ions oxygen atoms of water molecules in the FHS averaged across graphene membranes with a radii of 0.26, 0.28, and 0.32 nm for Na^+^ (orange), K^+^ (red), and Cl^–^ (green) under external fields. Note that the curves exhibit a shift to the left with the increase of the applied external fields between 0.0 (dashed) and 0.63 V/nm (dotted). A schematic representation shows the definition of angle Φ. For the Probability distribution of the orientation angle Φ in every case and the results using a different water model, refer to the Supporting Information.

**Figure 16:**
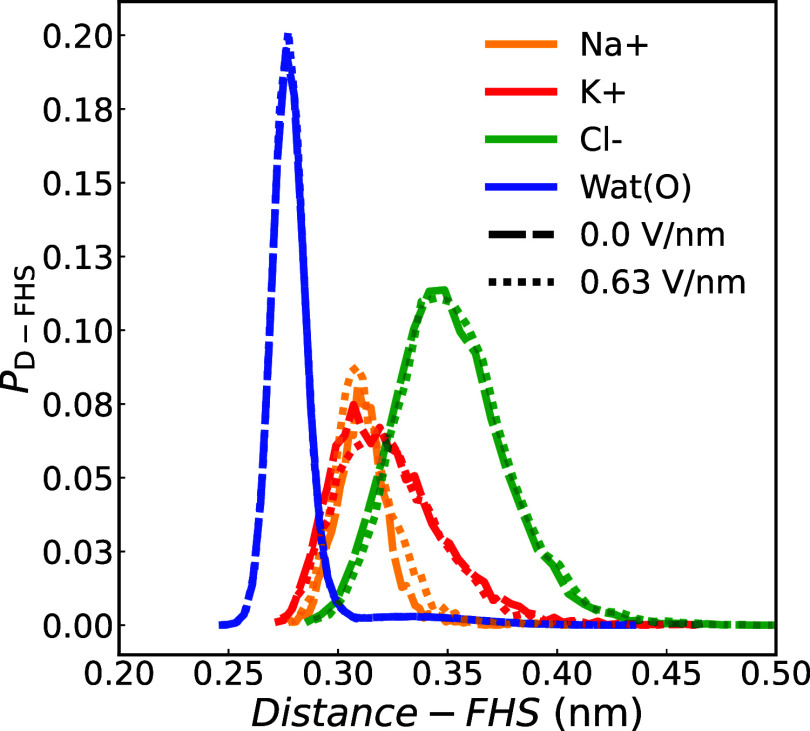
Distance probability between ions or oxygen in water molecules and the oxygen in other water molecules within the FHS, as a function of distance, averaged across graphene membranes with a radii of 0.26, 0.28, and 0.32 nm for Na^+^ (orange), K^+^ (red), Cl^–^ (green), and oxygen atom in water molecules under external fields. Note that the curves for external fields between 0 and 0.63 V/nm overlap. For the distance probability in each case and the results using a different water model, refer to the Supporting Information.

**Figure 17:**
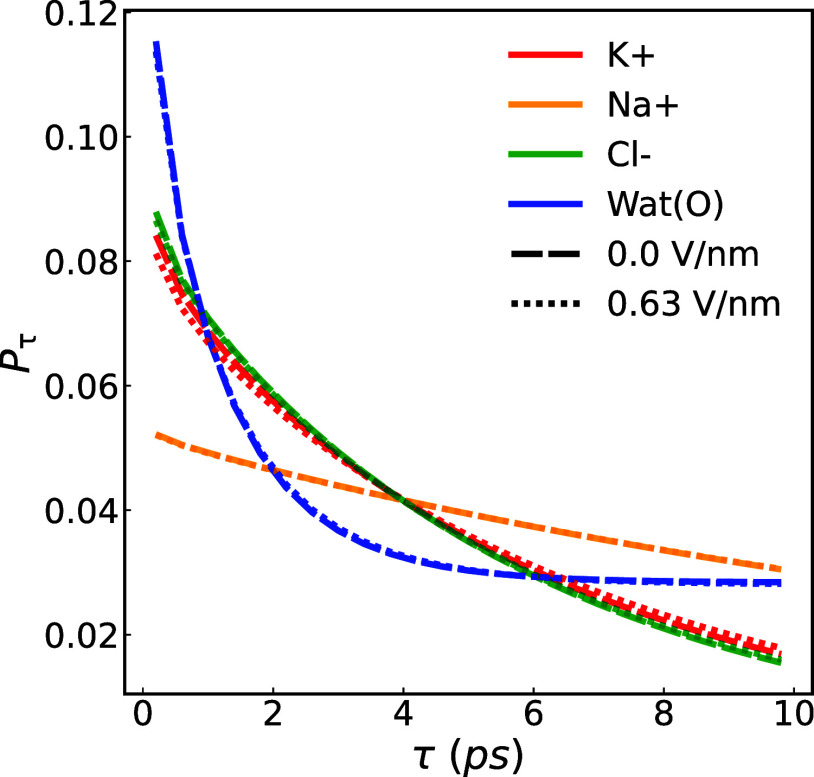
Probability for a water molecule to remain in the FHS of an ion or another water molecule as a function of residence time (τ), averaged across graphene membranes with a radii of 0.26, 0.28, and 0.32 nm for Na^+^ (orange), K^+^ (red), Cl^–^ (green), and oxygen atom in water molecules under external fields. Note that the curves for external fields between 0 and 0.63 V/nm overlap.

### Dynamic Behavior of Water–Ion Solutions under Electric Fields

To evaluate the effects of external fields on the dynamic properties of ionic hydration, we analyze the mean square displacement (MSD) of ions along the field direction (*z*-axis). When the field strength exceeds 0.0 V/nm, the MSD of the K^+^ and Cl^–^ ions along the *z*-direction (MSD_*z*_) experiences a pronounced enhancement, progressively intensifying with the increasing field strength. In contrast, the MSD_*z*_ of water and Na^+^ remains unperturbed under the influence of external fields, attributed to the inherent electroneutrality of water molecules and the strong electrostatic interactions of the Na^+^ ion.^46^ These changes indicate that in the presence of strong fields, K^+^ and Cl^–^ diffuse significantly faster within the solution, facilitating their escape from their original hydration shells.^19^ In the absence of an applied electric field, ions and their hydration shells can briefly move together during diffusion. However, under the influence of strong fields, the coordinated motions of ions and their adjacent hydration shells rapidly decouple, likely due to the weakened ion–water interactions (Figure 18).

**Figure 18:**
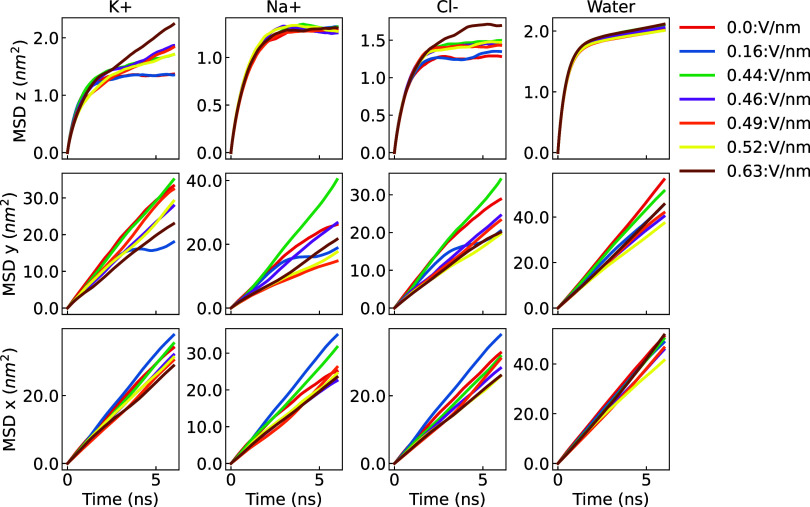
Mean square displacement (MSD) along the field direction (*z*, top panel), *y* (middle panel) and *x* (bottom panel) components averaged across graphene membranes with a radii of 0.26, 0.28, and 0.32 nm for K^+^, Na^+^, Cl^–^, and oxygen in water molecules under electric fields. For the individual *x*, *y*, and *z* components of the MSD in each case and the results using a different water model, refer to the Supporting Information.

Pronounced anisotropy between the *x*/*y* and *z* components of the mean squared displacement (MSD) is observed both in the presence and absence of an electric field applied along the *z*-direction (Figure 18). This anisotropy stems from spatial confinement in the nonperiodic *z*-direction, where the motion of water molecules and ions is restricted by the graphene membrane. The same behavior has been reported in ion–water simulations under confinement,^40^ where limited mobility along the confined axis leads to suppressed MSD_*z*_, regardless of whether an external electric field is applied. In the current simulation setup, the graphene membrane intensifies this effect by further hindering ion transport along the *z*-axis. As the electric field strength increases, ions—particularly K^+^—are more likely to traverse the membrane, leading to a reduction in MSD_*z*_ anisotropy and plateauing as shown in Figure 18. Minor anisotropy between the *x* and *y* components is also observed in Figure 18, but it is negligible compared to the confinement-induced effects along the *z*-direction, and is primarily attributed to ion–membrane/electrode and water–membrane/electrode interactions, as well as electric field-induced asymmetries.^86^ The self-diffusion coefficients (*D*), as depicted in Figure 19, were computed by fitting a linear equation to the various MSD profiles. The self-diffusion coefficients in the *x* (*D*_*x*_) and *y* (*D*_*y*_) dimensions remain largely unaffected when an external electric field is applied to the Na^+^, K^+^, and Cl^–^ ions. However, in the *z* dimension (*D*_*z*_), the self-diffusion coefficient increases as the magnitude of the applied external field rises for these ions. Notably, K^+^ ions display the highest sensibility to the external field, resulting in the most substantial increase in magnitude. This increase in magnitude can be attributed to the unique ability of K^+^ ions to traverse the nanopore, covering a greater distance compared to ions that are impeded by the nanopore. As a consequence, *D*_*z*_ exhibits an enhancement proportional to the applied external field’s strength.

**Figure 19:**
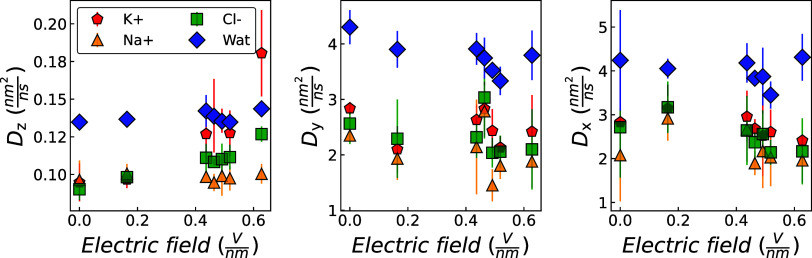
*D*_*z*_ (left panel), *D*_*y*_ (middle panel) and *D*_*x*_ (right panel) components of the self-diffusion coefficient (*D*) averaged across graphene membranes with a radii of 0.26, 0.28, and 0.32 nm and under electric fields of magnitude 0.0, 0.16, 0.44, 0.46, 0.49, 0.52, and 0.63 V/nm for K^+^, Na^+^, Cl^–^, and oxygen in water molecules under electric fields. For the individual *x*, *y*, and *z* components of the self-diffusion coefficient in every case, refer to the Supporting Information.

To further evaluate the displacement of K^+^ ions in contrast to Na^+^ ions, the instantaneous average velocity of each ion along the direction of the electric field (*z*-axis) was calculated over the course of the simulation. The resulting velocity distributions were then used to determine the ionic mobilities.^87^ For the full velocity distributions along the *z*-direction, refer to the Supporting Information. The ion’s electric mobility, μ_*q*_, defined by eq 5,^88^ was determined by extracting the *z*-component of the ion velocity in the presence of an electric field.^87^

5 $$\mu_{q}=\frac{V_{z}}{E}$$

Where *V*_*z*_ is the ion’s terminal drift velocity in the *z*-direction, and *E* is the magnitude of the electric field. The ion’s mobility was determined and is shown in Figure 20.

**Figure 20:**
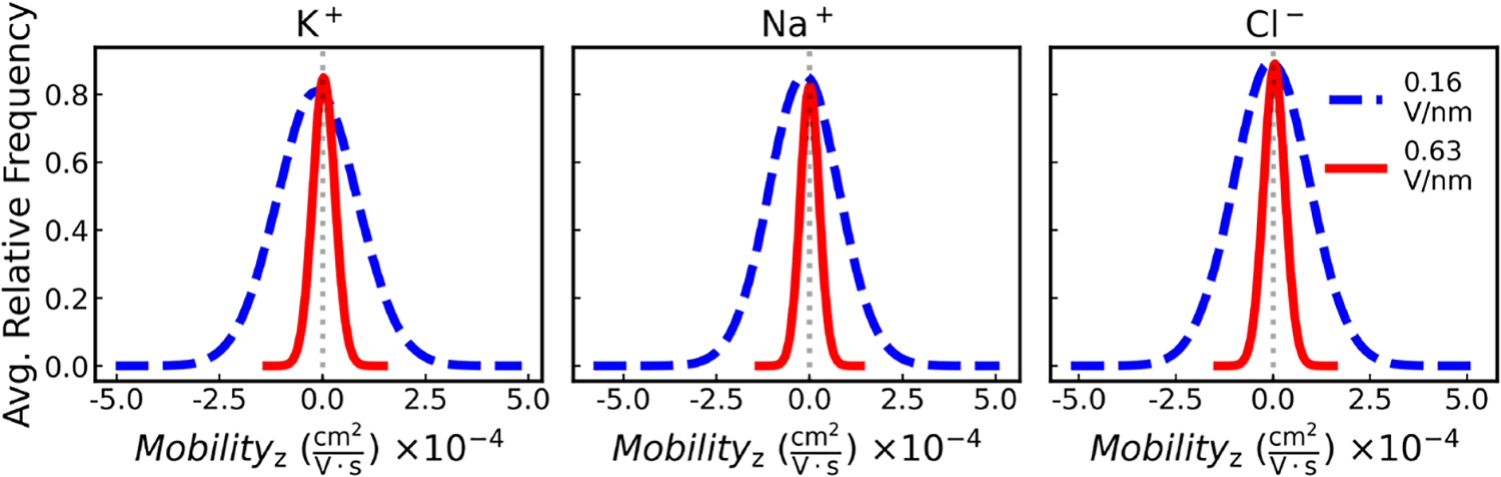
Average relative frequency as a function of ion mobility (i.e., ion mobility distribution) in the *z*-direction, μ_*q*_, averaged across graphene membranes with radii of 0.26, 0.28, and 0.32 nm, under electric fields generated by applied potential differences of 1.2 and 4.6 V. Results are shown for K^+^ (left panel), Na^+^ (middle panel), and Cl^–^ (right panel). For detailed ion mobility and terminal drift velocity distributions at each potential difference and membrane radius, as well as those obtained using a different water model, refer to the Supporting Information.

As shown in Figure 20 increasing the strength of the applied electric field—achieved by increasing the potential difference—leads to a noticeable narrowing and rightward shift in the ion mobility distribution, particularly for K^+^ ions. The peak of the distribution for K^+^ becomes sharper and more pronounced under higher field strengths, indicating enhanced mobility. In contrast, for Na^+^ and Cl^–^, although the distributions also become narrower, the peak height remains comparable to that observed under low field strength (e.g., *E* = 0.12 V/nm), and the rightward shift is less significant than that observed for K^+^. Moreover, K^+^ ions consistently exhibit a greater rightward shift and a more prominent peak in their mobility distribution compared to Na^+^ ions, suggesting a higher effective ionic mobility for K^+^ under the same electric field conditions, as reported in previous CFM-MD simulation studies.^24^ This behavior is generally attributed to the lower hydration energy of K^+^, which allows it to exit bulk water more readily than Na^+^.^27^

## Conclusions

In this study, we have established that weak electric fields, which do not induce highly ordered or ice-like phases in water, have minimal impact on the hydration shell (FHS) of Na^+^, K^+^, and Cl^–^. The structuring of water molecules around each ion is predominantly governed by the electrostatic interactions specific to each ion species. These interactions dictate the ease with which each ion can pass through the nanopore. We have also demonstrated that steric hindrance resulting from the dehydrated size of ions does not emerge as the dominant factor in species sieving. Ion mobility serves as a key descriptor of how the electric field influences the translational dynamics of ions. Rather than being driven by external constraints, the rigidity of an ion’s FHS arises from electrostatic interactions between the ion and its surrounding molecules. When considered alongside each ion’s hydration strength, these dynamics help explain the selective transport observed through the graphene nanopores. Specifically, the weaker hydration shell of potassium ions—resulting in easier dehydration—paired with their higher mobility, both relative to Na^+^ ions, contributes to the enhanced selectivity of K^+^ over Na^+^. Together, these effects result in differences in transport of K^+^ and Na^+^ ions under an applied electric field generated by a potential difference. Furthermore, the present work has showcased the reliability of the proposed methodology by leveraging nonequilibrium MD simulations in conjunction with the US technique and the CPM method for electric field-driven nanofiltration simulations. The results presented in this work offer qualitative, partial agreement with experimental findings while providing precise control over nanoporous membrane characteristics (specifically nanopore size), enabling the exploration of diverse materials, nanopore dimensions, pore size distributions, and different ions. These capabilities provide valuable insights into the characteristics and advantages of novel materials. Ultimately, our proposed methodology contributes to a fundamental understanding of ion transport and translocation mechanisms through nanopores, offering a framework that can be extended to both established and emerging membrane materials, as well as to a broader range of ionic species in solution.

Building on these insights, realizing the potential of nanoporous graphene membranes as biomimetic transport devices,^27^ voltage-tunable nanofluidic systems, and nanofiltration membranes capable of molecular sieving,^14,19,24^ requires precise control over the pore size distribution (PSD).^89^ The rational design of membranes with asymmetric nanopores and more representative pore-shape distributions,^90^ taking into account functional groups at pore edges that alter ion translocation under applied electric fields,^27^ as well as the pH-dependent charge sensitivity of graphene, where ionic environments can shift the charge state at pore edges,^14^ will help bring modeling and experiments into closer alignment, providing a stronger foundation for the practical design of nanoporous graphene membranes.

## Methods

### Experimental Methods

#### Membrane Fabrication

The methodology developed by Cheng et al.^28^ for the fabrication of nanoporous graphene membranes was followed to prepare the membranes used in this study. Nanoporous graphene was synthesized on Cu foils (catalyst) at 900 °C using low-pressure chemical vapor deposition (LPCVD) with H_2_ and CH_4_ (carbon source). Graphene was specifically synthesized at 900 °C since it forms high-density intrinsic subnanometer pores in the graphene lattice. The as-synthesized nanoporous graphene on Cu foil was then transferred onto polycarbonate track etch (PCTE) support via isopropanol-assisted hot lamination (IHL) method to ensure a high transfer yield (≥95%) and minimal surface contamination (no sacrificial polymer scaffold used). Subsequently, UV/ozone etching was performed to introduce new defects and enlarge existing nanopores in the graphene lattice. Finally, the large pores (>0.5 nm) and/or any tears were selectively sealed via interfacial polymerization (IP). IP was performed by introducing octa-ammonium-polyhedral-oligomeric silsesquioxane (POSS) and trimesoyl chloride (TMC). POSS in aqueous phase was introduced on the graphene side and trimesoyl chloride (TMC) in the hexane phase was introduced on the opposite side (PCTE side). Since TMC decomposes in water, POSS had to diffuse into hexane to react with TMC. The transport of POSS (∼0.5–1.8 nm) is sterically hindered through nanopores (<0.5 nm) in graphene because the shortest dimension of POSS is ∼0.5 nm (cage size), although the longest dimension of POSS is ∼1.8 nm. After IP, small nanopores in graphene (<0.5 nm) would remain intact, nanopores in the range of ∼0.5–1.8 nm would probably be sealed, whereas large nanopores (>1.8 nm), tears, and open pores would be completely sealed via POSS-polyamide (PA).

#### Diffusion-Driven Measurements

Diffusion-driven solute transport measurements across the fabricated membrane were performed using a customized 7 mL Side-Bi-Side glass diffusion cell (5 mm orifice), as shown in Figure 21. The membrane was installed between two diffusion cells with the graphene side facing the feed side (left cell), followed by clamping the cells in the diffusion system. During the measurement, the liquids in both cells were stirred vigorously at 1500 rpm with magnetic Teflon coated stir bars to minimize concentration polarization. Prior to diffusion-driven solute transport measurements, the system was rinsed with ethanol to fully wet the membrane, followed by washing with deionized (DI) water to completely replace ethanol. For measuring the salt transport, salt solution (0.5 M KCl or NaCl) was filled into the feed side while DI water was filled into the permeate side. A conductivity meter probe (connected to a Mettler Toledo SevenCompact S230 conductivity benchtop meter) was immersed in the permeate side to record the conductivity of the permeate solution every 15 s for 15 min (see Figure 21).^91–94^ The measured conductivity data were converted into concentration values using the conductivity–concentration calibration curve specific to each solute. The flow rate of each solute was computed via the slope of concentration change (concentration change per time) in the permeate side, while the normalized flux was calculated by dividing the slope of the fabricated membrane by that of the PCTE support membrane.

**Figure 21:**
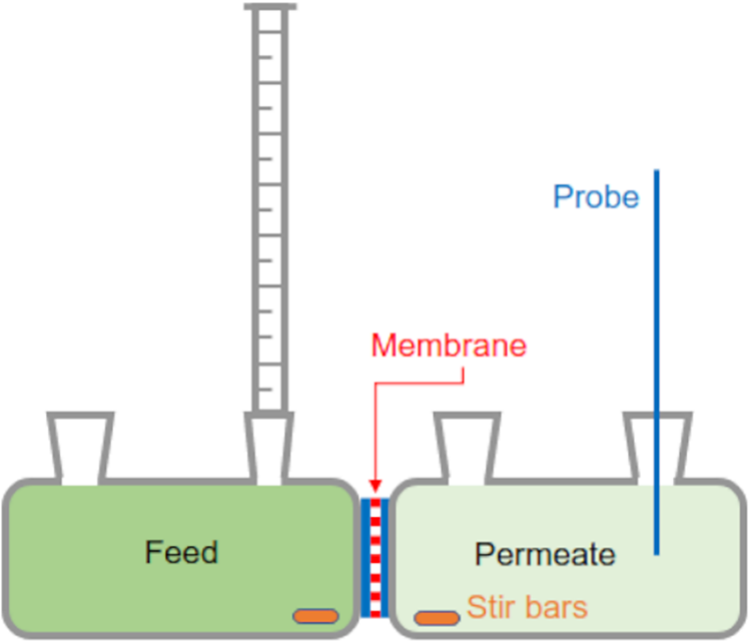
Experimental setup for measuring diffusion-driven solute transport, forward osmotic pressure-driven water transport, and salt (solute) rejection.

#### Water Transport Measurements

The same setup described in Diffusion-Driven Measurements section was used for the water transport experiments via forward osmosis, using glycerol ethoxylate (Sigma-Aldrich, 31694–55–0; average molecular weight *M*_*n*_ ∼ 1000) as the draw solution.^10,28^ The feed side was filled with DI water, followed by sealing with a rubber plug; while the permeate side was filled with draw solution. A digital camera was used to record the change of water meniscus level along the syringe (Figure 21). The osmotic pressure was calculated by the following relation:^10^ log ΔΠ = 4.87 + 0.8·(wt %)^0.34^ where ΔΠ is the osmotic pressure with the units of dyn/cm^2^. Experimentally, the water flux was computed using eq 6 ^91^

6 $$j_{\mathrm{water}}=\frac{\Delta V}{A\cdot \gamma \cdot \Delta t}$$

where Δ*V* is the change of water volume along the graduated syringe, *A* is the orifice area of the diffusion cell, γ is the porosity of PCTE support (9.4%), and Δ*t* is the measurement time. Water permeance was calculated by dividing representative water flux by corresponding osmotic pressure.^10,28^

#### Solute Rejection Measurements

The same setup described in Diffusion-Driven Measurements section was used for the solute rejection experiments conducted via forward osmosis, using glycerol ethoxylate (Sigma-Aldrich, 31694–55–0; average molecular weight *M*_*n*_ ∼ 1000) as the draw solution.^10,28^ For salt rejection experiments, the feed side was filled with a salt solution (16.6 mM KCl or NaCl) and sealed with a rubber plug, while the permeate side was filled with the draw solution.^10,28^ A conductivity meter probe was immersed in the permeate side to measure the conductivity change. The first method, referred to as method 1,^10^ determines the membrane’s solute rejection rate using eq 7

7 $$S_{\mathrm{rejection}}=\left[1-\frac{j_{\mathrm{solute}}/j_{\mathrm{water}}}{C_{f}}\right]\times 100$$

where *C*_*f*_ is the initial solute concentration on feed side, *j*_solute_ and *j*_water_ are solute flux and water flux, respectively. The solute (salt) flux (*j*_solute_) was calculated using eq 8 ^10,28,95^

8 $$j_{\mathrm{solute}}=\frac{\Delta C_{\mathrm{permeate}}}{\Delta t}\times \frac{V_{\mathrm{permeate}}}{A_{\mathrm{membrane}}}$$

where *j*_solute_ is the solute flux, $\frac{\Delta C_{\mathrm{permeate}}}{\Delta t}$ is the slope of the solute concentration change in the permeate side over time, *V*_permeate_ is the permeate volume, and *A*_membrane_ is the membrane open area. The second method, referred to as method 2,^96^ determines the solute rejection rate using eq 9

9 $$S_{\mathrm{rejection}}=\left[1-\left(\frac{C_{P}}{C_{F}}\right)\right]\times 100$$

where *C*_P_ is the solute concentration on permeate side after 24 h, and *C*_F_ is the initial solute concentration on feed side.

#### Ionic Conductance Measurements

The fabricated membranes were assembled in a H-cell for ionic conductance measurements, as shown in Figure 22. First the membranes were washed in ethanol for a total of 3 times (each with 5 min wait) to wet the membranes and get rid of any air/bubbles trapped. Thereafter the membranes were washed in water for 5 times (each at a interval of 5 min) to make sure all the ethanol is replaced with water. Finally, the membranes were washed with the respective electrolyte to be measured 0.1 M KCl or 0.1 M NaCl. The conductance measurement was done in a 4-electrode geometry as reported in previous publications.^28^ The voltage was scanned between ± 100 mV at a scan rate of 2 mV/s. The linear portion of the *I*–*V* curve was used for estimating the membrane’s resistance. Following the procedure described in previous studies,^24,60^ the intercation selectivity, ICS, (relative to K^+^) was defined following eq 10 ^60^

10 $${\mathrm{ICS}}_{i/K^{+}}=\frac{g_{i}}{g_{K^{+}}}$$

where *g*_*i*_ is the normalized conductance with respect to the bulk electrophoretic mobility ratio between cation *i* and K^+^ (e.g., *g*_K^+^_ for K^+^). A larger ICS indicates that cation *i* is more readily transported through the pore than K^+^.^60^ The normalized conductance *g*_*i*_ is defined in eq 11 ^24^

11 $$g_{i}=\frac{G_{i}}{\left(\frac{\mu_{i}}{\mu_{K^{+}}}\right)}$$

where *G*_*i*_ is the measured conductance through the graphene nanopores for cation *i*, and μ_*i*_ is the bulk electrophoretic mobility of cation *i*. For reference, the electrophoretic mobilities are μ_K^+^_ = 7.62 × 10^–4^ cm^2^ V^–1^ s^–1^ and μ_Na^+^_ = 5.19 × 10^–4^ cm^2^ V^–1^ s^–1^.^58^

**Figure 22:**
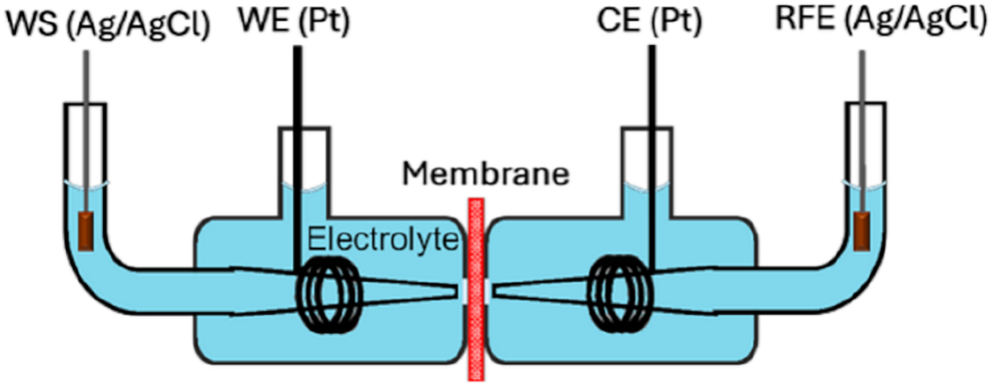
Schematic of H-cell for measuring the current–voltage characteristics.

### Computational Methods

#### System Setup

The systems, as illustrated in Figure 23 (top panel), were constructed using mbuild.^97^ They comprised five sections: a graphene wall, a saline water section (Feed reservoir), a nanoporous graphene membrane with a centrally located pore, and a pure water section (Permeate reservoir), followed by another graphene wall. The graphene walls and membrane were positioned parallel to the *xy* plane at the center of the simulation box. The pore was generated by selecting a reference carbon atom at coordinates (*x*_*r*_, *y*_*r*_, *z*_*r*_) and removing graphene atoms within a chosen pore radius based on their distance from the reference atom: $\sqrt{{\left(x-x_{r}\right)}^{2}+{\left(y-y_{r}\right)}^{2}}$ .^15^ The pore radii used were 2.2, 2.4, 2.6, 2.8, and 3.2 Å achieved by removing 5, 6, 7, 9, and 12 carbon atoms, respectively. The saline water section was positioned between the graphene membrane and a rigid graphene sheet, which served as a piston for applying external pressure and/or as electrodes. The graphene membrane, ions, and atoms of the graphene walls were represented using Lennard–Jones (LJ) electrostatic potentials with parameters from Cole and Klein.^98^ The water molecules were modeled using the nonpolarizable, rigid, extended point charge model (SPC/E),^99^ which is recognized for its reliability in reproducing the structure and dynamics of both bulk and confined water.^18,100^ The SPC/E model has been successfully employed to characterize water–ion interactions in desalination studies,^33,101^ as well as ion permeation through single graphene sheets with varying nanopore radii.^18,20,43^ Previous studies indicate that polarization effects are negligible in monovalent salt solutions,^18^ as the field associated with monovalent ions has minimal influence on water molecules within the hydration shell. The ions were modeled using the Loche et al.^102^ force field, which was optimized with respect to experimental solvation free energy and activity with water. This force field is fully transferable with the SPC/E, TIP3P,^103^ and TIP4P/2005 water models,^104^ accurately describing concentration-dependent density, ionic conductivity, and dielectric constant.^102^ Additional simulations were performed for graphene nanopores with a radius of 0.26 nm using both TIP3P and TIP4P/2005 water models. The *TIP3P* model—commonly used in desalination studies^105^ and ion permeation through graphene nanopores^15,17^ was paired with the Loche et al. ion force field. Simulations using the multisite TIP4P/2005 water model employed the Madrid/2019 ion force field.^106^ Lennard-Jones parameters for ion–water cross-interactions were derived using Lorentz–Berthelot combination rules, consistent with both SPC/E and TIP3P water models when used with the Loche et al. ion parameters. Similarly, nonbonded interactions for the TIP4P/2005 and Madrid/2019 combination are provided in the Supporting Information. All force field parameters were disseminated using Foyer.^107^

**Figure 23:**
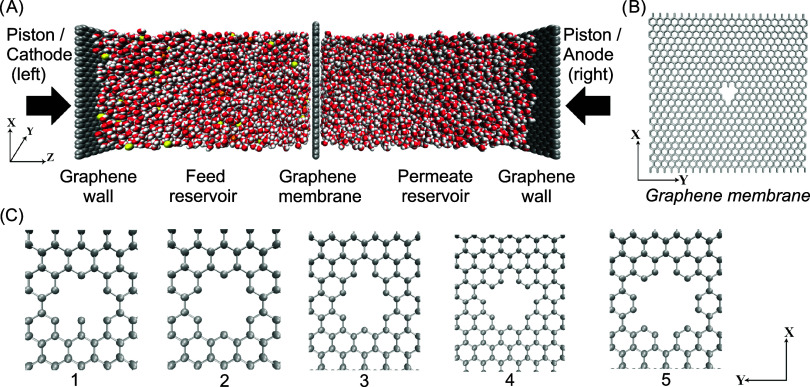
Panel (A) illustrates the simulation system for water desalination through a graphene membrane. The system consists of two chambers: the left chamber containing an aqueous NaCl solution with a concentration of 1.0 M NaCl (Feed reservoir), and the right chamber filled with a pure water bath (Permeate reservoir). The chambers are separated by the graphene membrane. The graphene plates, referred to as Piston left and Piston right, are subjected to hydraulic pressures P^left^ and P^right^, respectively. In the atomistic representation of the system, distinct atom types are color-coded: Cl^–^ (yellow), Na^+^ (orange), carbon atoms in graphene (silver) hydrogen atoms in water (white), and the oxygen atom in water (red). Panel (B) depicts the graphene membrane with a nanopore located at the center. The nanopore exhibits varying radii. Panel (C) provides schematic representations of the graphene nanopores with radii of 2.2, 2.4, 2.6, 2.8, and 3.2 Å, corresponding to the pores numbered 1 to 5, respectively.

#### Umbrella Sampling and Free Energy Profiles

The systems, illustrated in Figure 24, were constructed following the methodology outlined at the start of Computational Methods section. The system, with box dimensions of 58.13 × 2.87 × 2.81 nm^3^, consisted of two chambers: a feed reservoir containing water and a single ion pair, and a permeate reservoir containing only water. MD simulations were performed using GROMACS 2021 in double precision,^108^ with periodic boundary conditions (PBC) applied in the *x*, *y*, and *z* dimensions to maintain a continuous 2D membrane. The graphene membrane and walls were assumed to be rigid and fixed in their initial positions using a harmonic potential with a force constant of 10^5^ kJ mol^–1^ nm^–2^ to prevent membrane drift under applied forces. These restraints were applied throughout the pre-equilibration stages, except during compression and NPT equilibration, where atoms in the piston walls were only restrained in directions parallel to the membrane, allowing movement along the *z*-axis.^17^ The simulation methodology followed previous approaches using graphene walls as pistons to apply external pressure,^9,20,35,65^ as implemented in studies by Gupta et al.^65^ and Shen et al.^35^ Energy minimization was performed using the steepest descent method, followed by pre-equilibration under the NVT ensemble for 100 ps with a time step of 1 fs, employing a Berendsen weak coupling thermostat^109^ with a temperature fluctuation period of 0.2 ps. A second pre-equilibration stage involved a nonequilibrium MD (NEMD) simulation in the canonical ensemble, during which both pistons applied a pressure of 0.1 MPa for 1 ns using a 2 fs time step and the same Berendsen thermostat. Following compression, the system underwent a 1 ns presampling equilibration under the NPT ensemble with a 1 fs time step. Temperature was maintained at 298 K using a Nosé–Hoover thermostat^110–112^ with a 200 fs relaxation time, and pressure was controlled at 1 bar using a C-rescale barostat^113^ with a time constant of 1 ps and a compressibility of 4.5 × 10^–5^ bar^–1^.^17^ The equations of motion were integrated using the Velocity-Verlet algorithm,^114^ with neighbor lists constructed via the Verlet cutoff scheme.^115^ The geometry of water molecules was constrained using the LINCS algorithm.^116^ Short-range van der Waals and electrostatic interactions were truncated at 1.1 nm, and cross-interaction parameters for unalike atom pairs were calculated using Lorentz–Berthelot combining rules. Long-range electrostatic interactions were treated using the particle-mesh Ewald (PME) method^117^ with a Fourier spacing of 0.16 nm. The system temperature was maintained at 298 K throughout both equilibration and production stages.

**Figure 24:**
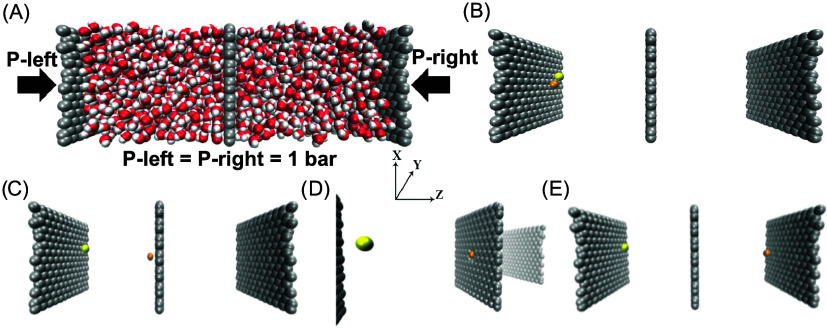
(A) Illustration represents the simulation system used for conducting US simulations. The setup consists of two chambers: the left chamber contains a NaCl ion pair, and the right chamber is filled with a pure water bath. These chambers are separated by a graphene membrane. In the atomistic representation of the system, distinct atom types are color-coded: Cl^–^ (yellow), Na^+^ (orange), carbon atoms in graphene (silver) hydrogen atoms in water (white), and the oxygen atom in water (red). (B––E). These images showcase the progression of the nonequilibrium pulling simulation for the Na^+^ ion at different time points, with water molecules excluded to enhance visual clarity.

The permeation pathways^17^ were generated by selecting an ion (K^+^, Na^+^, Cl^–^) or water molecule and then gradually pulling it the length of the simulation cell (4 nm) through the membrane in the *z*-direction (see Figure 24). The ion was pulled using the harmonic potential, *U*(*z*) = *k*_*z*_(*z* – *z*_eq_)^2^, where *z* is the coordinate of the ion, *z*_eq_ is the equilibrium coordinate of the potential and *k*_*z*_ is the force constant. The equilibrium coordinate *z*_eq_ was increased at a rate of 1.0 nm/ns for 5 ns and *k*_*z*_ = 1.0 × 10^4^ kJ/(mol nm^2^). The energy accumulated by the spring-like harmonic potential due to the deviation of the ion from the target position, *U* pull, is defined as the average of *U*(*z*) over the length of the entire pathway. Pathway simulations were conducted in the canonical ensemble. Since the harmonic potential was only applied to the position of the ion in the *z*-direction, the ion was able to freely move in the *x* and *y* directions. The graphene walls and membranes were assumed to be rigid during simulations and were kept restrained during the nonequilibrium pulling simulations. The free energy profiles (represented by PMF profiles) were calculated by US.^118–120^ Initial coordinates for the US windows were selected using coordinates generated from the pathway simulations, defined as the *z*-component distance between the center of mass (COM) of the ion/molecule and the graphene membrane. The reaction coordinate (ζ) is defined between the center of mass (COM) of the graphene membrane and the ion/molecule to ensure symmetry in the calculated PMFs across both sides of the nanopore.^43^ Configurations were chosen with two different umbrella window spacings in order to enhance the sampling around the graphene nanopore while at the same time ensuring good sampling overlap between adjacent simulation windows.^18,43,65^ The window spacing chosen for data collection is 0.1 nm from 1.5 to 1.0 nm away from the membrane and 0.05 nm from 1.0 nm to the membrane position, with the sampling being symmetrical, ensuring the same density of measurements before and after the membrane. In these simulations, the axial coordinate *z* of a single ion or molecule was subjected to a harmonic biasing potential. The bias potential constant, in kJ/(mol nm^2^), was 1 × 10^3^ for water, Na^+^, and K^+^, while for Cl^–^, it was 5 × 10^3^, consistent with previous MD studies^18^ were a higher biasing potential is used for Cl^–^. This higher bias for Cl^–^ was applied as it has been shown that Cl^–^ exhibits a slower convergence rate.^15^ During the calculation of the PMF, each window was simulated in the canonical ensemble for a total duration of 20.0 ns, where the initial 12 ns were considered as equilibration and were excluded from the analysis.^43,65^ Using the weighted histogram analysis method (WHAM),^121,122,121,122^ the PMF was calculated with a tolerance of 10^–6^. For additional details on the determination of the PMF profiles, refer to the Supporting Information.

#### Constant Potential Method (CPM)

The systems, illustrated in Figure 25, were built using the methodology outlined at the start of this section. The simulation box had dimensions 3.15 × 3.08 × 9.40 nm^3^. The system comprised two chambers: the feed reservoir and the permeate reservoir. Compared to the US simulations, the feed reservoir contained 20 Cl^–^, 10 Na^+^, and 10 K^+^ ions, corresponding to a 2.2 M concentration. This higher salinity, consistent with previous desalination studies,^20,33,123^ was selected to reduce computational cost without compromising accuracy. Prior work has shown that water flux is largely insensitive to ion concentration in the feed,^33^ and that ion concentration has a negligible impact on the energy profile associated with translocation through nanopores.^18^ MD simulations with completely neutral electrodes were initially performed using GROMACS 2021 in double precision,^108^ following the same equilibration protocol detailed in the previous section. The equilibrated system was used as an input for the CPM simulations, which were performed in LAMMPS^124^ with the CPM implemented via the ELECTRODE package^125^ in a slab geometry as previous CPM-MD. studies^126^ Although long-range electrostatic interactions are computed using different approaches—PME in GROMACS 2021 ^117,117^ and particle–particle–particle-mesh (PPPM) in LAMMPS^127^ Thielemann et al.^128^ demonstrated that no significant differences are observed between them, as PME is effectively an extension of PPPM. In this method, the charge fluctuations on the electrodes are driven by the local density fluctuations in the electrolyte solution confined between them. At each simulation step, the electric potential (ϕ_*i*_) of each atom in the electrodes is constrained to be equal to a constant applied external potential (Ψ), which is maintained over each respective electrode. The distinctive feature of CPM-MD is the calculation of electrode charges keeping electrodes at a desired electrostatic potential.^125^ In this slab geometry, we define the *z*-axis as the direction normal to the electrodes and apply periodic boundary conditions only in the *x*–*y* plane (parallel to the graphhene membrane). From this initial state, CPM-MD simulations were run for 40 ns with a 2 fs time step. For each nanopore radius, 6 potential differences were used (0.0, 3.4, 3.6, 3.8, 4.2, 4.6 Ψ). Bonds and angles in water molecules were constrained using the LINCS algorithm^116^ during the initial equilibration phase, as it employs a global convergence criterion that enhances stability under large-force conditions.^129^ Following the approach of previous CPM studies,^126,126^ the equilibrated configuration was then used in production simulations where constraints were applied using the SHAKE algorithm,^130^ which relies on a local convergence criterion and is more sensitive to high-force environments. The electric potential on each atom in the electrodes is constrained at each simulation step to be equal to a present applied external potential Ψ, which is constant over a given electrode.^51^ Constant NVT conditions are enforced using a Nosé-Hoover thermostat with a relaxation time of 200 fs and a temperature of 298 K. Non-Coulombic interactions were modeled with the Lennard–Jones potential with a cutoff of 1.2 nm, whereas Coulombic interactions were modeled using PPPM summation^127^ to a relative accuracy of 1 × 10^–6^. While traditional Ewald (PME) summation assumes periodic boundary conditions in all three dimensions, the slab-like geometry of the 2D periodic system was handled using the Ewald 2D (EW2D) solver from the ELECTRODE package.^125^ This method truncates the Coulomb interactions in real space, allowing it to be treated with a finite cutoff in a system lacking periodicity in the *z* dimension.^54,125^ The parameter of the Gaussian electrode charge distribution was set to 19.79 nm^–1^, as used in previous studies.^53,54^

**Figure 25:**
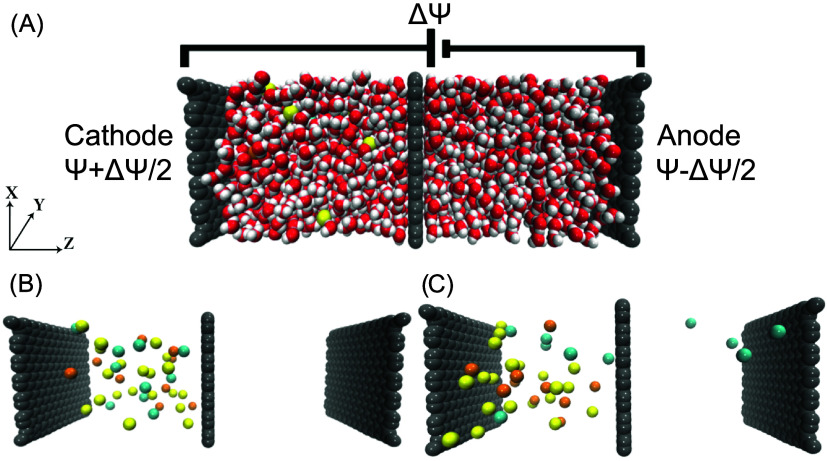
(A) This illustration depicts the simulation system utilized for conducting simulations using the CPM. The setup comprises two chambers: the left chamber containing an aqueous NaCl solution with a concentration of 2.2 M, and the right chamber filled with a pure water bath (permeate reservoir). These chambers are separated by a graphene membrane. The electrodes are represented by the graphene piston on the left (cathode) and the graphene piston on the right (anode). In the atomistic representation of the system, distinct atom types are color-coded: Cl^–^ (yellow), Na^+^ (orange), K^+^ (cyan), carbon atoms in graphene (silver), hydrogen atoms in water (white), and the oxygen atom in water (red). (B) Shows a representation of the initial system at time zero, with water molecules not shown for clarity. (C) Shows a representation of the system at the conclusion of the simulation. Again, water molecules are not shown for clarity.

## Supplementary Material



## Notes

detailed force field parameters and density profiles; umbrella sampling methodology; selectivity metrics; CPM analysis including electrostatic potential, electric field, MSD, electrical mobility, and water structuring; experimental details; and comparative results across different force fields encompassing PMF profiles, CPM outcomes, and mobility analyses (PDF)
