# Supplementary material for: Elucidating the Mechanisms of Ion Permeation through Sub-Nanometer Graphene Pores: Uncovering Free Energy Barriers via High-Throughput Molecular Simulations
Source: ACS Nano. 2025 Oct 17;19(50):42222–41. doi: 10.1021/acsnano.5c13306 (PMC12752709; doi:10.1021/acsnano.5c13306)
Supplement: Supplementary file 1 [file nn5c13306_si_001.pdf]

Supporting Information: Elucidating the Mechanisms of Ion Permeation through Sub-Nanometer Graphene Pores: Uncovering Free Energy Barriers via High-Throughput Molecular Simulations

Andres F. Ordorica ,[†] Peifu Cheng ,[†] Pavan Chaturvedi ,[†] Peter T. Cummings ,[*,†,‡] and Piran R. Kidambi [¶]

†*Department of Chemical and Biomolecular Engineering, Vanderbilt University, Nashville, Tennessee 37212, USA*

‡*School of Engineering & Physical Sciences, Heriot-Watt University, Edinburgh, UK*

¶*Department of Mechanical and Aerospace Engineering, University of Florida, Gainesville, Florida, 32611, USA*

E-mail: p.cummings@hw.ac.uk

Supporting Information

Experimental results

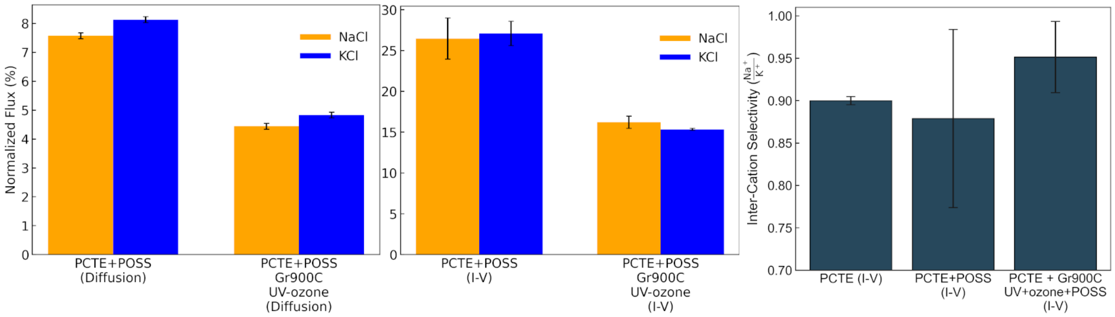

Figure S1: Normalized flux (%) values for the diffusion (*Diffusion*, left panel) and potential-driven (I–V, middle panel) salt transport experiments of NaCl (orange) and KCl (blue) and inter-cation selectivity (right panel) values for the potential driven (I–V) salt transport experiments of NaCl and KCl.

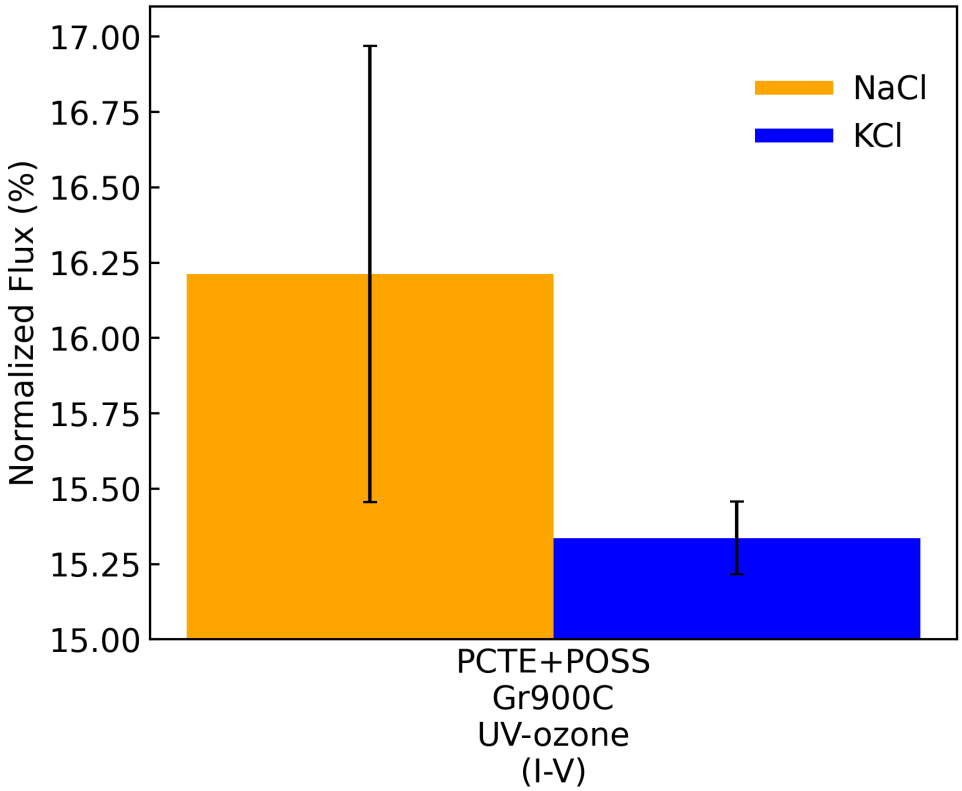

Figure S2: Normalized flux (%) values for potential-driven (I–V) salt transport experiments of NaCl (orange) and KCl (blue). The y-axis has been adjusted to enhance the clarity of the error bars.

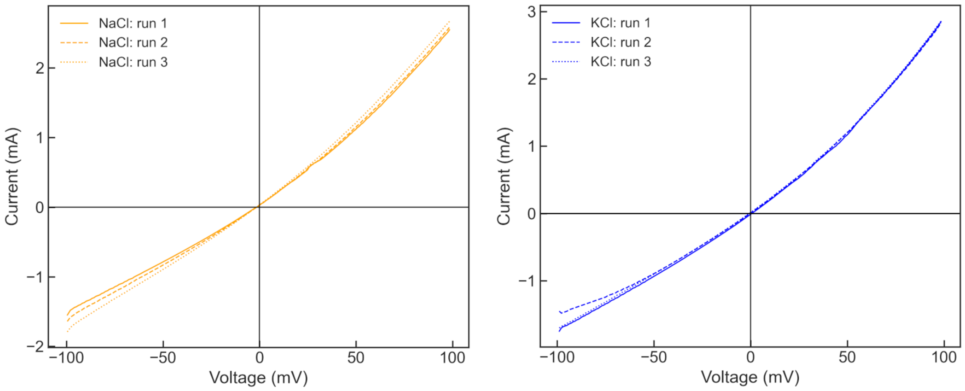

Figure S3: Current (mA) as a function of the applied potential difference (mV) values for potential-driven (I–V) salt transport experiments of NaCl (orange) and KCl (blue), carried out for three runs for each solution.

Force field parameters

The force field parameters used in this work are presented in the table S1.

Table S1: Force field parameters for water (*SPC/E*, *TIP3P*, and *TIP4P*/2005), graphene (Cole and Klein), and ions(Loche *et al.* and *Madrid*/2019).

Parameter type	Units	Value	Model	Reference
σ_{OO}	nm	0.3167	*SPC/E*	1
ϵ_{OO}	$\frac{\text{kJ}}{\text{mol}}$	0.6502	*SPC/E*	1
q_O	e	-0.8476	*SPC/E*	1
q_H	e	0.4238	*SPC/E*	1
r_{OH}	nm	0.1	*SPC/E*	1
θ_{H-O-H}	*degrees*	109.5	*SPC/E*	1
σ_{OO}	nm	0.31506	*TIP3P*	2
ϵ_{OO}	$\frac{\text{kJ}}{\text{mol}}$	0.63638	*TIP3P*	2
q_O	e	-0.834	*TIP3P*	2
q_H	e	0.417	*TIP3P*	2
r_{OH}	nm	0.09572	*TIP3P*	2
θ_{H-O-H}	*degrees*	104.52	*TIP3P*	2
σ_{OO}	nm	0.31506	*TIP4P*/2005	3
ϵ_{OO}	$\frac{\text{kJ}}{\text{mol}}$	0.7684	*TIP4P*/2005	3
q_O	e	0.0	*TIP4P*/2005	3
q_{OM}	e	-1.1	*TIP4P*/2005	3
q_H	e	0.550	*TIP4P*/2005	3
r_{OH}	nm	0.09572	*TIP4P*/2005	3
r_{OM}	nm	0.15	*TIP4P*/2005	3
θ_{H-O-H}	*degrees*	104.52	*TIP4P*/2005	3
σ_C	nm	0.337	Cole and Klein	4
ϵ_C	$\frac{\text{kJ}}{\text{mol}}$	0.3505	Cole and Klein	4
σ_{Na}	nm	0.2310	Loche *et al.*	2
ϵ_{Na}	$\frac{\text{kJ}}{\text{mol}}$	0.4500	Loche *et al.*	2
q_{Na}	e	1.0	Loche *et al.*	2
σ_K	nm	0.2830	Loche *et al.*	2
ϵ_K	$\frac{\text{kJ}}{\text{mol}}$	0.9000	Loche *et al.*	2
q_K	e	1.0	Loche *et al.*	2
σ_{Cl}	nm	0.4300	Loche *et al.*	2
ϵ_{Cl}	$\frac{\text{kJ}}{\text{mol}}$	0.4200	Loche *et al.*	2
q_{Cl}	e	-1.0	Loche *et al.*	2
σ_{Na}	nm	0.2210	*Madrid*/2019	3
ϵ_{Na}	$\frac{\text{kJ}}{\text{mol}}$	1.472	*Madrid*/2019	3
q_{Na}	e	0.85	*Madrid*/2019	3
σ_K	nm	0.230	*Madrid*/2019	3
ϵ_K	$\frac{\text{kJ}}{\text{mol}}$	1.985	*Madrid*/2019	3
q_K	e	0.85	*Madrid*/2019	3
σ_{Cl}	nm	0.469	*Madrid*/2019	3
ϵ_{Cl}	$\frac{\text{kJ}}{\text{mol}}$	0.076	*Madrid*/2019	3
q_{Cl}	e	-0.85	*Madrid*/2019	3

The non-bonded cross-interaction parameters used for the *Madrid*/2019[3] and *TIP4P*/2005[5] force fields are presented in table S2, if parameters are not explicitly listed, LB combination rules were used to determine them.

Table S2: Cross-interaction non-bonded parameters for water (*TIP4P*/2005) and ions(*Madrid*/2019). Ow represents the oxygen atom in water.

Parameter type	Units	Value	Model	Reference
σ_{Na-Ow}	nm	0.260	*Madrid*/2019	3
ϵ_{Na-Ow}	$\frac{\text{kJ}}{\text{mol}}$	0.793	*Madrid*/2019	3
σ_{K-Ow}	nm	0.289	*Madrid*/2019	3
ϵ_{K-Ow}	$\frac{\text{kJ}}{\text{mol}}$	1.4	*Madrid*/2019	3
σ_{Cl-Ow}	nm	0.423	*Madrid*/2019	3
ϵ_{Cl-Ow}	$\frac{\text{kJ}}{\text{mol}}$	0.061	*Madrid*/2019	3
σ_{Na-Cl}	nm	0.300	*Madrid*/2019	3
ϵ_{Na-Cl}	$\frac{\text{kJ}}{\text{mol}}$	1.438	*Madrid*/2019	3
σ_{K-Cl}	nm	0.339	*Madrid*/2019	3
ϵ_{K-Cl}	$\frac{\text{kJ}}{\text{mol}}$	1.400	*Madrid*/2019	3

Density profiles

The density profile along the z-direction was computed after the NPT equilibration stage and prior to the sampling simulations for the systems used in the *US* calculations. The systems were parameterized using the Loche *et al.* force field for the ions and the *SPC/E* model for water. For the membrane with a nanopore radius of 0.26 nm, the z-direction density profiles of additional systems parameterized using Loche *et al.* and *TIP3P* for the ions and water molecules, respectively, as well as *Madrid*/2019 and *TIP4P*/2005 for the ions and water molecules, respectively, are also included for comparison. These systems are shown in Fig.S4.

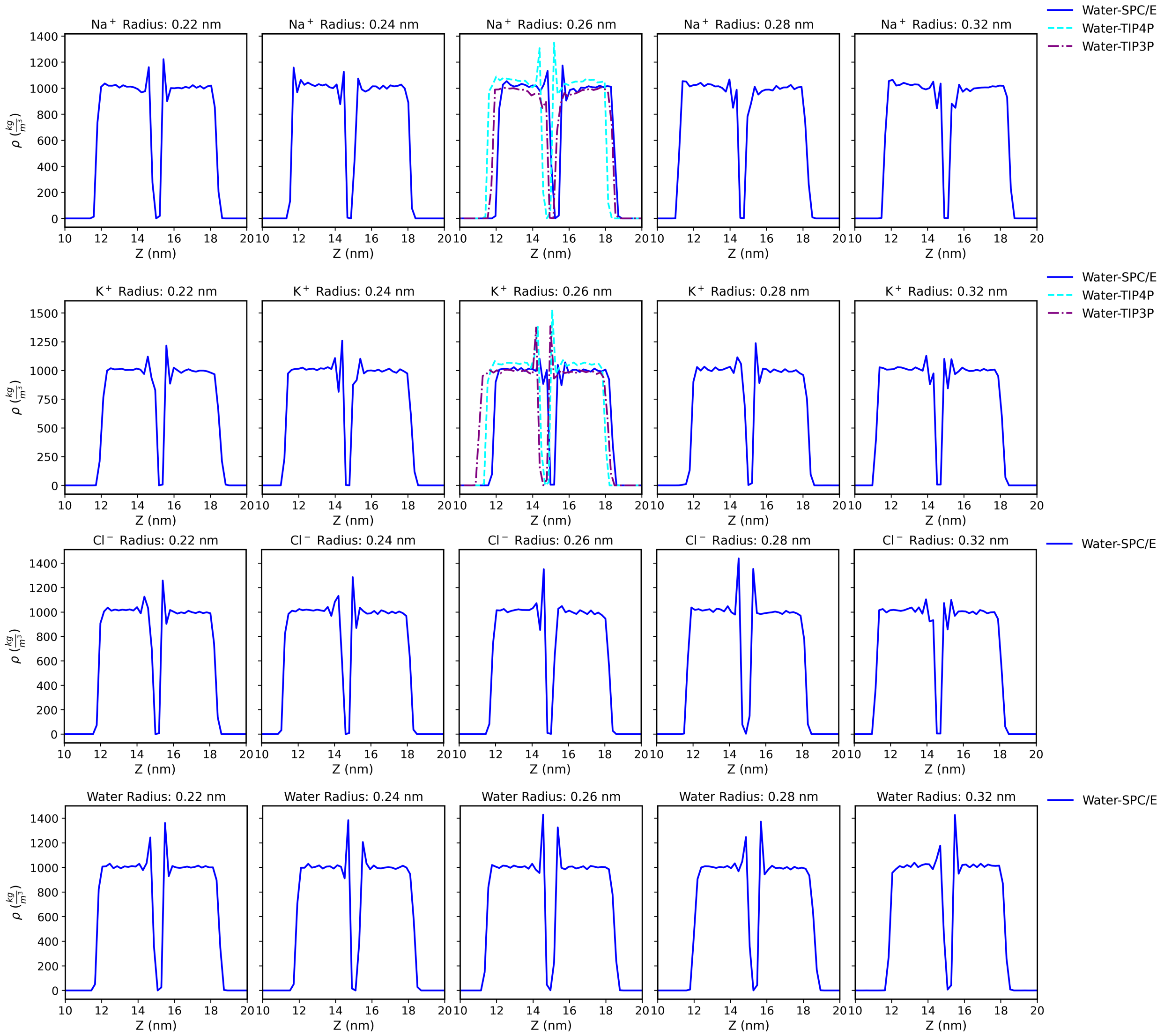

Figure S4: Density profiles along the z-direction for graphene nanopore systems with radii of 0.22, 0.24, 0.26, 0.28, and 0.32 nm, parameterized using the Loche *et al.* force field for ions and the *SPC/E* model for water. For the 0.26 nm radius system, additional profiles are shown for systems parameterized with Loche *et al.* and *TIP3P*, as well as *Madrid*/2019 and *TIP4P*/2005, for ions and water molecules, respectively.

The density profile along the z-direction was computed after the NPT equilibration stage and prior to the *CPM* simulations. The systems were parameterized using the Loche *et al.* force field for the ions and the *SPC/E* model for water. For the membrane with a nanopore radius of 0.26 nm, the z-direction density profiles of additional systems parameterized using Loche *et al.* and *TIP3P* for the ions and water molecules, respectively, as well

as *Madrid*/2019 and *TIP4P*/2005 for the ions and water molecules, respectively, are also included for comparison. These systems are shown in Fig.S4.

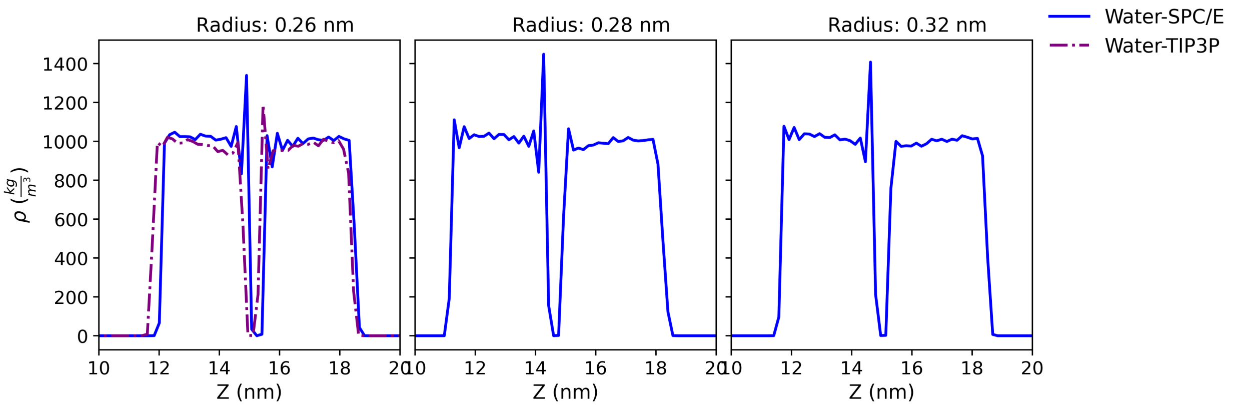

Figure S5: Density profiles along the z-direction for graphene nanopore systems with radii of 0.26, 0.28, and 0.32 nm, parameterized using the Loche *et al.* force field for ions and the *SPC/E* model for water. For the 0.26 nm radius system, additional profiles are shown for systems parameterized with Loche *et al.* and *TIP3P* for ions and water molecules, respectively.

Umbrella sampling

The ions and water molecule were pulled along a single reaction coordinate (ζ) defined between the center of mass (COM) of the ion or water molecule and that of the graphene membrane. By this definition, the ion or water molecule is positioned at 0 nm on the reaction coordinate when located at the center of the nanopore. The pulling was performed along the z-axis using a direction-type geometry (pulling in the direction of the graphene membrane) with a positive harmonic biasing potential. Initial configurations for the *US* simulations were generated by placing the ion at various positions along the reaction coordinate (ζ) relative to the graphene membrane. A total of 54 configurations were simulated for 20 ns each. The first 12 ns of each trajectory were discarded as equilibration, and the remaining data were used to compute the *PMF* profile using the weighted histogram analysis method (WHAM[6]). The $g_w ham$[7] implementation of WHAM was employed to determine the *PMF*s for both the ions and the water molecule. For *PMF* calculations, the reaction coordinate was divided

into 120 equally spaced bins, each approximately 0.025 nm wide. The PMF was considered symmetric ($g_{w}ham$ option -sym YES) at the default value of 0 nm, corresponding to the ion or water molecule being at the center of the nanopore. The overlap between the different simulated windows and the resulting PMF profile can be observed in Fig. S6.

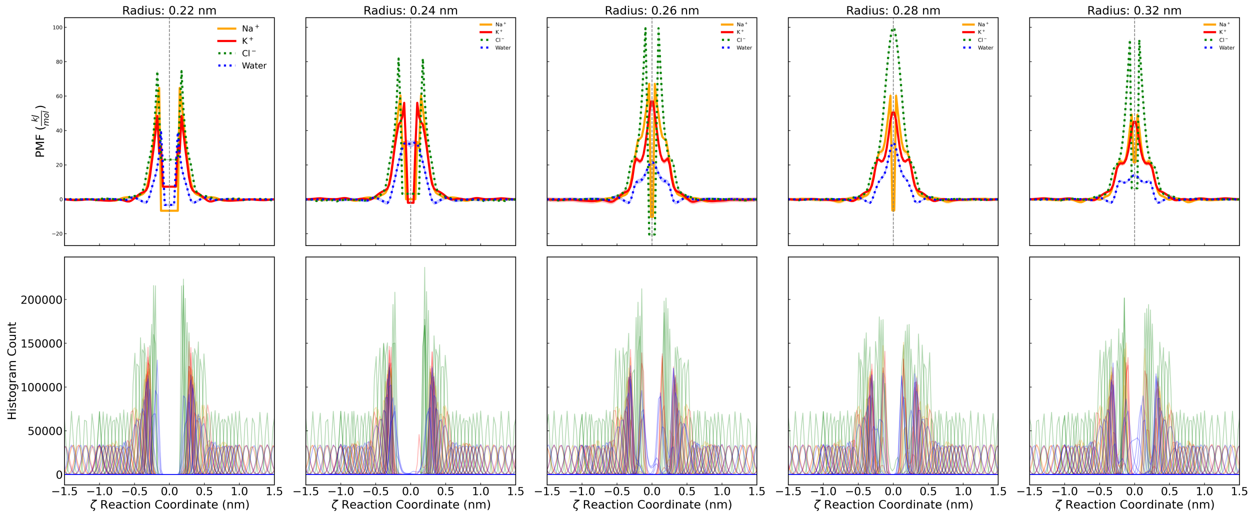

Figure S6: Potential of mean force (top panels) and histogram count (bottom panels) of K^+ (red), Na^+ (orange), Cl^- (green), and water molecule (blue) as a function of the reaction coordinate (ζ) for the species gaining passage through a membrane with a graphene nanopore of radii 0.22, 0.24, 0.26, 0.28 and 0.32 nm.

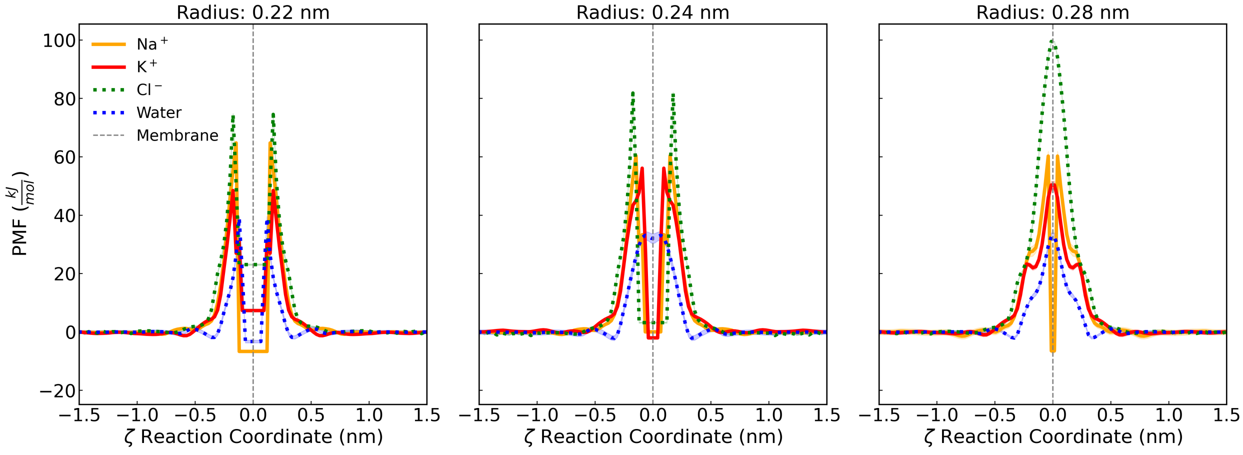

Figure S7: PMFs of K^+ (red), Na^+ (orange), Cl^- (green), and water molecule (blue) as a function of the reaction coordinate (ζ) for the species gaining passage through a membrane with a graphene nanopore of radii 0.22, 0.24, and 0.28 nm.

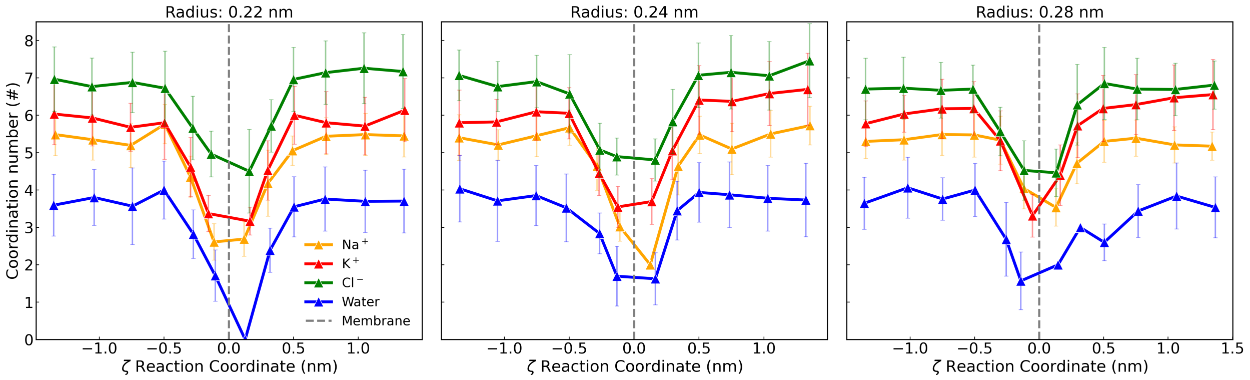

Figure S8: K^+: O (red), Na^+: O (orange), Cl^-: O (green) , O:O (blue) coordination numbers in the FHS as a function of the reaction coordinate (ζ) for the species gaining passage through a graphene membrane with a nanopore of radii 0.22, 0.24 and 0.28 nm

Selectivity

For the values of the natural logarithm of the selectivity reported in the main text, the corresponding non-logarithmic selectivity values are presented in Fig.S9.

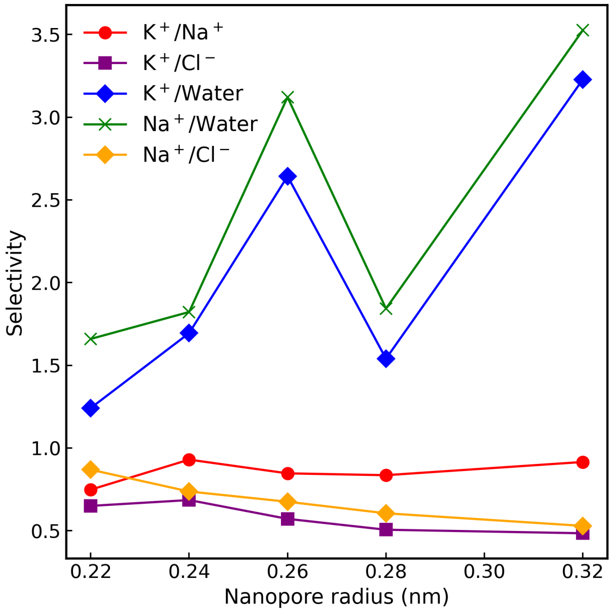

Figure S9: graphene membrane selectivity as a function of the nanopore radii for K^+/Na^+ (red), K^+/Cl^- (purple), Na^+/Cl^- (orange) , K^+/water (blue), and Na^+/water (green).

CPM

Electrostatic potential

Following the approach used by Lin *et al.*[8] for solving the electrostatic potential in a system with a slab geometry using Poisson's equation 1,

$$\frac{d^2\psi(z)}{dz^2} = -\frac{\rho(z)}{\epsilon_0} \tag{1}$$

Where $\psi(z)$ is the electrostatic potential along the direction z, $\rho(z)$ is the atom charge density along the z direction, and ϵ_0 is the vacuum permittivity. Integrating once gives equation 2,

$$\frac{d\psi(z)}{dz} = -\frac{1}{\varepsilon_0}\int_0^z \rho(z')\,dz' + C_1 \tag{2}$$

The second integration gives equation 3,

$$\psi(z) = -\frac{1}{\varepsilon_0}\int_0^z \int_0^{z''} \rho(z')\,dz'\,dz'' + C_1 z + C_2 \tag{3}$$

This gives a 1 dimension form of Poisson's equation integrated twice. The electrostatic potential $\psi(z)$ is determined by integrating the charge density $\rho(z)$ twice with respect to z. To fully specify the electrostatic potential in the system, we apply two boundary conditions: (1) The reference point is at z=0, which means $\psi(z) = 0$ at z = 0 (2) The electric field past the most right boundary is zero, given by equation 4,

$$E(z)\big|_{z\to\infty} = -\frac{d\psi(z)}{dz}\bigg|_{z\to\infty} = 0 \tag{4}$$

The first boundary condition results in $C_2 = 0$, and the second condition is satisfied because the system is charge-neutral, which translates into $C_1 = 0$ by equation 5:

$$\int_0^\infty \rho(z')\,dz' = 0 \tag{5}$$

This method of solution assumes planar symmetry, due to the consideration of a slab system, where the potential only varies along the z dimension and is assumed uniform in the x and y (parallel to the membrane and electrodes).[8,9]

The resulting electrostatic potentials computed for the systems investigated in this study are shown in Fig.S10.

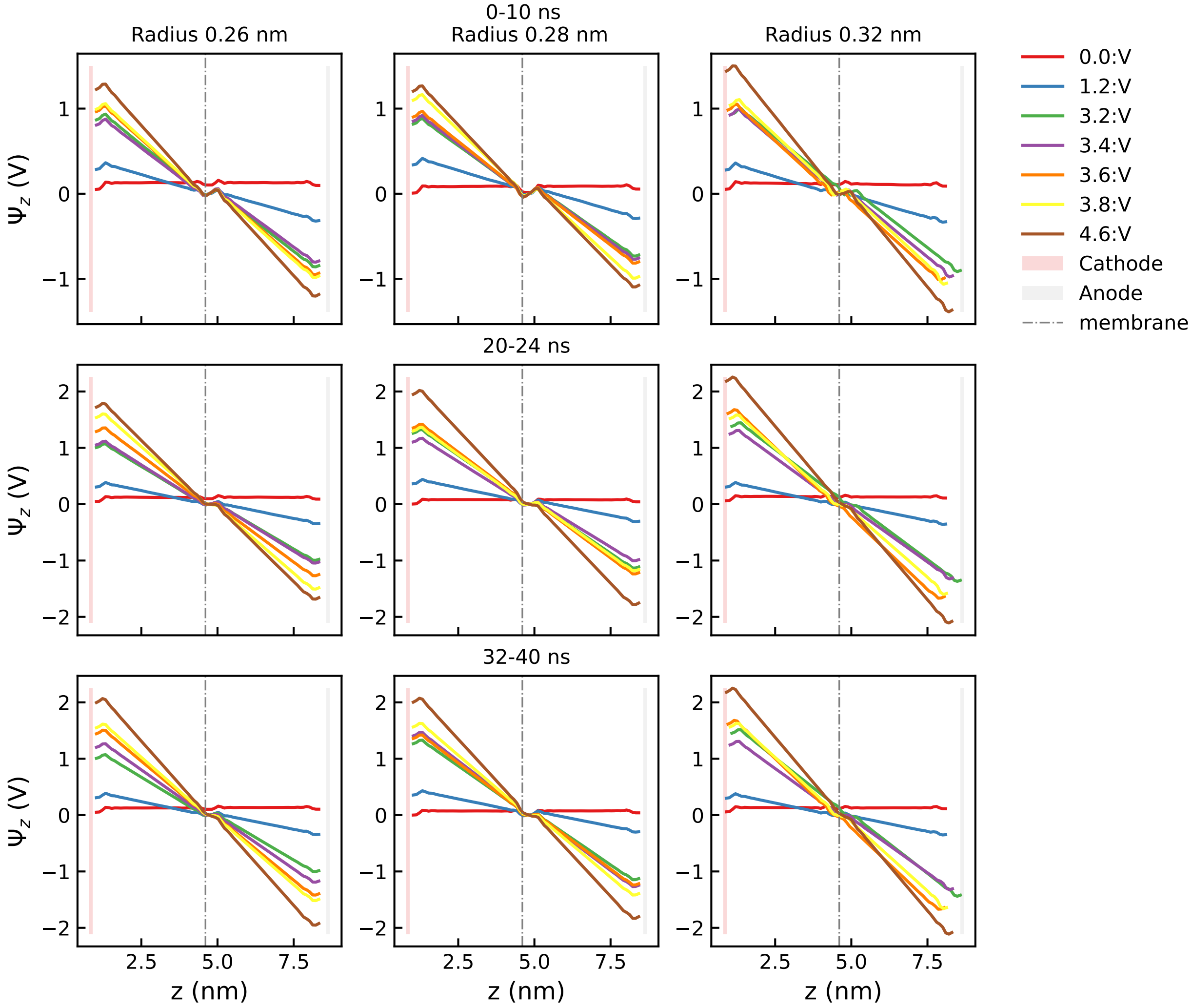

Figure S10: Electric potential profiles for the application of 0.0, 1.2, 3.2, 3.4, 3.6, 3.8 and 4.6 Ψ and potential differences on the graphene electrodes for a graphene membrane of nanopore radii 0.26 (first column), 0.28 (second column), and 0.32 (third column) nm for the simulation times 0-10 (first row), 20-24 (second row), and 32-40 (third row) ns . The gray shaded region represents the anode, the red shaded region the cathode, and the dotted vertical line the position of the graphene membrane

Electric field

For the one-dimensional electrostatic potentials determined for the systems, the electric field along the z-direction was obtained by taking the gradient of the electrostatic potential, as shown in Fig.S11.

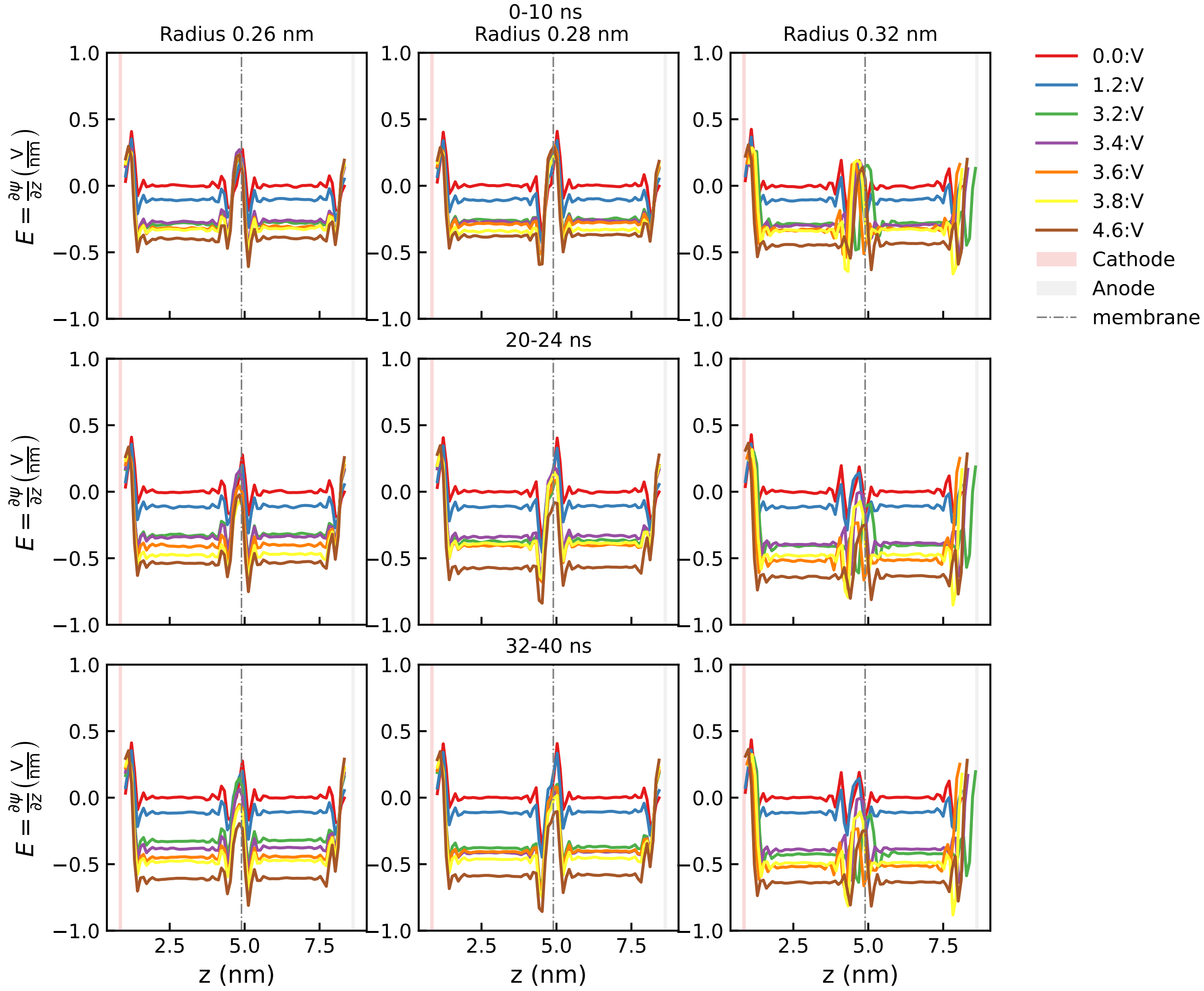

Figure S11: 1D electric field profiles along the z-direction for the application of 0.0, 1.2, 3.2, 3.4, 3.6, 3.8 and 4.6 Ψ and potential differences on the graphene electrodes for a graphene membrane of nanopore radii 0.26 (first column), 0.28 (second column), and 0.32 (third column) nm for the simulation times 0-10 (first row), 20-24 (second row), and 32-40 (third row) ns . The gray shaded region represents the anode, the red shaded region the cathode, and the dotted vertical line the position of the graphene membrane

MSD

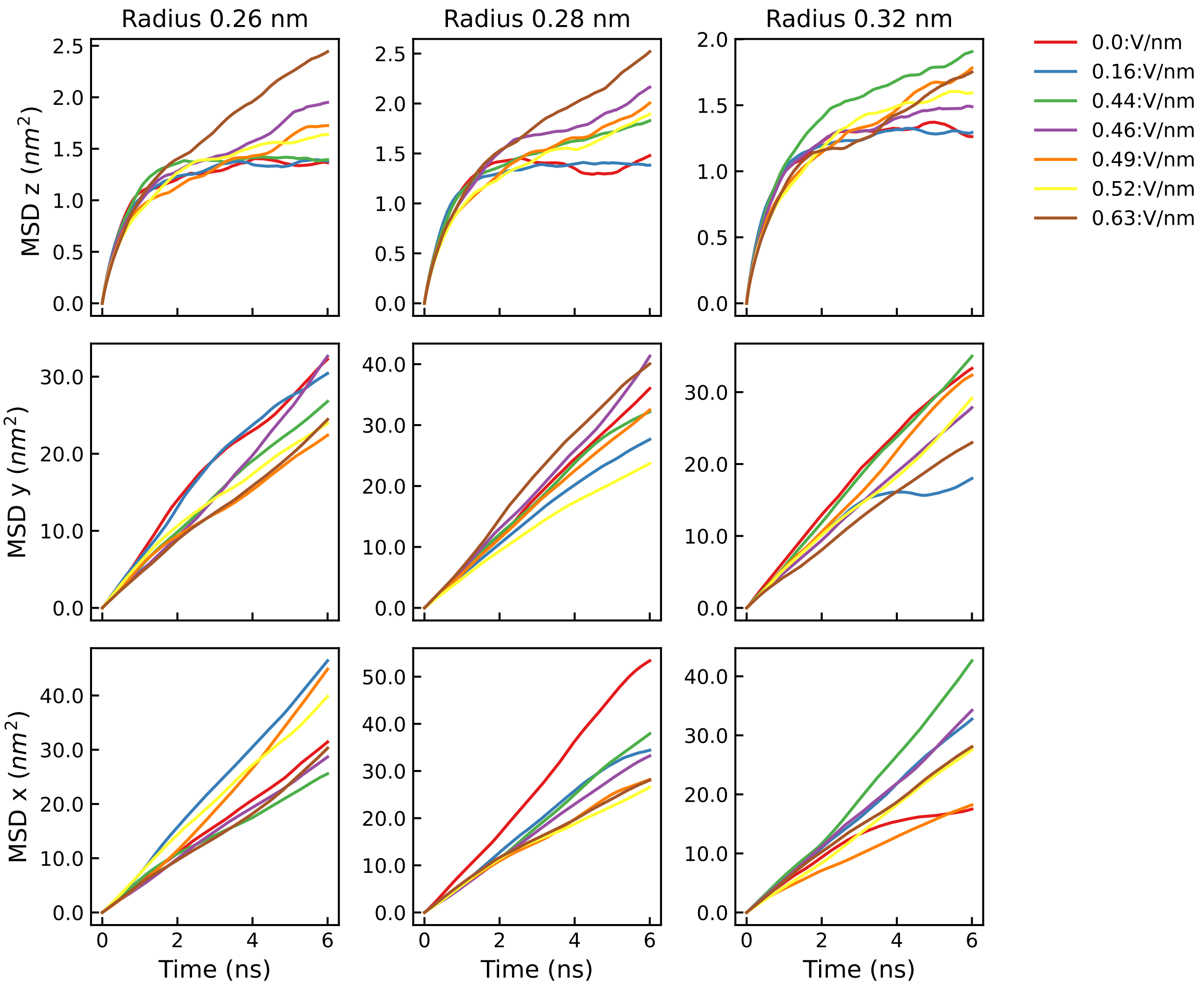

Figure S12: K^+ mean square displacement (MSD) components in the z (MSD_z top panel), y (MSD_y middle panel), and x (MSD_x bottom panel) axis across graphene membranes of nanopore radii of 0.26 (first column), 0.28 (second column), and 0.32 (third column) nm for K^+, Na^+, Cl^-, and Oxygen atom in water molecules under electric fields.

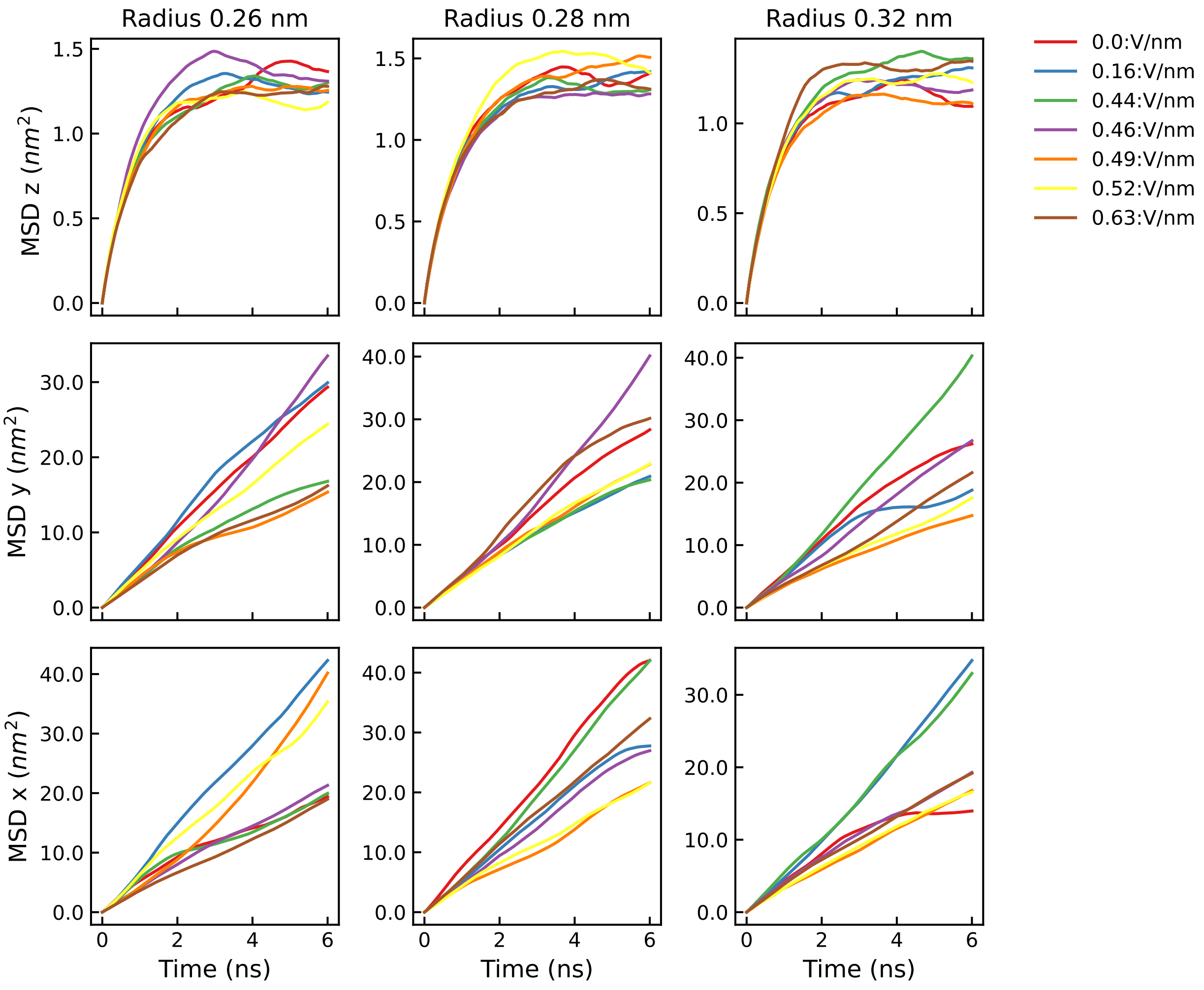

Figure S13: Na^+ mean square displacement (MSD) components in the z (MSD_z top panel), y (MSD_y middle panel), and x (MSD_x bottom panel) axis across graphene membranes of nanopore radii of 0.26 (first column), 0.28 (second column), and 0.32 (third column) nm for K^+, Na^+, Cl^-, and Oxygen atom in water molecules under electric fields.

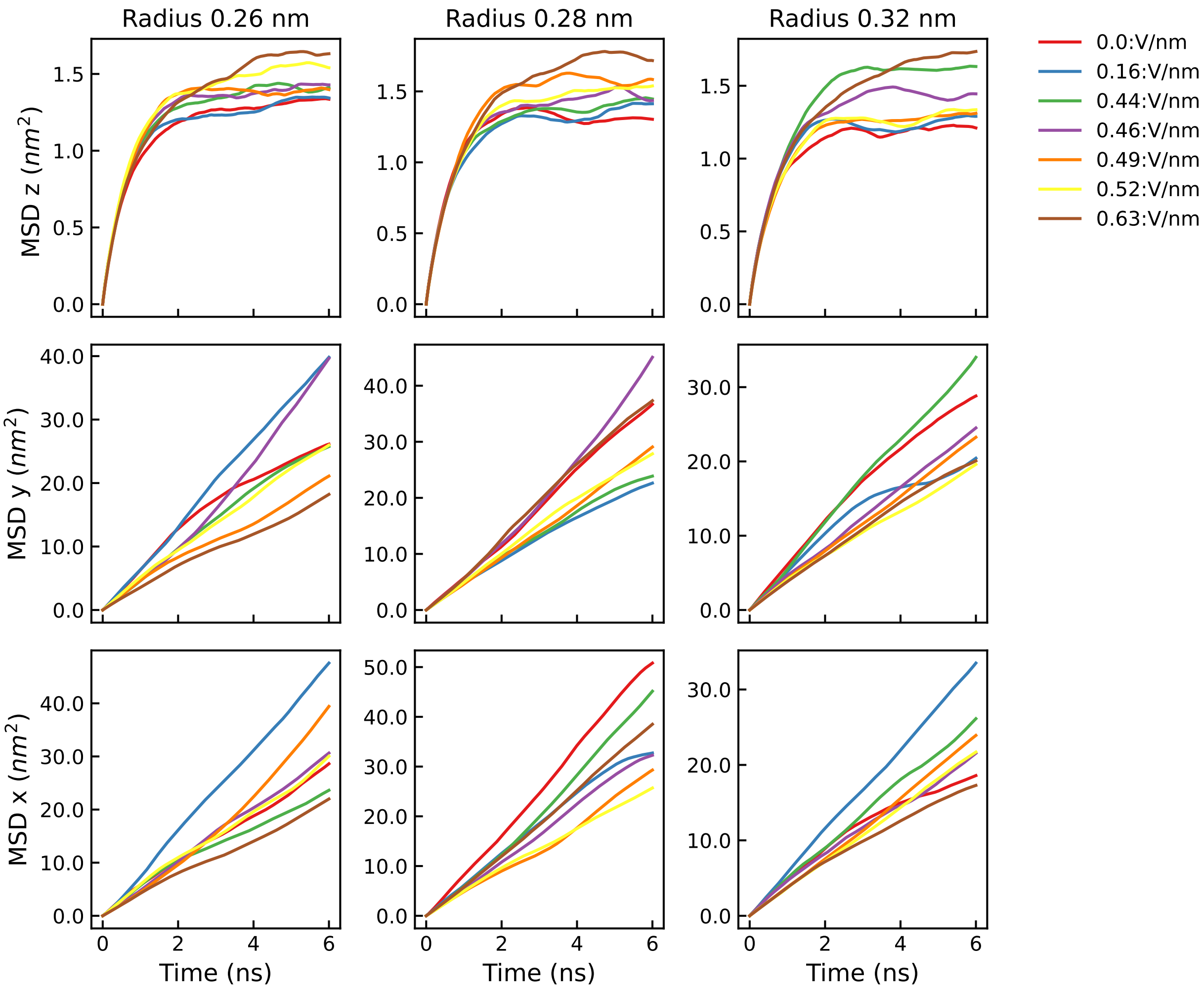

Figure S14: Cl^- mean square displacement (MSD) components in the z (MSD_z top panel), y (MSD_y middle panel), and x (MSD_x bottom panel) axis across graphene membranes of nanopore radii of 0.26 (first column), 0.28 (second column), and 0.32 (third column) nm for K^+, Na^+, Cl^-, and Oxygen atom in water molecules under electric fields.

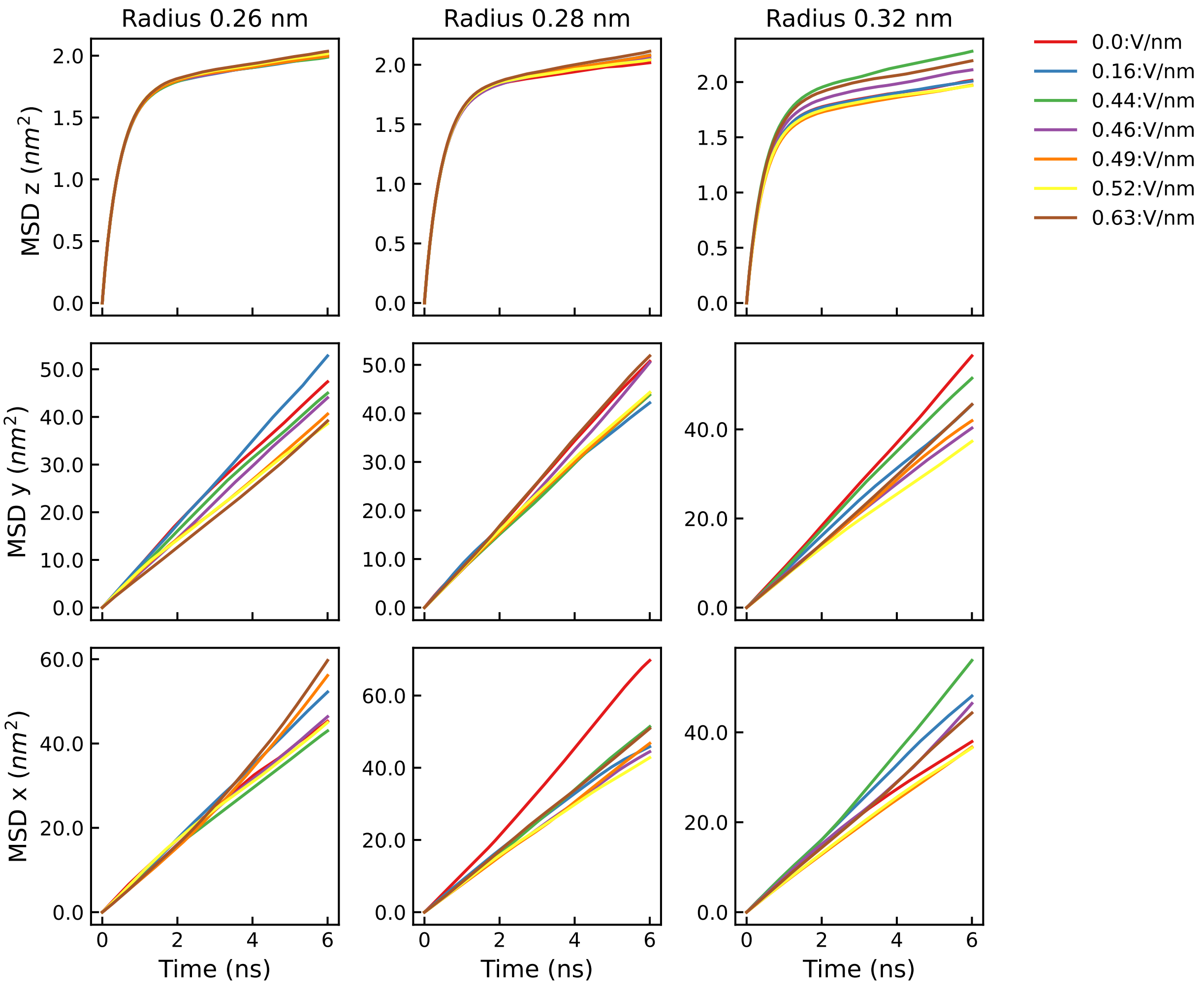

Figure S15: Oxygen in water mean square displacement (MSD) components in the z (MSD_z top panel), y (MSD_y middle panel), and x (MSD_x bottom panel) axis across graphene membranes of nanopore radii of 0.26 (first column), 0.28 (second column), and 0.32 (third column) nm for K^+, Na^+, Cl^-, and Oxygen atom in water molecules under electric fields.

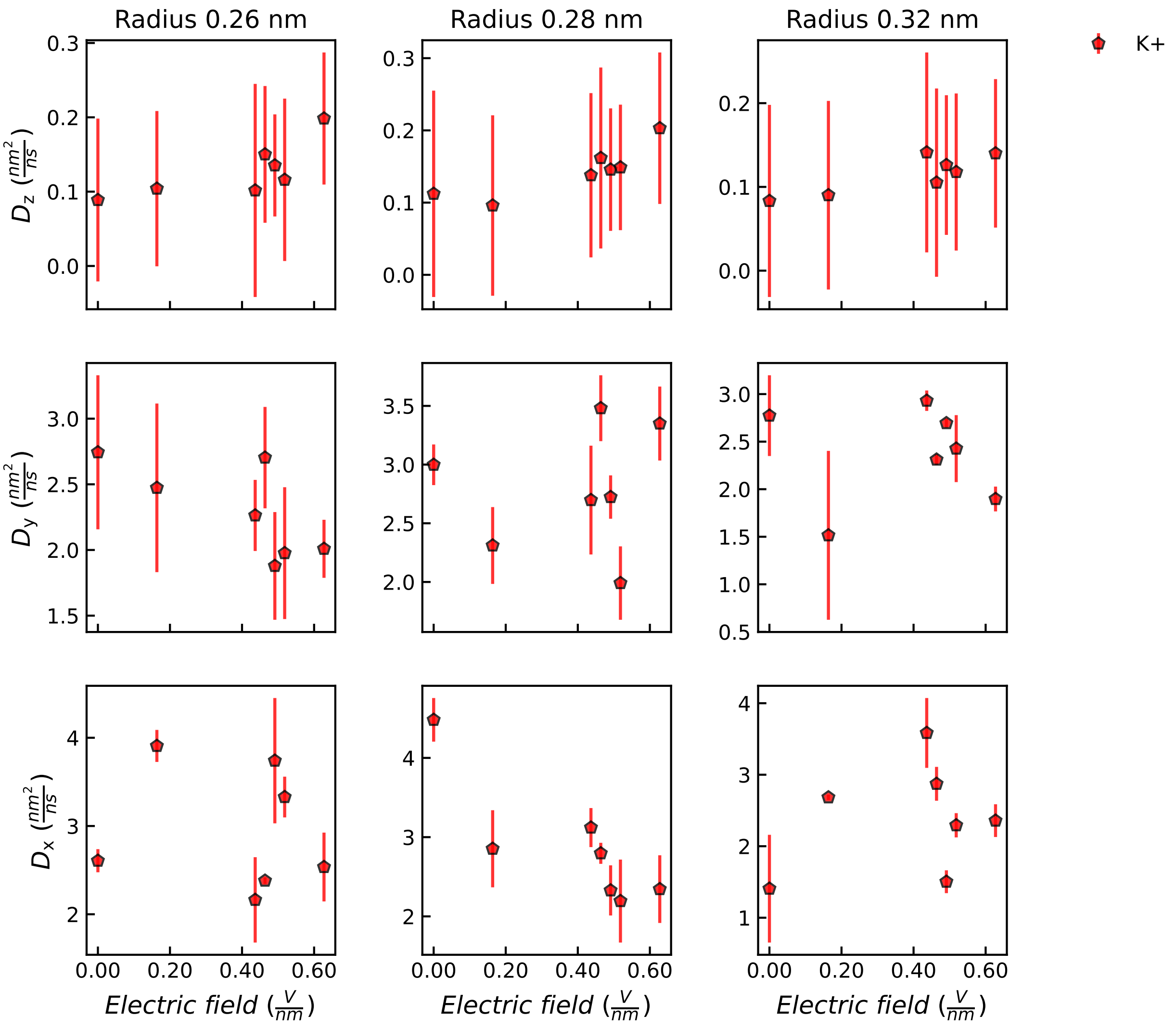

Figure S16: K^+ self-diffusion coefficient (D) components in the z (D_z top panel), y (D_y middle panel), and x (D_x bottom panel) axis as a function of the electric field intensity across graphene membranes of nanopore radii of 0.26 (first column), 0.28 (second column), and 0.32 (third column) nm for K^+, Na^+, Cl^-, and Oxygen atom in water molecules under electric fields.

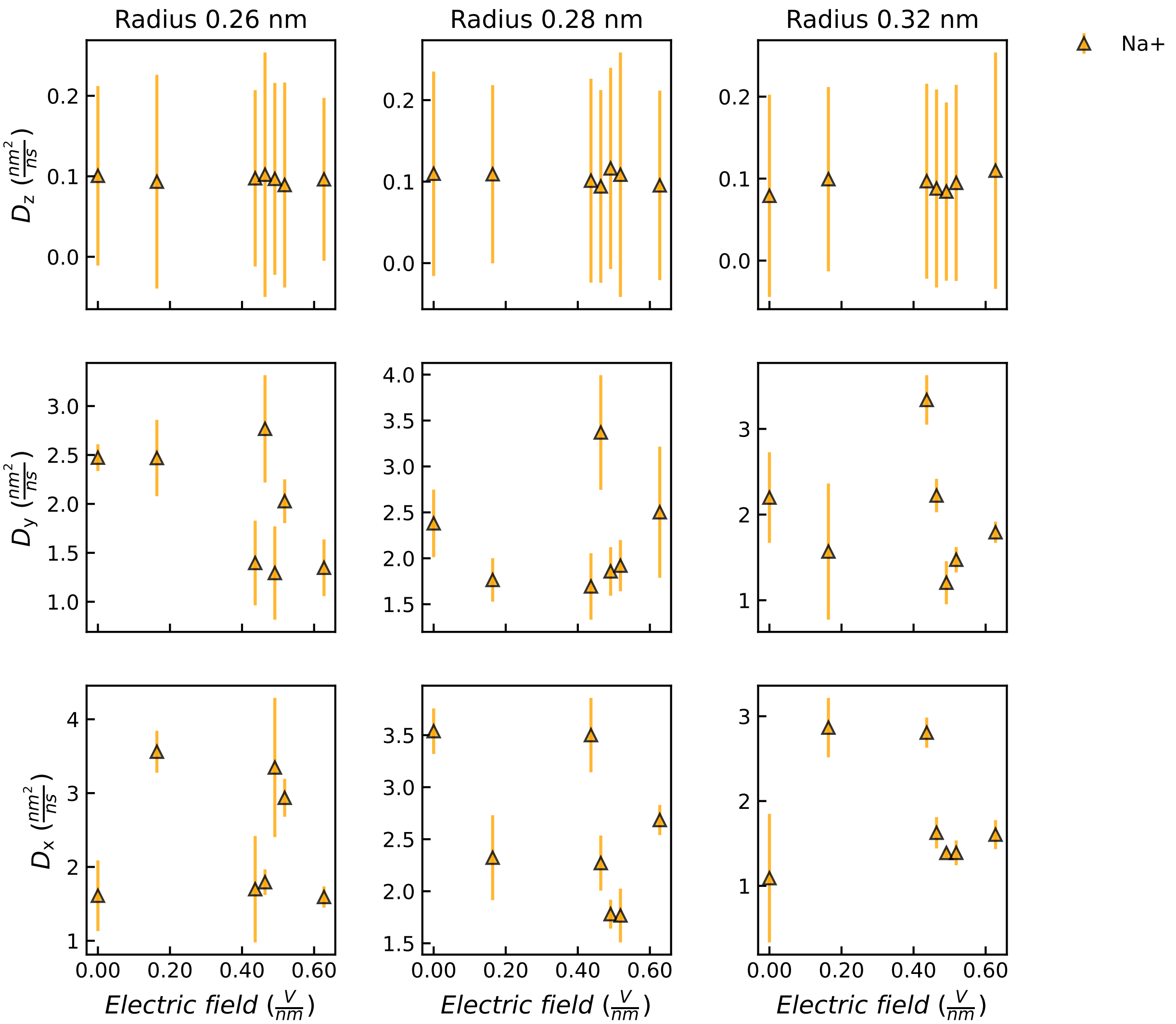

Figure S17: Na^+self-diffusion coefficient (D) components in the z (D_z top panel), y (D_y middle panel), and x (D_x bottom panel) axis as a function of the electric field intensity across graphene membranes of nanopore radii of 0.26 (first column), 0.28 (second column), and 0.32 (third column) nm for K^+, Na^+, Cl^-, and Oxygen atom in water molecules under electric fields.

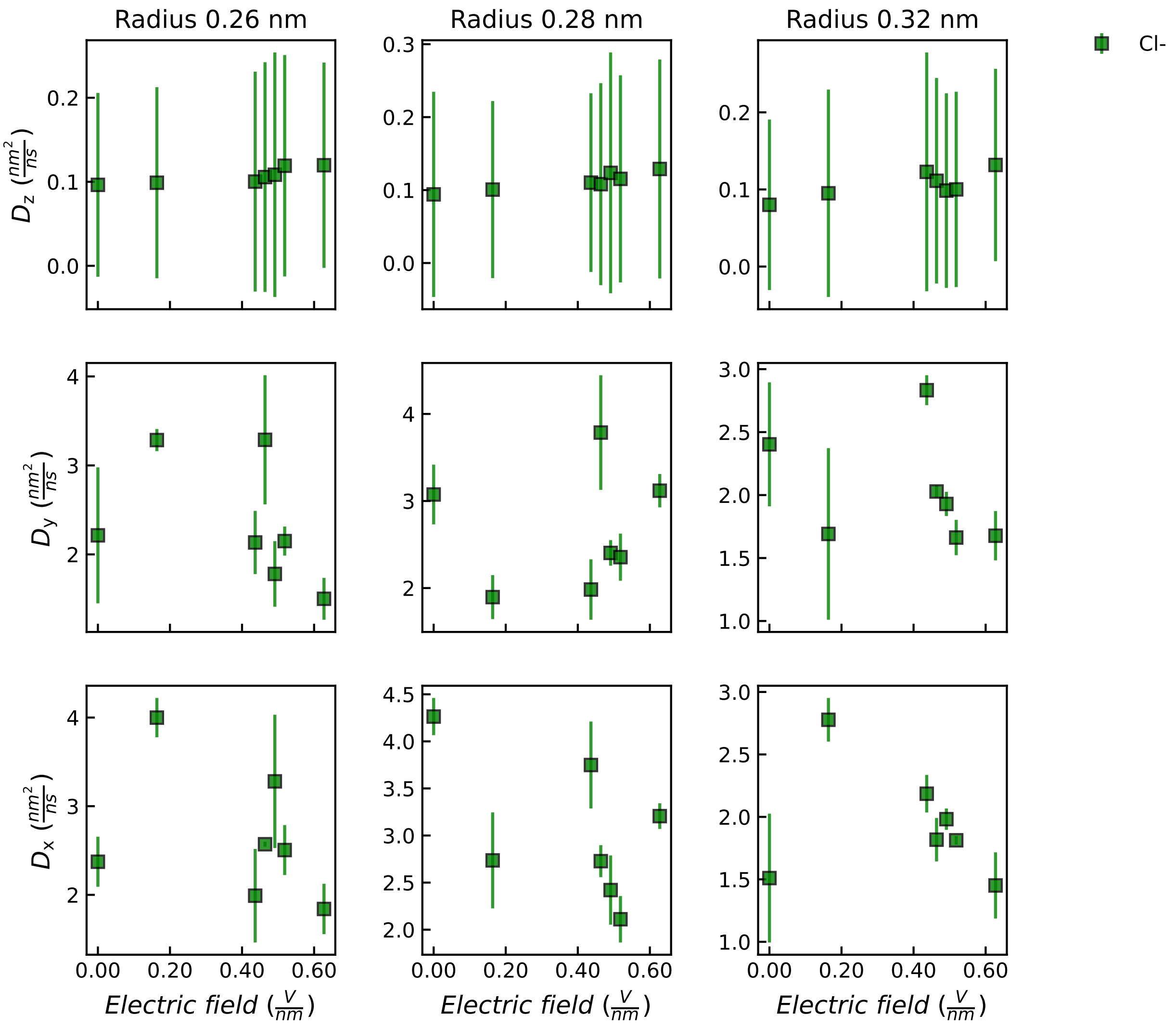

Figure S18: Cl^- self-diffusion coefficient (D) components in the z (D_z top panel), y (D_y middle panel), and x (D_x bottom panel) axis as a function of the electric field intensity across graphene membranes of nanopore radii of 0.26 (first column), 0.28 (second column), and 0.32 (third column) nm for K^+, Na^+, Cl^-, and Oxygen atom in water molecules under electric fields.

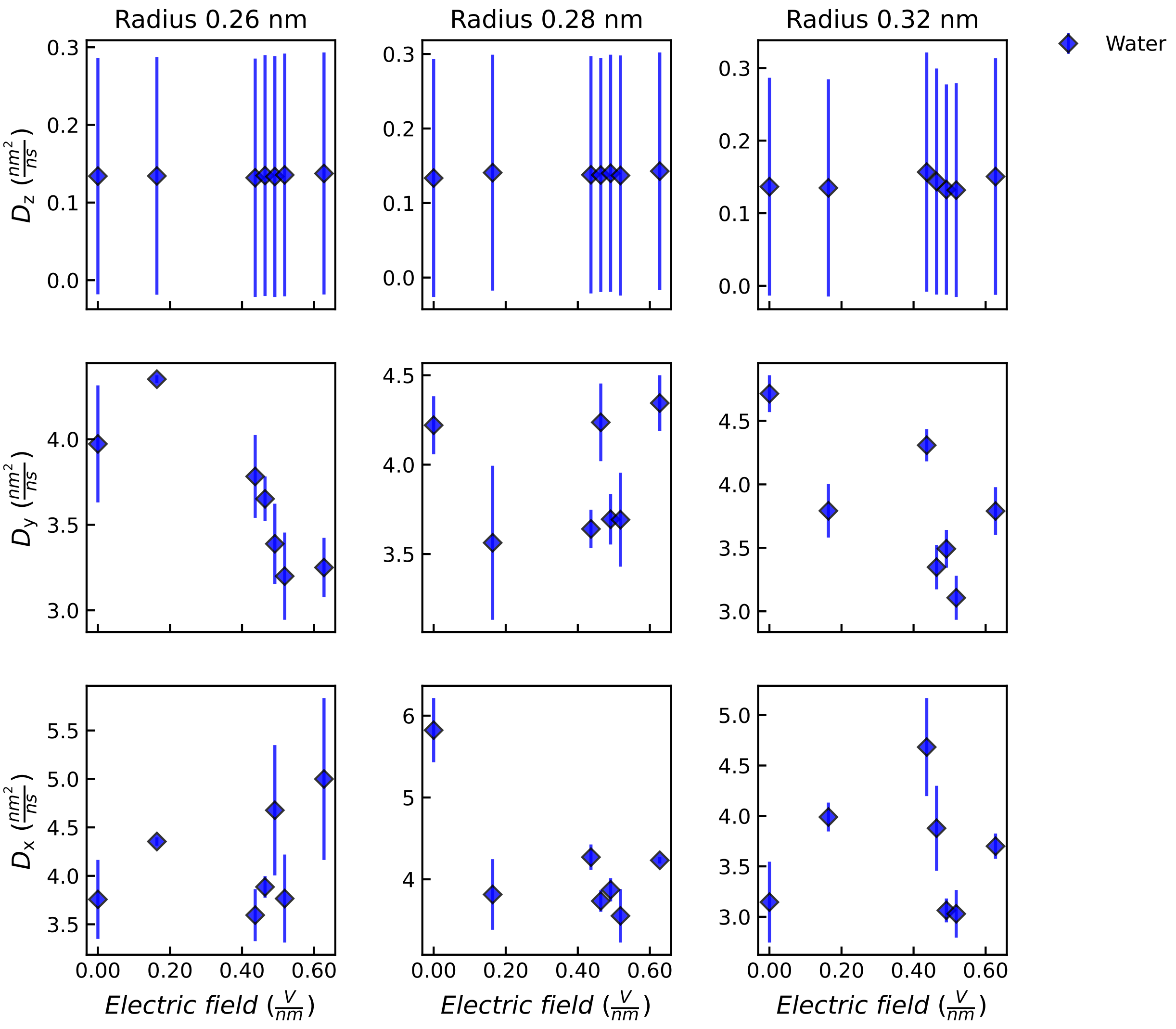

Figure S19: Oxygen in water self-diffusion coefficient (D) components in the z (D_z top panel), y (D_y middle panel), and x (D_x bottom panel) axis as a function of the electric field intensity across graphene membranes of nanopore radii of 0.26 (first column), 0.28 (second column), and 0.32 (third column) nm for K^+, Na^+, Cl^-, and Oxygen atom in water molecules under electric fields.

Electrical mobility

The terminal drift velocity distribution along the z-direction was obtained from the instantaneous velocities of individual ions, calculated between consecutive simulation frames. This approach was necessary to account for confinement effects, as ion movement is restricted and passage limited by the graphene membrane—an effect also evident in the mean squared

displacement along the z-axis. Traditionally, the terminal velocity along the z-direction is computed from the slope of the displacement versus time in the direction of the applied electric field, which follows a linear trend in unconfined systems.[10,11] The instantaneous drift velocity distribution in the direction in the direction of the electric field is shown in Fig. S20

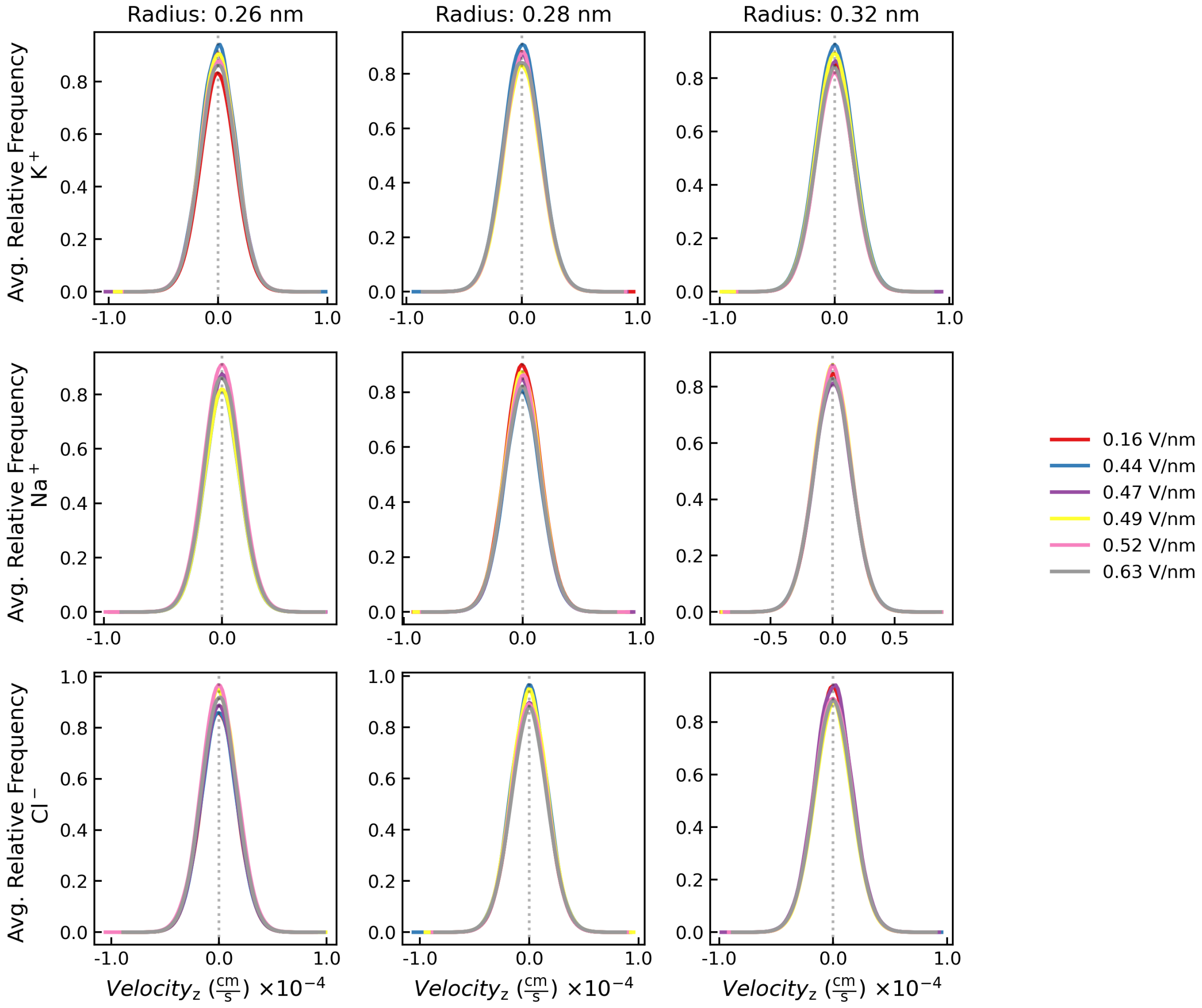

Figure S20: Terminal drift velocity distribution, V_z under external fields generated *via* different potential differences for K^+ (first row), Na^+ (second row), Cl^- (third row) across graphene membranes of nanopore radii 0.26 (first column), 0.28 (second column), and 0.32 (third column) nm.

With the terminal drift velocity across the z-direction, V_z, the electrical mobility of each ion, μ_q, was computed as shown in Fig. ll Using the terminal drift velocity in the z-direction, V_z, the electrical mobility of each ion, μ_q, was computed, as shown in Fig. S21.

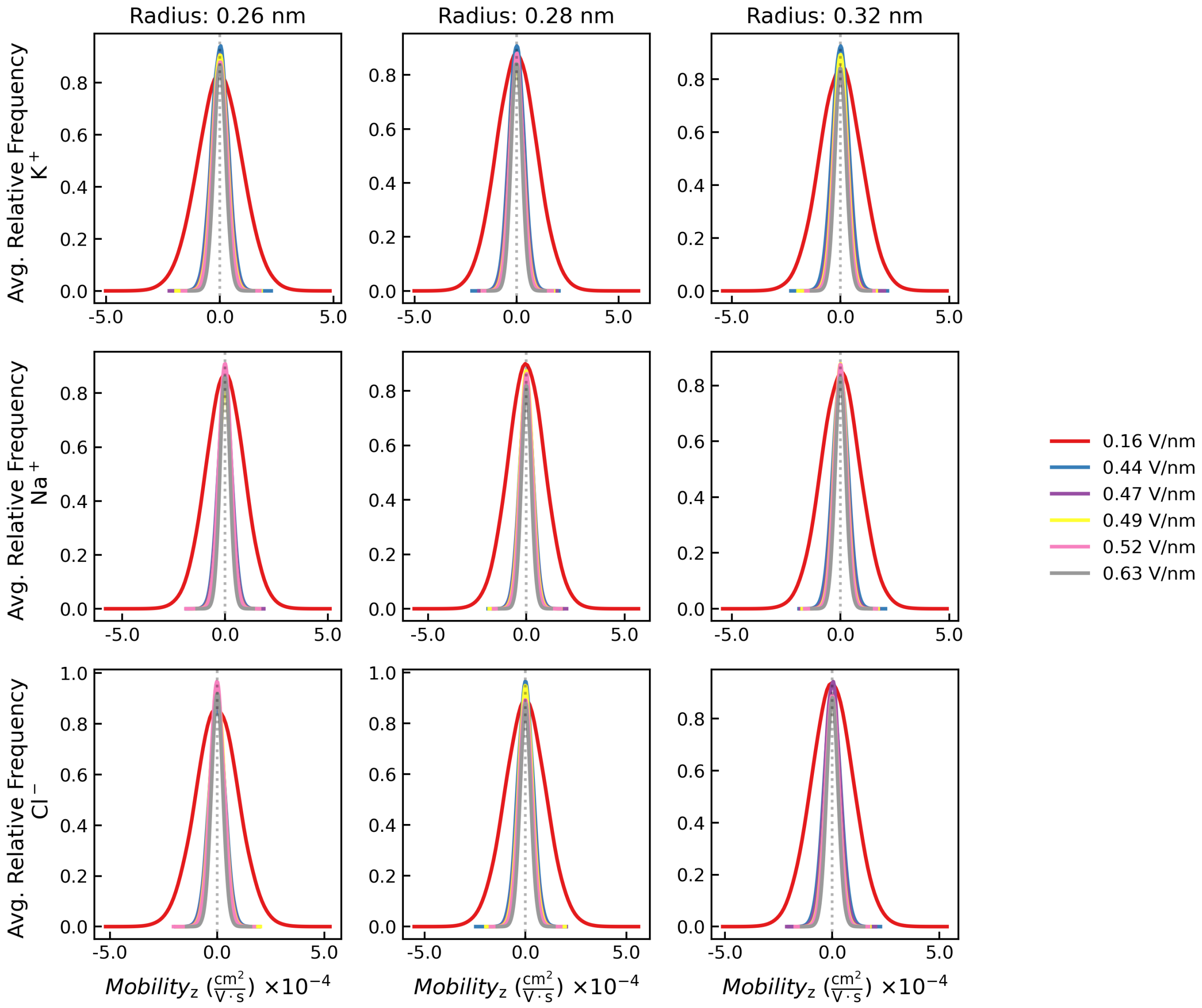

Figure S21: Electrical mobility distribution,μ_q, under external fields generated *via* different potential differences for K^+ (first row), Na^+ (second row), Cl^- (third row) across graphene membranes of nanopore radii 0.26 (first column), 0.28 (second column), and 0.32 (third column) nm.

Water structuring

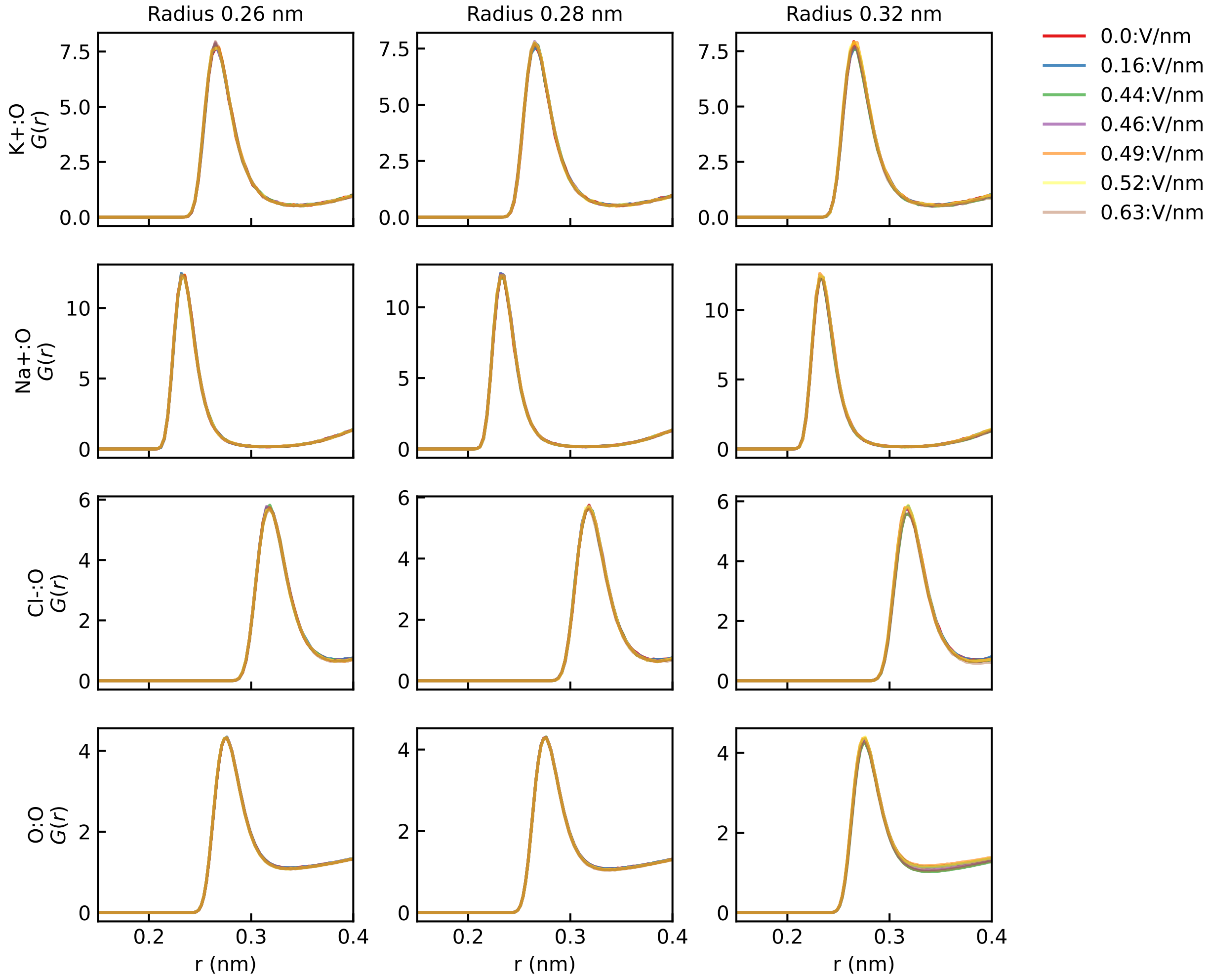

Figure S22: Ion-oxygen RDFs $g_{ij}(r)$ under external fields for K^+ (first row), Na^+ (second row), Cl^- (third row) -, and oxygen in water (bottom row) across graphene membranes of nanopore radii 0.26 (first column), 0.28 (second column), and 0.32 (third column) nm.

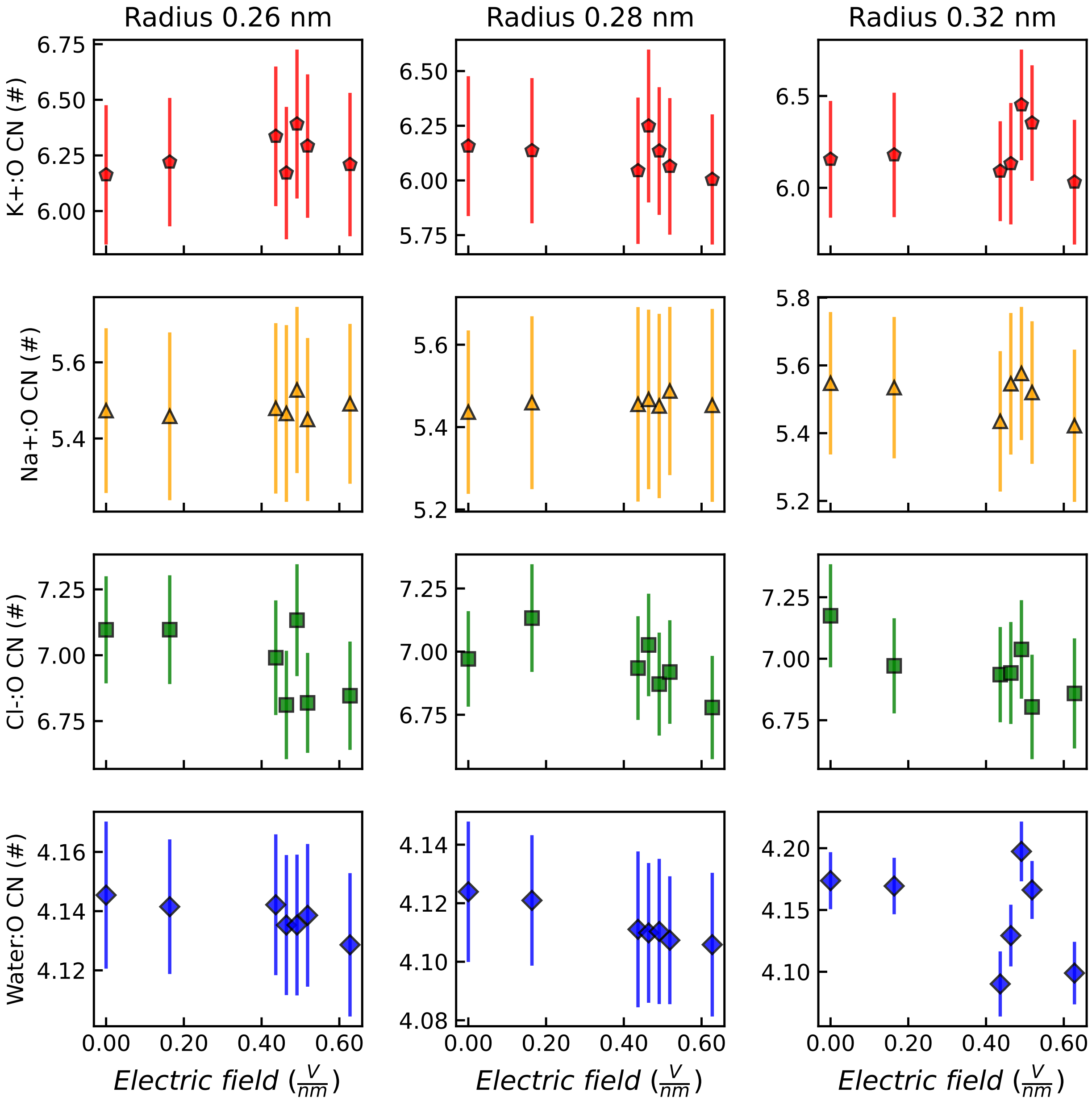

Figure S23: Coordination number in the FHS as a function of the electric field intensity for K^+ (first row), Na^+ (second row), Cl^- (third row) -, and oxygen in water (bottom row) across graphene membranes of nanopore radii 0.26 (first column), 0.28 (second column), and 0.32 (third column) nm.

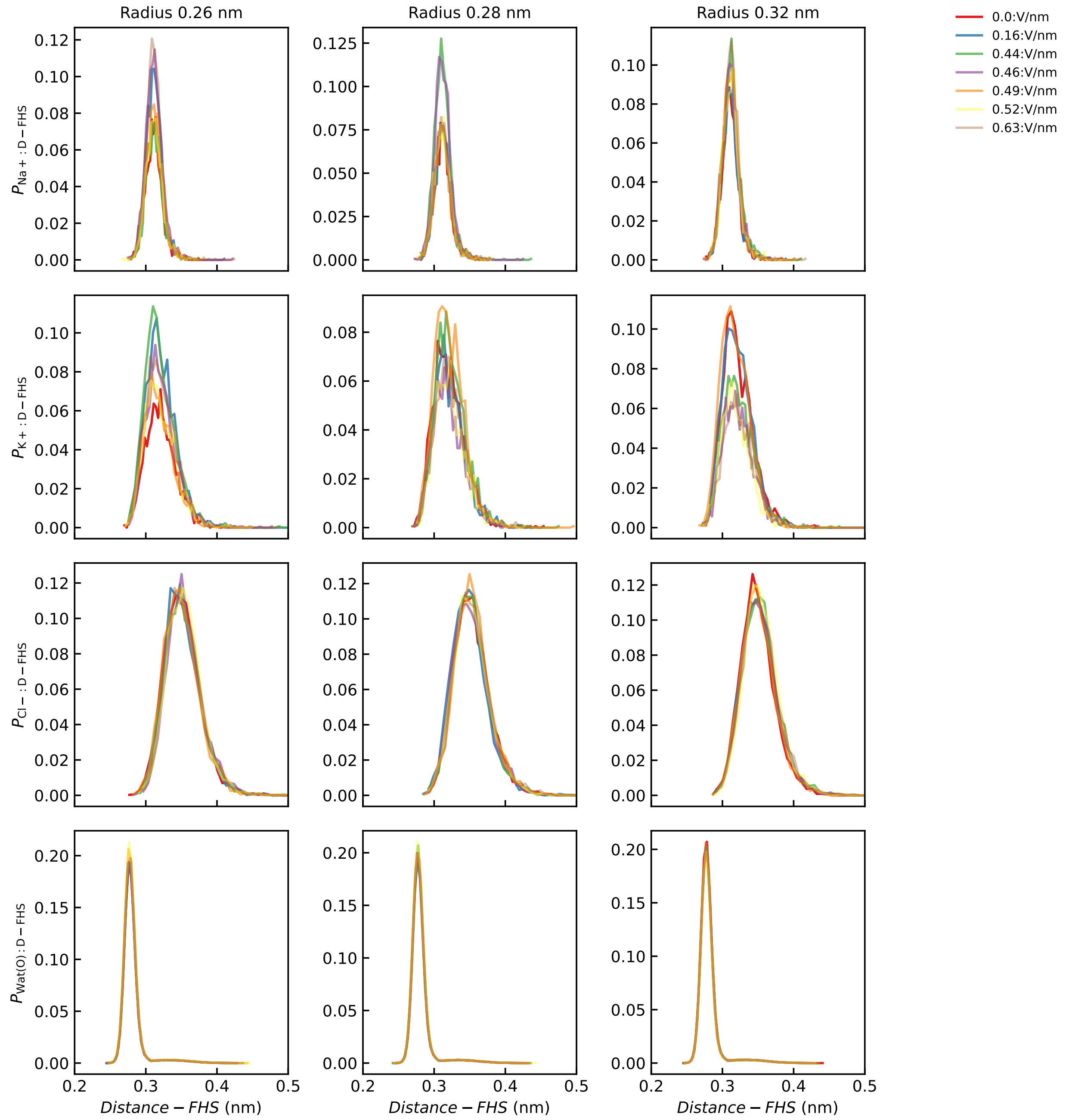

Figure S24: Distance probability between ions or oxygen in water molecules and the oxygen in other water molecules within the FHS as a function under electric fields for Na^+ (first row), K^+ (second row), Cl^- (third row) -, and oxygen in water (bottom row) across graphene membranes of nanopore radii 0.26 (first column), 0.28 (second column), and 0.32 (third column) nm.

To characterize water orientation, the dipole vector of each molecule as the vector from the oxygen atom to the midpoint between its two hydrogen atoms. This dipole vector was then

compared to the applied electric field direction, defined along the z-axis, to obtain the angle Φ between the two. For spatially resolved analysis, molecules were classified based on the z-coordinate of their center of mass relative to the simulation box: molecules within 0.5 nm of the lower boundary were assigned to the cathode region, those within 0.5 nm of the upper boundary to the anode region, and those within ± 0.5 nm of the membrane midplane to the membrane region. Molecules outside these regions were treated as bulk. Orientation distributions were constructed by binning the angles. The degree distributions spanned the range [0°-180°]. Three resolutions were considered: increments of 2°, 10°and 20°.

bin size = 2 degrees

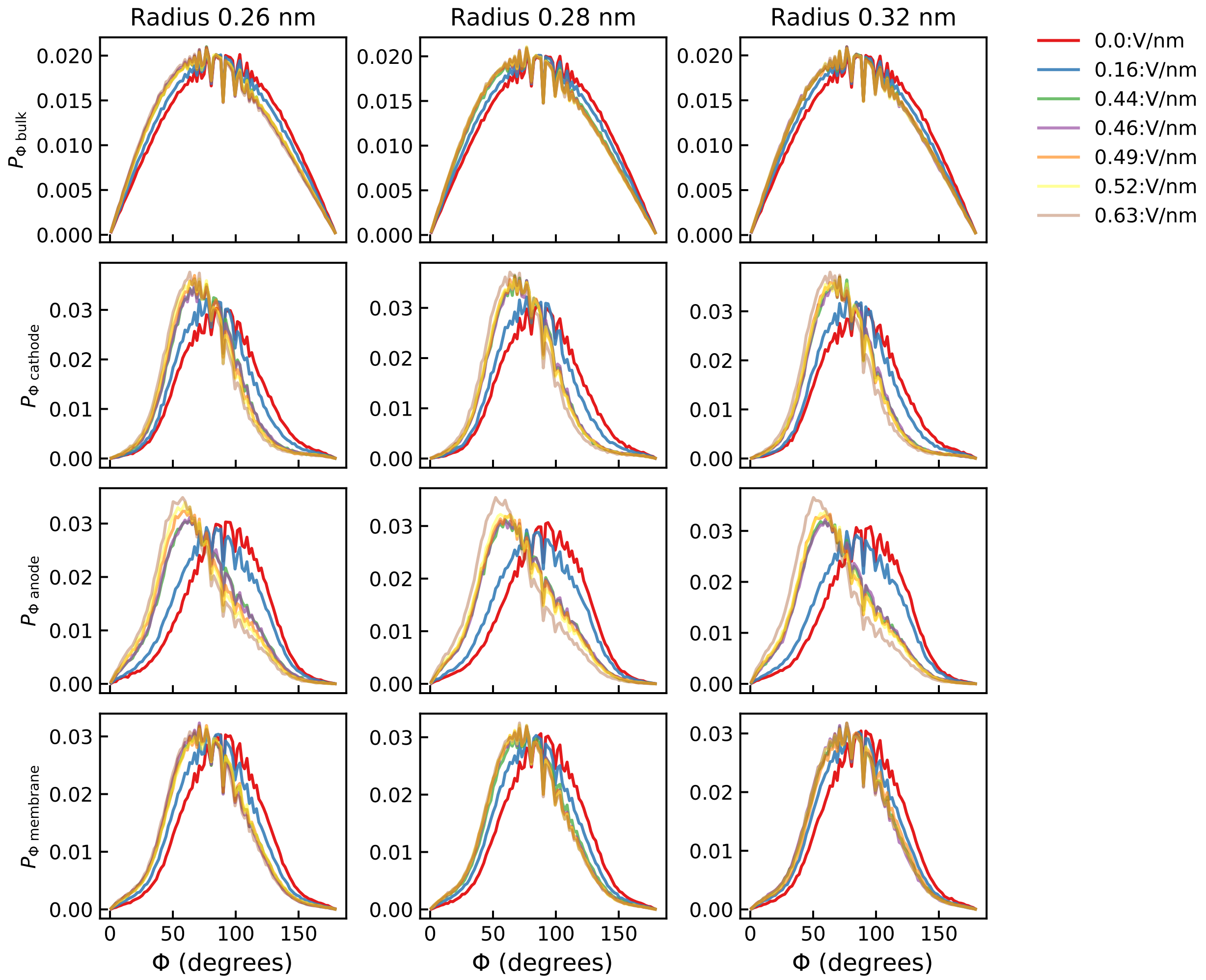

Figure S25: Probability distribution of the angle Φ between the water molecule dipole and the electric field vector (0,0,1) in four regions: the bulk of the simulation box (Φ_{bulk}, first row), within 0.5 nm of the cathode (Φ_{cathode}, second row), within 0.5 nm of the anode (Φ_{anode}, third row), and within 0.5 nm of the graphene membrane (Φ_{membrane}, last row). Results are shown under applied electric fields across graphene membranes with nanopore radii of 0.26 nm (first column), 0.28 nm (second column), and 0.32 nm (third column). Bin size: 2°.

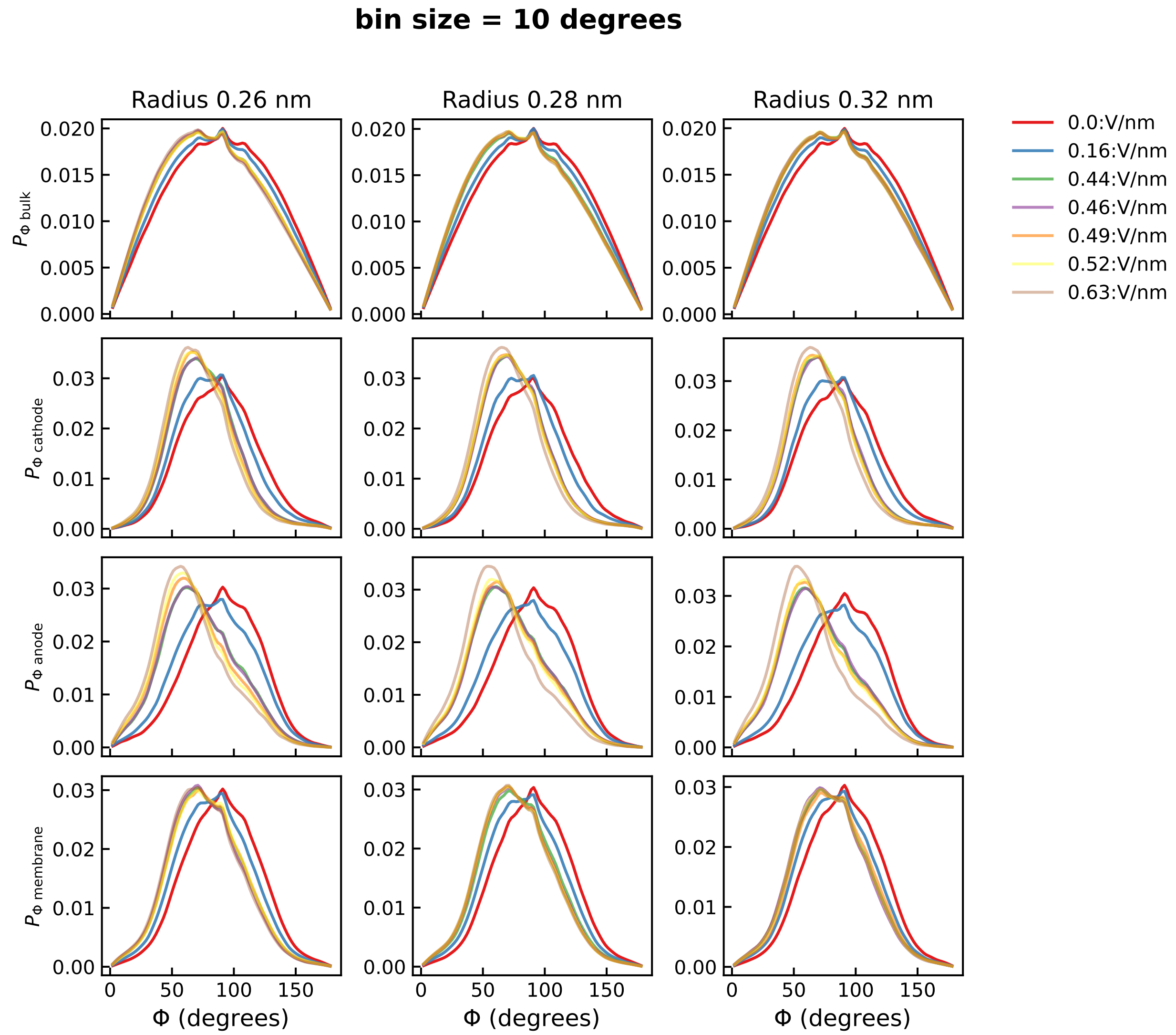

Figure S26: Probability distribution of the angle Φ between the water molecule dipole and the electric field vector (0,0,1) in four regions: the bulk of the simulation box (Φ_{bulk}, first row), within 0.5 nm of the cathode (Φ_{cathode}, second row), within 0.5 nm of the anode (Φ_{anode}, third row), and within 0.5 nm of the graphene membrane (Φ_{membrane}, last row). Results are shown under applied electric fields across graphene membranes with nanopore radii of 0.26 nm (first column), 0.28 nm (second column), and 0.32 nm (third column). Bin size: 10°.

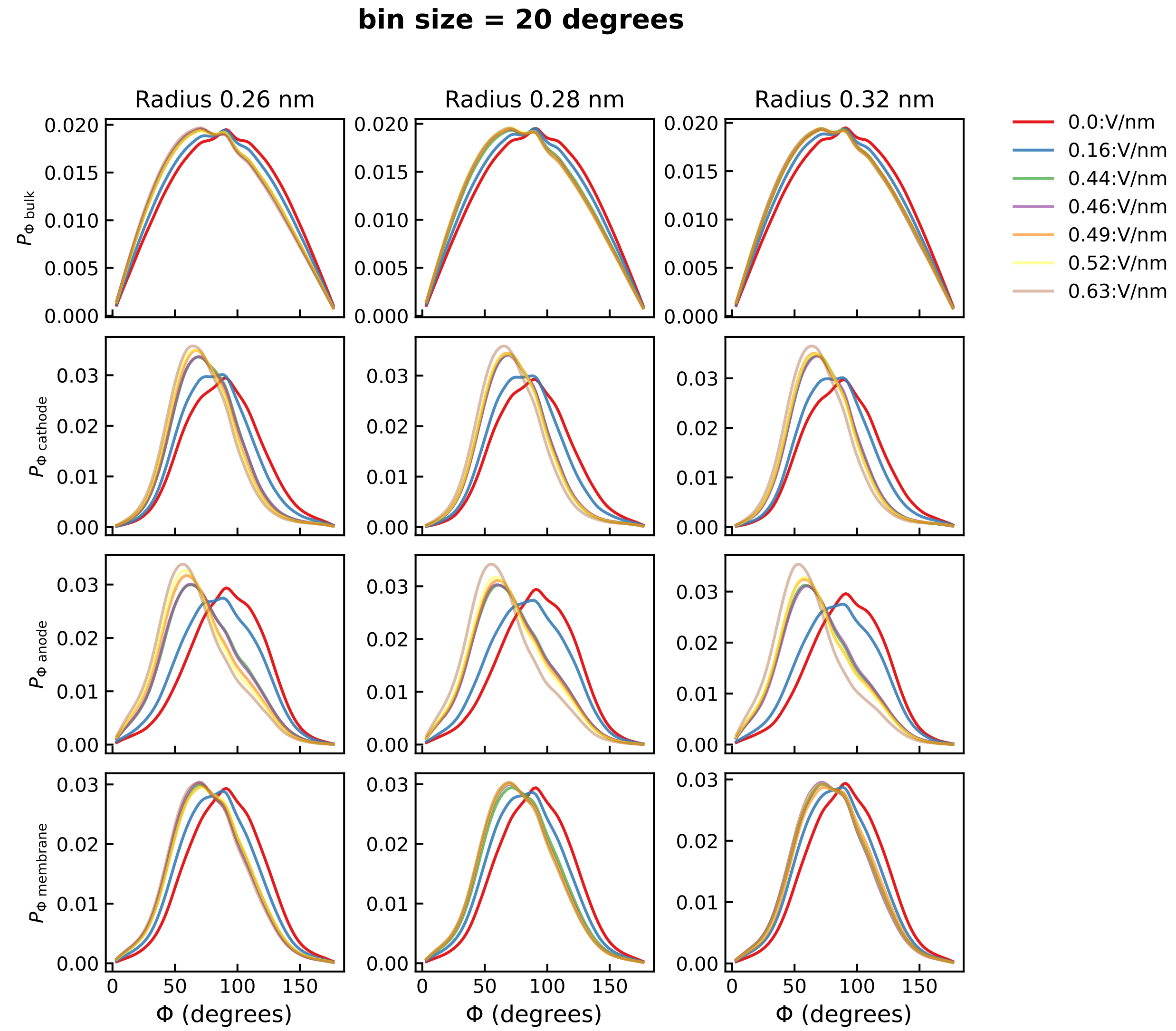

Figure S27: Probability distribution of the angle Φ between the water molecule dipole and the electric field vector (0,0,1) in four regions: the bulk of the simulation box (Φ_{bulk}, first row), within 0.5 nm of the cathode (Φ_{cathode}, second row), within 0.5 nm of the anode (Φ_{anode}, third row), and within 0.5 nm of the graphene membrane (Φ_{membrane}, last row). Results are shown under applied electric fields across graphene membranes with nanopore radii of 0.26 nm (first column), 0.28 nm (second column), and 0.32 nm (third column). Bin size: 20°.

Furthermore, the mean and standard deviation of the Φ angle distributions were computed for the cases with electric field strengths of 0.0 V/nm and 0.63 V/nm. This analysis highlights the negligible effects of binning while capturing the orientation changes induced by the applied electric field. In addition, the orientation angle Φ was evaluated based on the proximity of water molecules to ions in solution (Na^+, K^+, and Cl^-), distinguishing between

water molecules located more than 0.4 nm away from any ion and those within 0.4 nm of an ion. The Φ angle distributions of water molecules located near any of the ions (within 0.4 nm) and those farther than 0.4 nm from all ions were normalized as conditional distributions. The overall angular distribution is a weighted mixture of the two:

$$p_(\phi) = w_{\text{near}}\, p(\phi \mid \text{near}) + w_{\text{away}}\, p(\phi \mid \text{away}),$$

where $w_{\text{near}} = \frac{N_{\text{near}}}{N_{\text{near}}+N_{\text{away}}}$ and $w_{\text{away}} = 1 - w_{\text{near}}$, with N_{near} and N_{away} denoting the number of water molecules in each category.

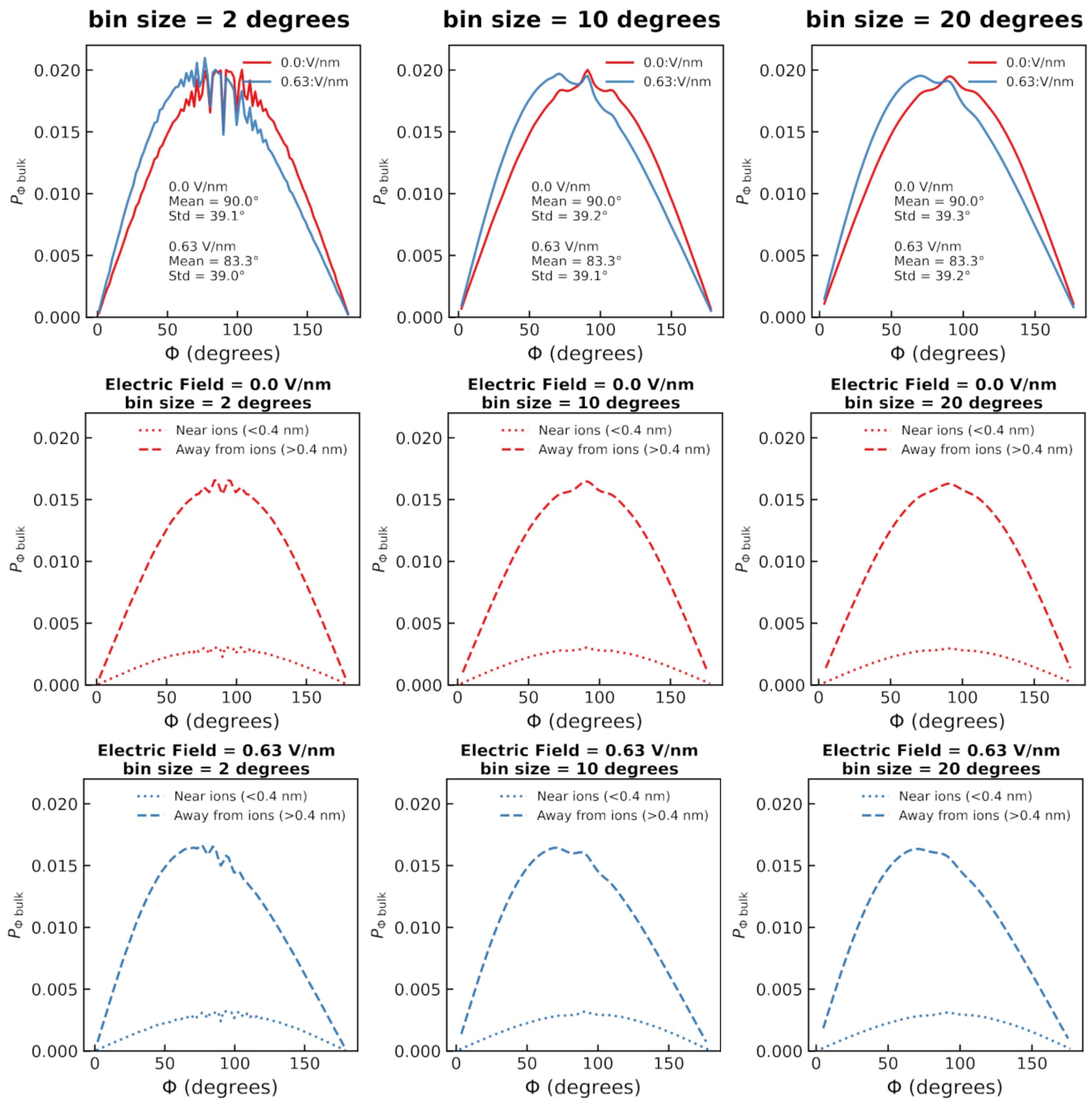

Figure S28: Probability distribution of the angle Φ between the water molecule dipole and the electric field vector (0,0,1) in the bulk of the simulation box (Φ_{bulk}). Top row: results for electric fields of 0.0 V/nm and 0.63 V/nm, including the mean and standard deviation of each distribution. Middle and bottom rows: Φ_{bulk} distributions separated into water molecules near the ions (dotted line) and those more than 0.4 nm from any ion (dashed line), shown for 0.0 V/nm (middle row) and 0.63 V/nm (bottom row). Columns correspond to different bin sizes: 2°(first column), 10°(second column), and 20°(third column).

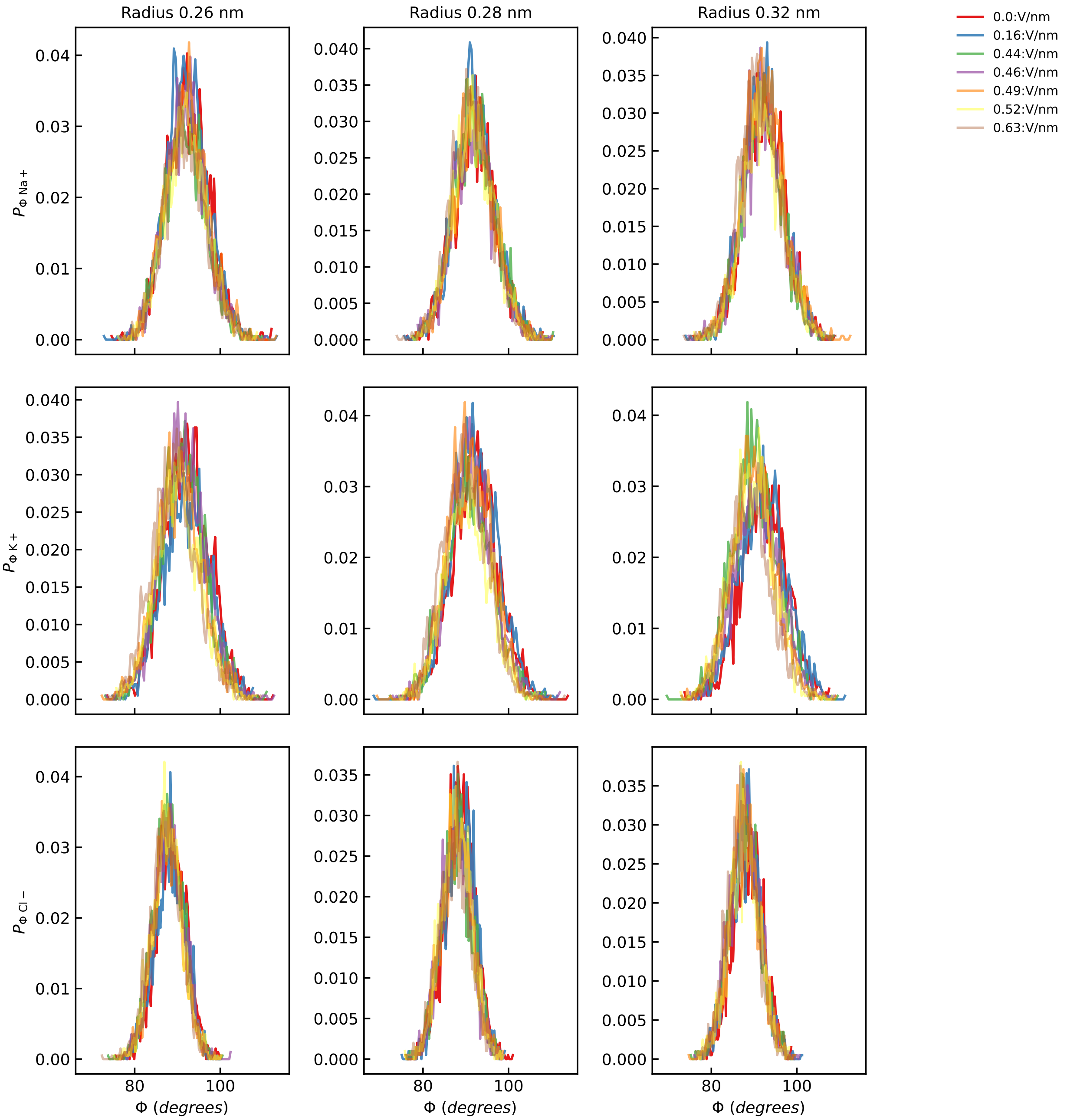

Figure S29: Probability distributions of the orientation angle between the water molecules dipole and the vector between the ion and the oxygen in the water molecule (Φ) of the water molecules in the FHS under electric fields across graphene membranes of nanopore radii 0.26 (first column), 0.28 (second column), and 0.32 (third column) nm for Na^+ (first row), K^+ (middle row), and Cl^- (bottom row).

The TCFs associated with isosceles triangle configurations with probability of finding the tetrahedral angle $\approx 109°$, denoted by $g_{\text{O-A-O}}(r, r, s)$ (where $r = r \neq s$) is plotted on the right

panel and the probability of finding 60 $\circ$ angle, denoted by $g_{\text{O-A-O}}(r, r, r)$ (where $r = r = r$) is plotted with the dashed line. Note that in the first case where the θ is the angle formed by the two sides of same length of a triplet and is related with third length as $s^2 = 2r^2(1-\cos\theta)$.

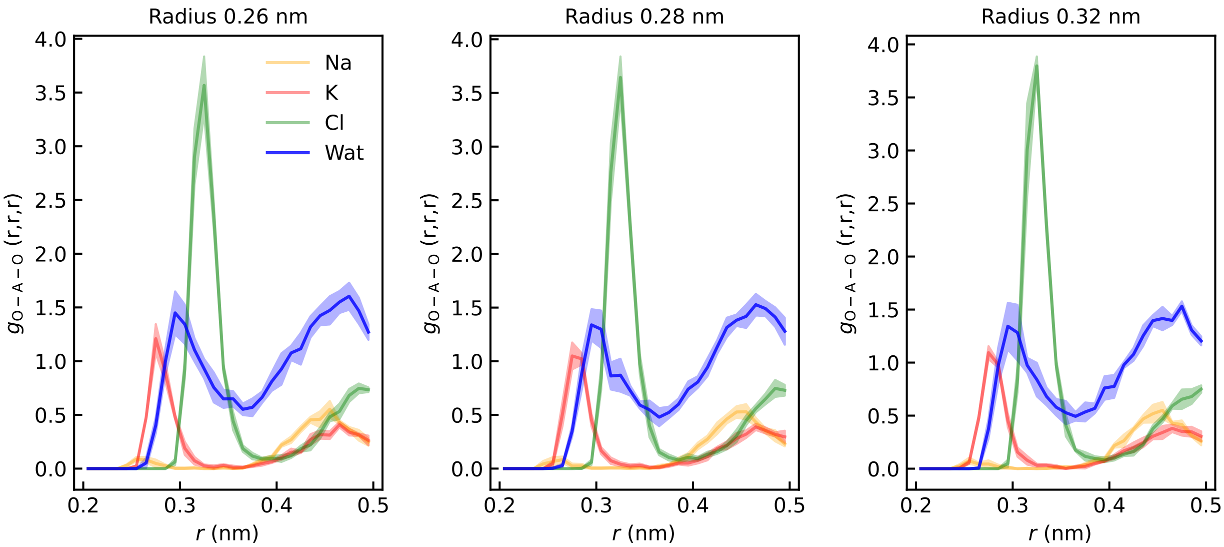

Figure S30: O-A-O *TCF*s, averaged across electric fields of magnitude 0.0, 0.46, 0.49, and 0.63 V/nm for graphene membranes with nanopore radii of 0.26 (left panel) 0.28 (middle panel), and 0.32 (right panel) nm, were A stands for Na^+ (orange), K^+ (red), Cl^- (green) and oxygen in water (blue). The *TCF*s associated with equilateral triangle configurations with probability of finding $\theta = 60^\circ$, denoted by$g_{\text{O-A-O}}(r, r, r)$ (where $r = r = r$).

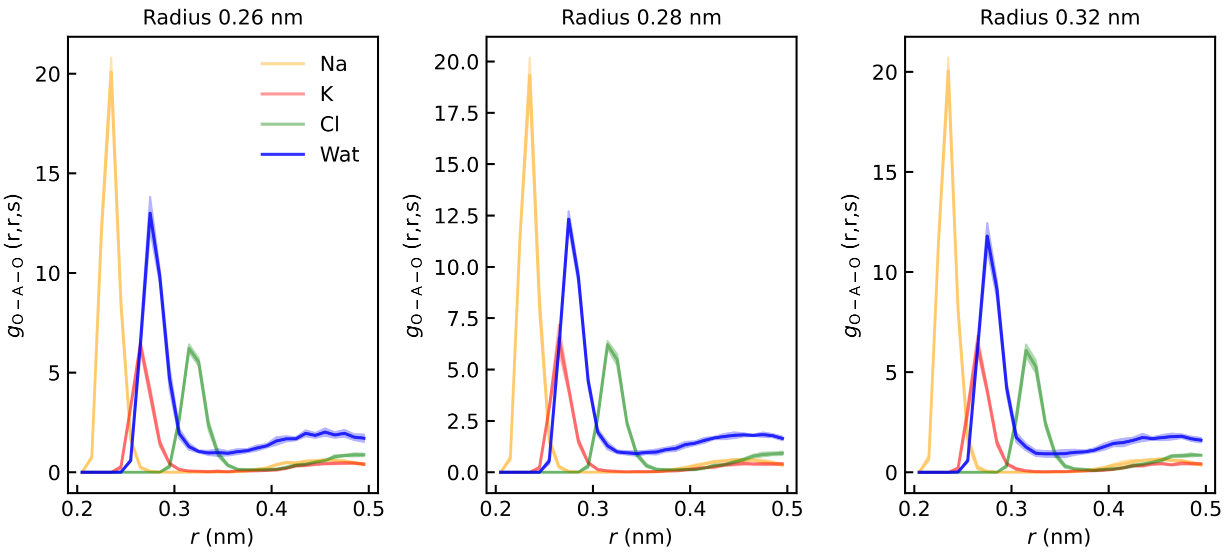

Figure S31: O-A-O *TCF*s, averaged across electric fields of magnitude 0.0, 0.46, 0.49, and 0.63 V/nm for graphene membranes with nanopore radii of 0.26 (left panel) 0.28 (middle panel), and 0.32 (right panel) nm, where A stands for Na^+ (orange), K^+ (red), Cl^- (green) and oxygen in water (blue). The *TCF*s associated with isosceles triangle configurations with probability of finding $\theta = 109°$, denoted by $g_{\text{O-A-O}}(r, r, s)$ (where $r = r \neq s$). Note that in this case where the θ angle is formed by the two sides of same length of a triplet and is related with third length as $s^2 = 2r^2(1 - \cos\theta)$.

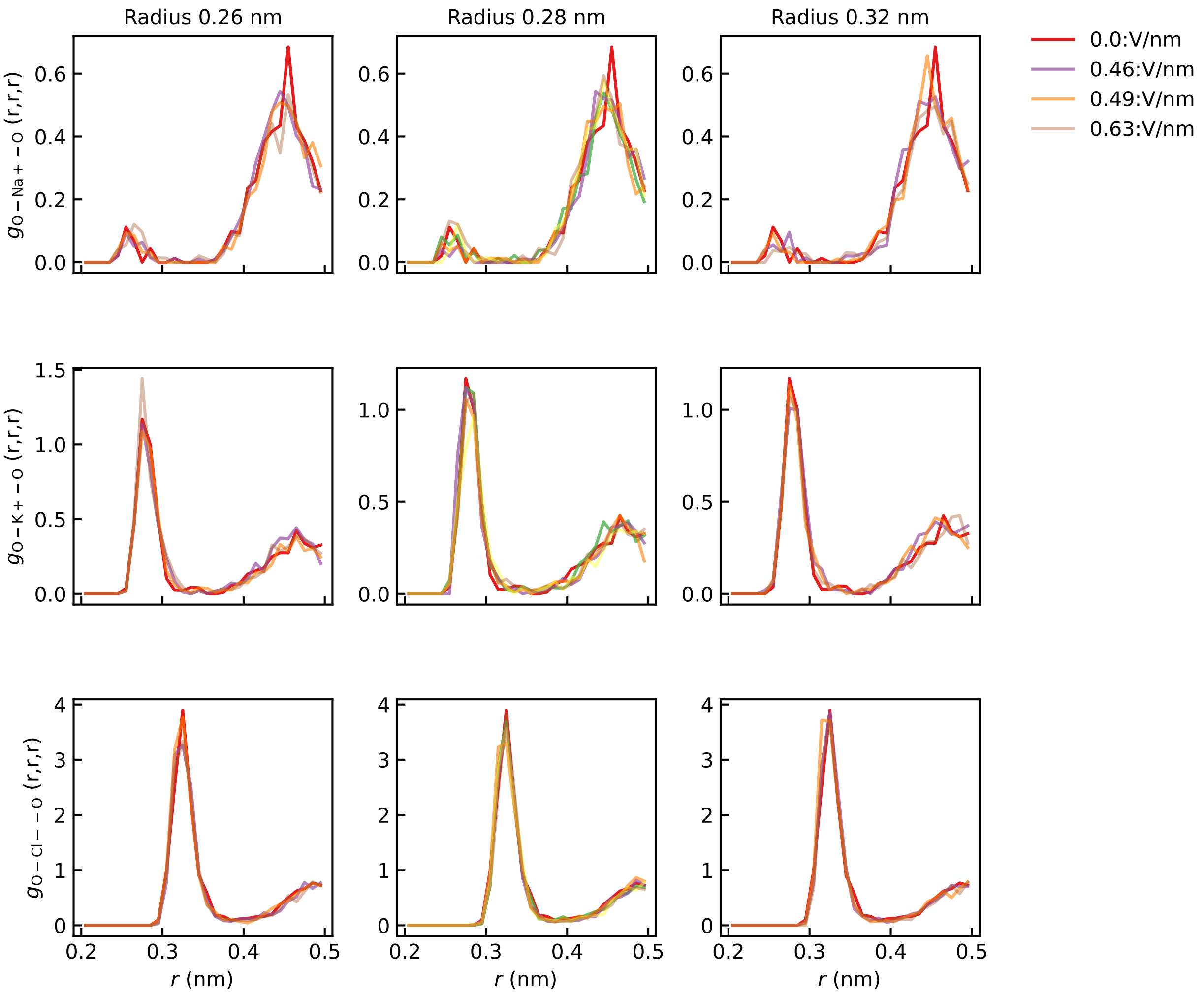

Figure S32: O-A-O *TCF*s across electric fields of magnitude 0.0, 0.46, 0.49, and 0.63 V/nm for graphene membranes with nanopore radii of 0.26 (first column) 0.28 (second column), and 0.32 (third column) nm, where A stands for Na^{+} (first row), K^{+} (middle row), and Cl^{-} (bottom row). The *TCF*s associated with equilateral triangle configurations with probability of finding $\theta = 60°$.

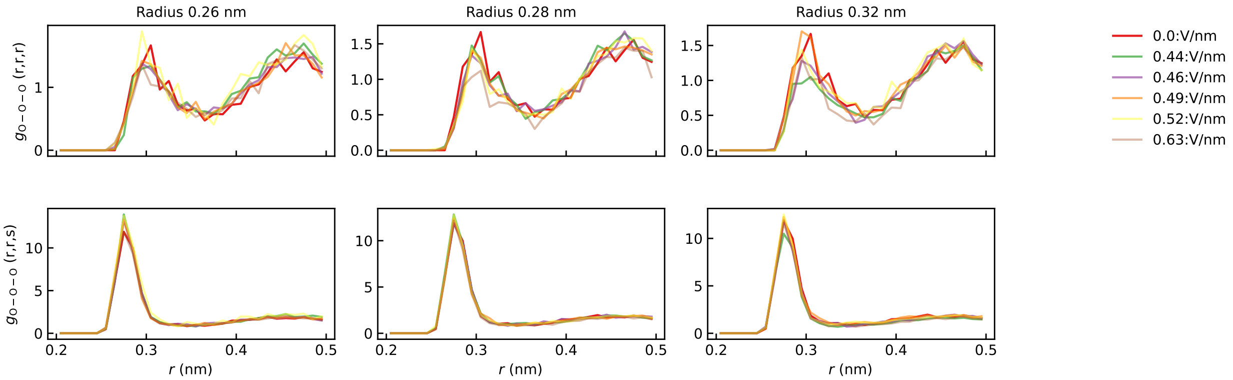

Figure S33: O-Oxygen in water-O TCFs across electric fields of magnitude 0.0, 0.46, 0.49, and 0.63 V/nm for graphene membranes with nanopore radii of 0.26 (first column) 0.28 (second column), and 0.32 (third column) nm. The first row is the TCFs associated with equilateral triangle configurations with probability of finding $\theta = 60°$, while the bottom row is the TCFs associated with isosceles triangle configurations with probability of finding $\theta = 109°$.

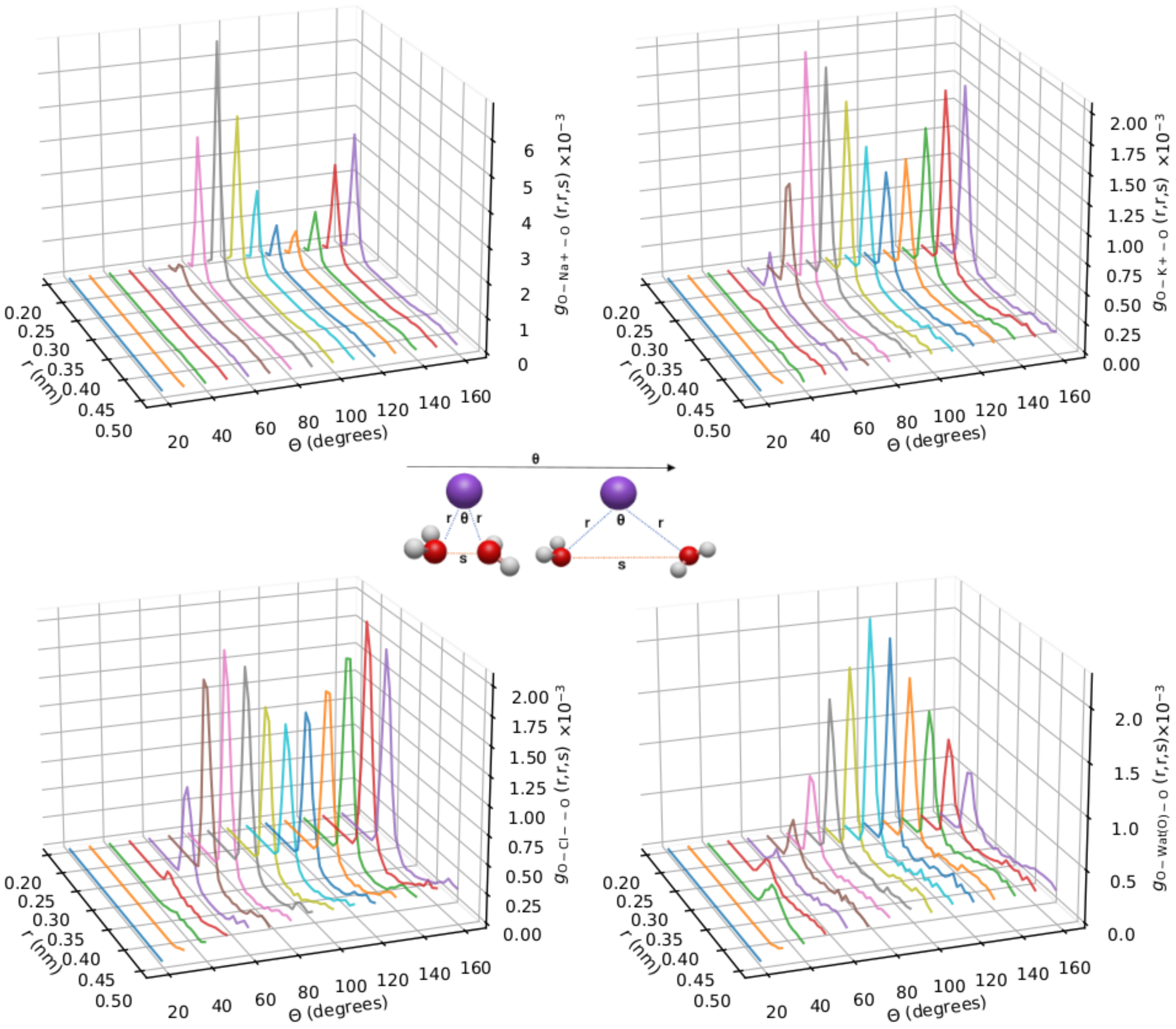

Figure S34: O-A-O TCFs obtained by screening the Θ angle from 20° − 160° with 10° increments for Na^+ (top left), K^+ (top right), Cl^- (bottom left) and oxygen in water (bottom right). A schematic representation of an increase in the magnitude of Θ is shown at the center of the plots.

Results for different force fields

PMF profiles and energy barriers

The maxima observed in the *PMF* profiles, defined as the energy barriers for passage through the nanopore, were determined for different force fields using the 0.26 nm radius nanopore

membrane system for Na^+ and Cl^- ions. The system parameterized with the Loche *et al.* force field for ions and the *SPC/E* model for water molecules was compared to systems using the same methodology, but with Loche *et al.* and *TIP3P* for ions and water, respectively, as well as *Madrid*/2019 for ions and *TIP4P*/2005 for water molecules. The results of this comparison, based on the *PMF* profiles, are shown in Fig.S35.

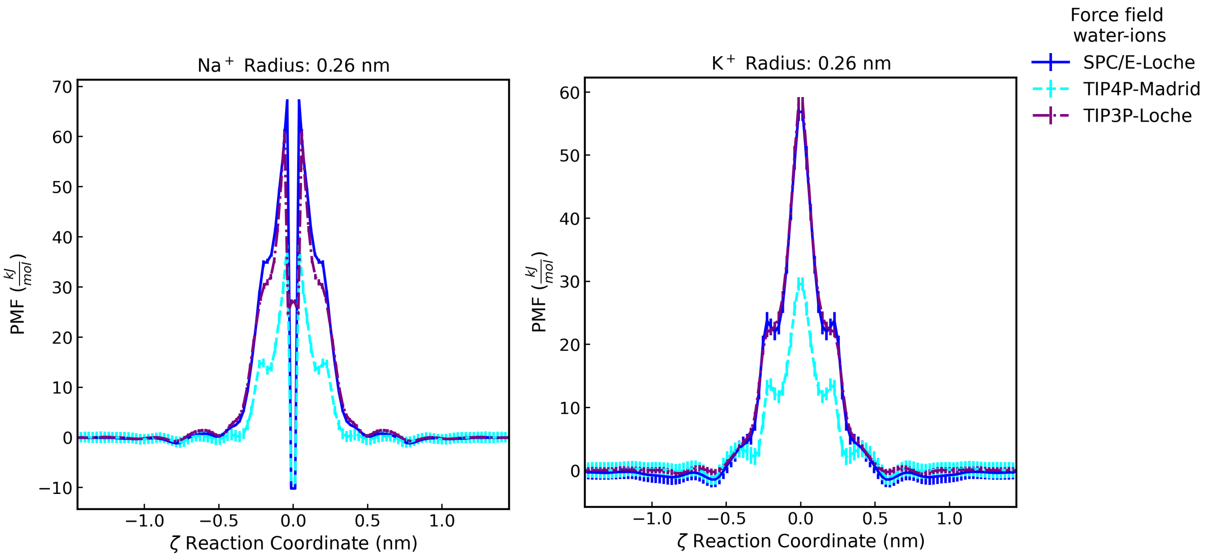

Figure S35: *PMF* profiles computed for Na^+ (left panel) and K^+ (right panel) using different force field parameters for the ion–water systems. The profiles include results using the Loche *et al.* force field for ions with the *SPC/E* model for water molecules (dark blue line), the Loche *et al.* force field for ions with the *TIP3P* model for water molecules (purple line), and the *Madrid*/2019 force field for ions with the *TIP4P*/2005 model for water molecules (cyan line).

The corresponding energy barriers computed from these *PMF* profiles for the same systems using different force field parameters are shown in Fig.S36.

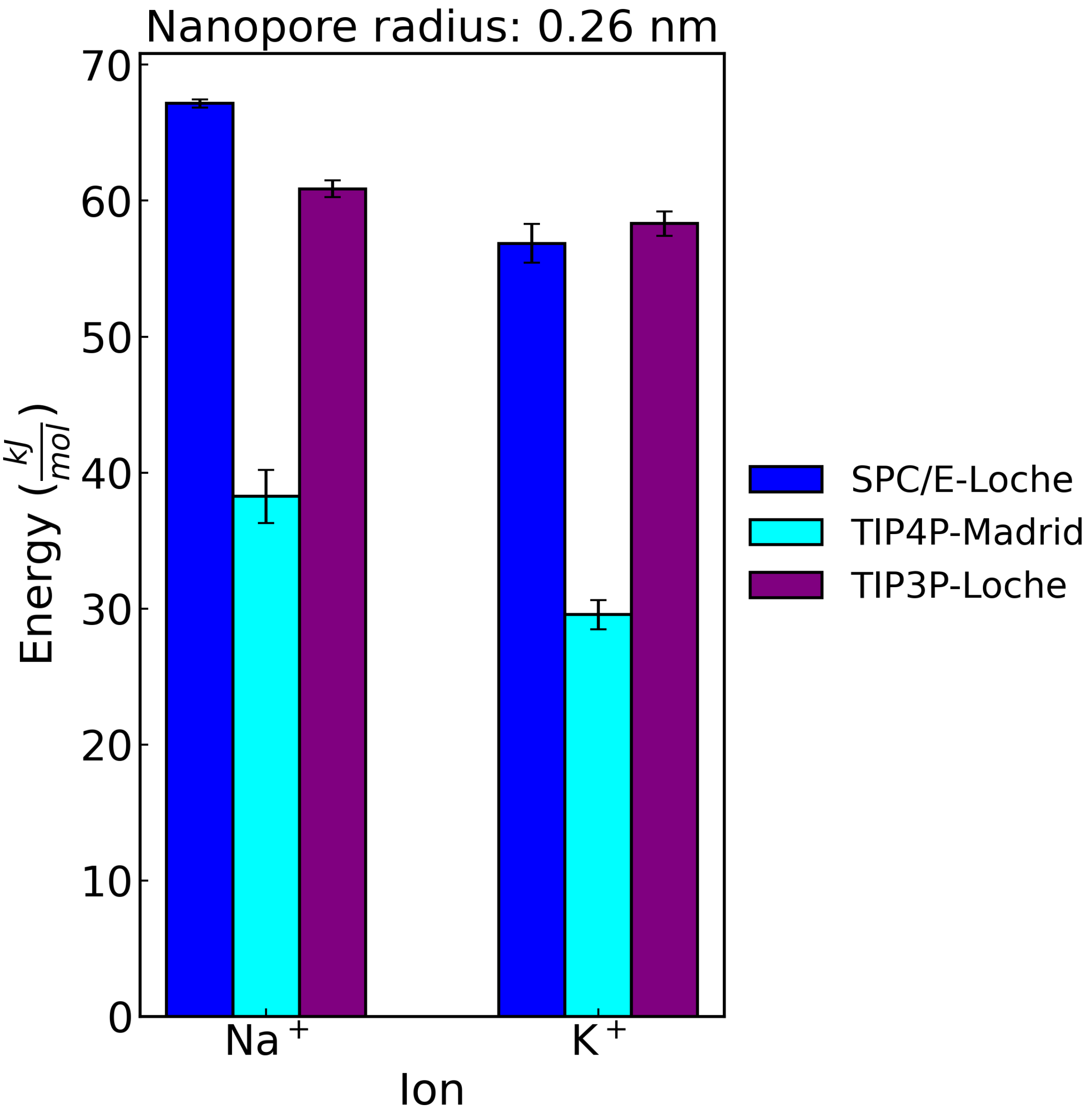

Figure S36: Energy barriers computed for Na^+ and K^+ using different force field parameters for the ion–water systems. The bar plot includes results obtained using the Loche *et al.* force field for ions with the *SPC/E* model for water molecules (dark blue), the Loche *et al.* force field for ions with the *TIP3P* model for water molecules (purple), and the *Madrid*/2019 force field for ions with the *TIP4P*/2005 model for water molecules (cyan).

CPM

The system featuring a nanopore membrane with a 0.26 nm radius was parametrized using the Loche *et al.* force field for ions and the *TIP3P* model for water molecules to enable comparison with results obtained using a different water model. The systems studied in the previous section—employing the *TIP4P*/2005 water model and the *Madrid*/2019 ion force field—were not used in *CPM* simulations due to incompatibilities between the pair style cut-off required by the simulation engine for a 4-point water model and the long-range electrostatic (kspace style = PPPM/conp) method necessary for *CPM* simulations under slab geometry. The system was subjected to three different applied voltages: 3.2, 3.8 and 4.6 V. The results comparing the number of ions sieved as a function of the force field used to parametrize the system are shown in Fig.S37.

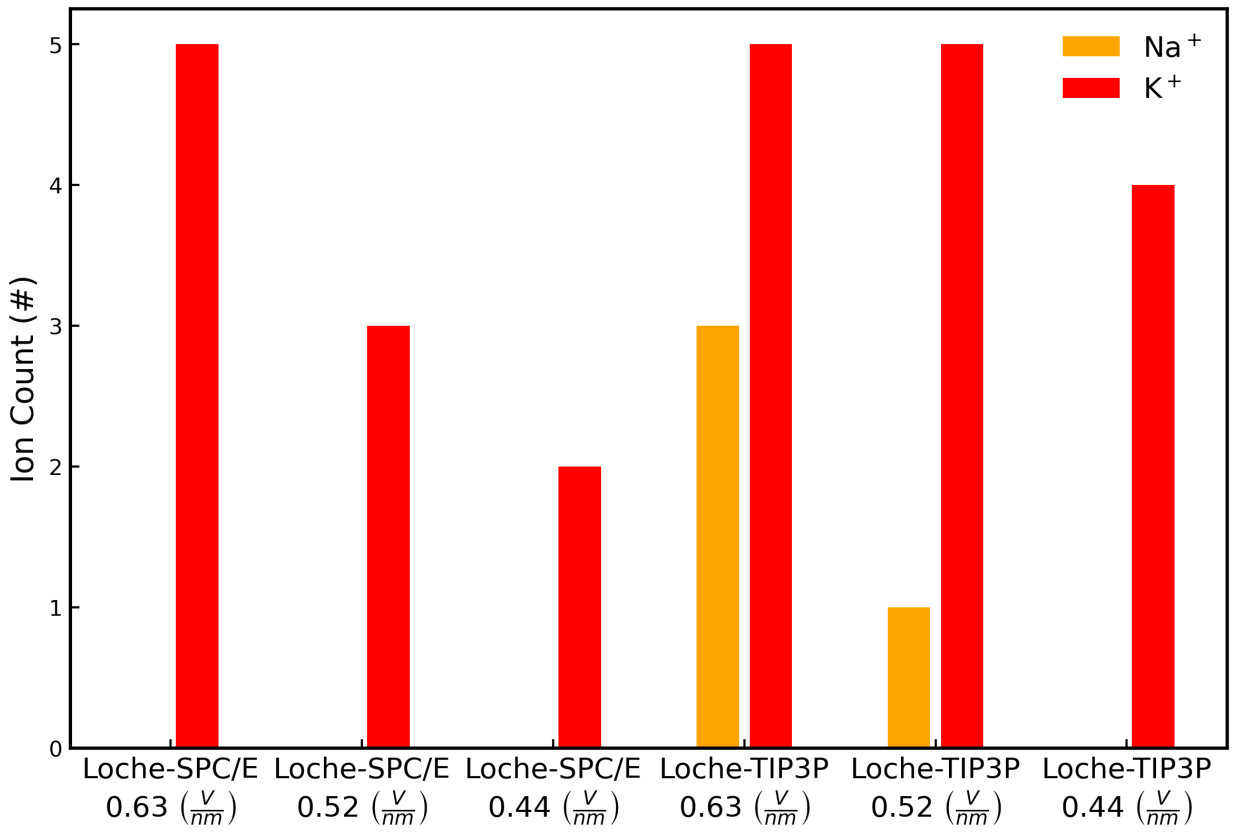

Figure S37: Number of ions that successfully permeated the nanoporous membrane computed for Na^+ (orange) and K^+ (red) using different force field parameters for the ion–water systems. The bar plot includes results obtained using the Loche *et al.* force field for ions with the *SPC/E* model for water molecules and the Loche *et al.* force field for ions with the *TIP3P* model for water molecules.

For the systems modeled with the *TIP3P* water model and the Loche *et al.* force field for ions, the electrostatic potential profile and the electric field profile along the z-direction are shown in Fig.S38. The RDFs and the coordination number analysis of the system are presented in Fig.S39.

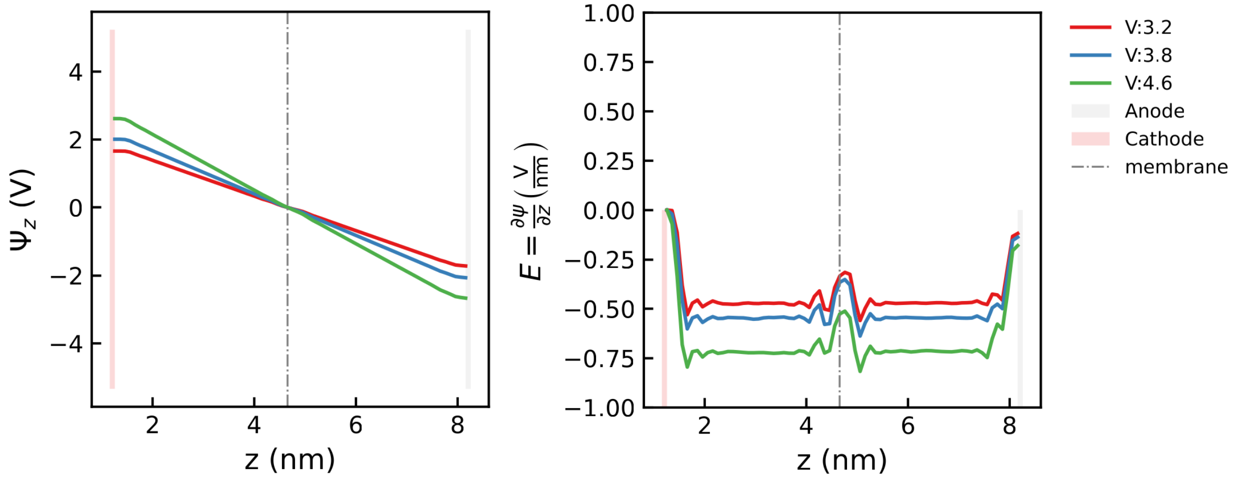

Figure S38: Electric potential profiles (left) and corresponding one-dimensional electric field profiles along the z-direction (right) for applied potential differences of 3.8 and 4.6 ψ across graphene electrodes, for a graphene membrane with a nanopore radius of 0.26 nm. The gray shaded region denotes the anode, the red shaded region the cathode, and the dotted vertical line indicates the position of the graphene membrane.

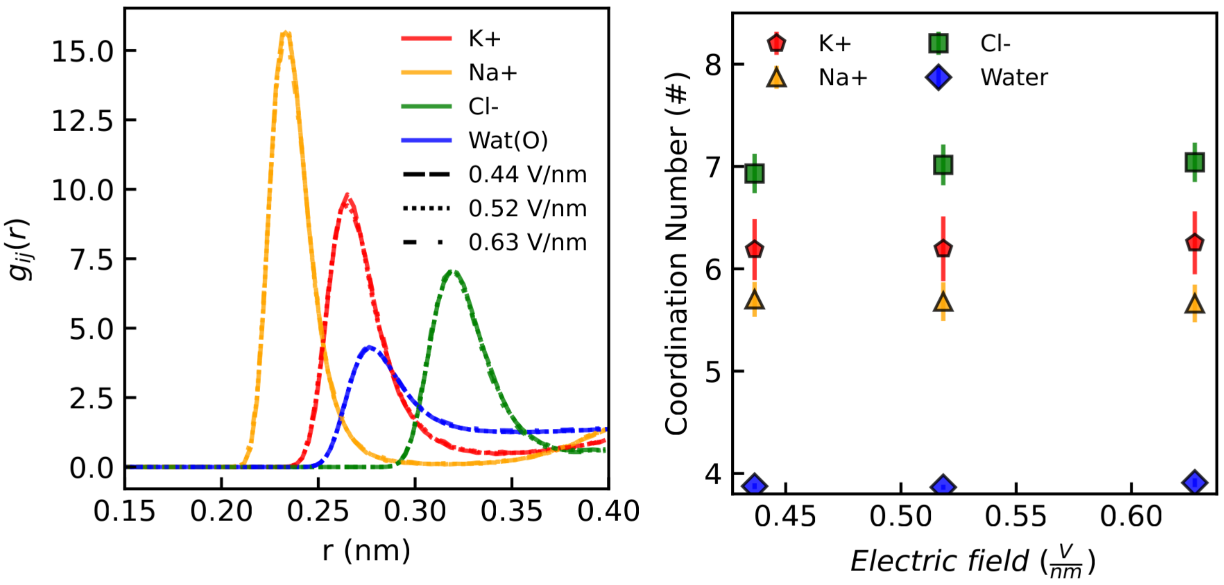

Figure S39: **Left:** *RDF*s $g_{ij}(r)$averaged across a graphene membrane with a radius of 0.26 nm for K^+ (*i.e.*, g_{OK^+}(r), red), Na^+ (*i.e.*, g_{ONa^+}(r), orange), Cl^- (*i.e.*, g_{OCl^-}(r), green), and oxygen in water (*i.e.*, g_{OO}(r), blue) K^+:O (red), Na^+:O(orange), Cl^-:O(green), O:O(blue). **Right:** Coordination numbers within the *FHS* as a function of time, averaged across a graphene membrane with radii of 0.26 nm, and under electric fields of 0.44, 0.52, and 0.63 V/nm. Color coding: K^+:O (red), Na^+:O(orange), Cl^-:O(green), O:O(blue).

The angle between the water dipole and the electric field vector was used to determine

the probability distribution characterizing the orientation of water molecules both near the electrodes and within the bulk of the simulation box. Additionally, the orientation of water molecules within the ion's FHS, and the distance probability distributions between ions or water oxygen atoms and the oxygen atoms of other water molecules within the FHS, are shown in Fig.S40.

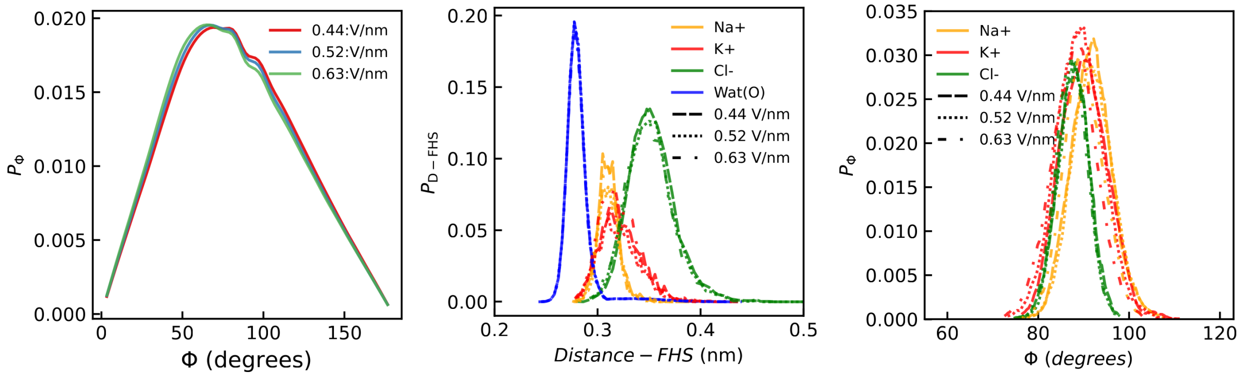

Figure S40: Probability distributions across a graphene membrane with radii of 0.26 nm under external fields of 0.44 V/nm (red), 0.52 V/nm (blue), and 0.63 V/nm (green): **Left:** Distribution of the angle Φ between the water dipole and the electric field vector (0,0,1) in the bulk region, averaged for water molecules. **Middle:** Distance probability between Na^+ (orange), K^+ (red), Cl^- (green), or water oxygens and other water oxygens within the *FHS*. **Right:** Orientation angle distributions between the water dipole and the vector connecting the ion and water oxygen within the *FHS* for Na^+ (orange), K^+ (red), Cl^- (green). A leftward shift in the distributions is observed with increasing field strength.

To evaluate the effects of external electric fields on the dynamic properties of ionic hydration, the mean square displacement (*MSD*) of ions along the field direction (z-axis) was analyzed, as shown in Fig.S41.

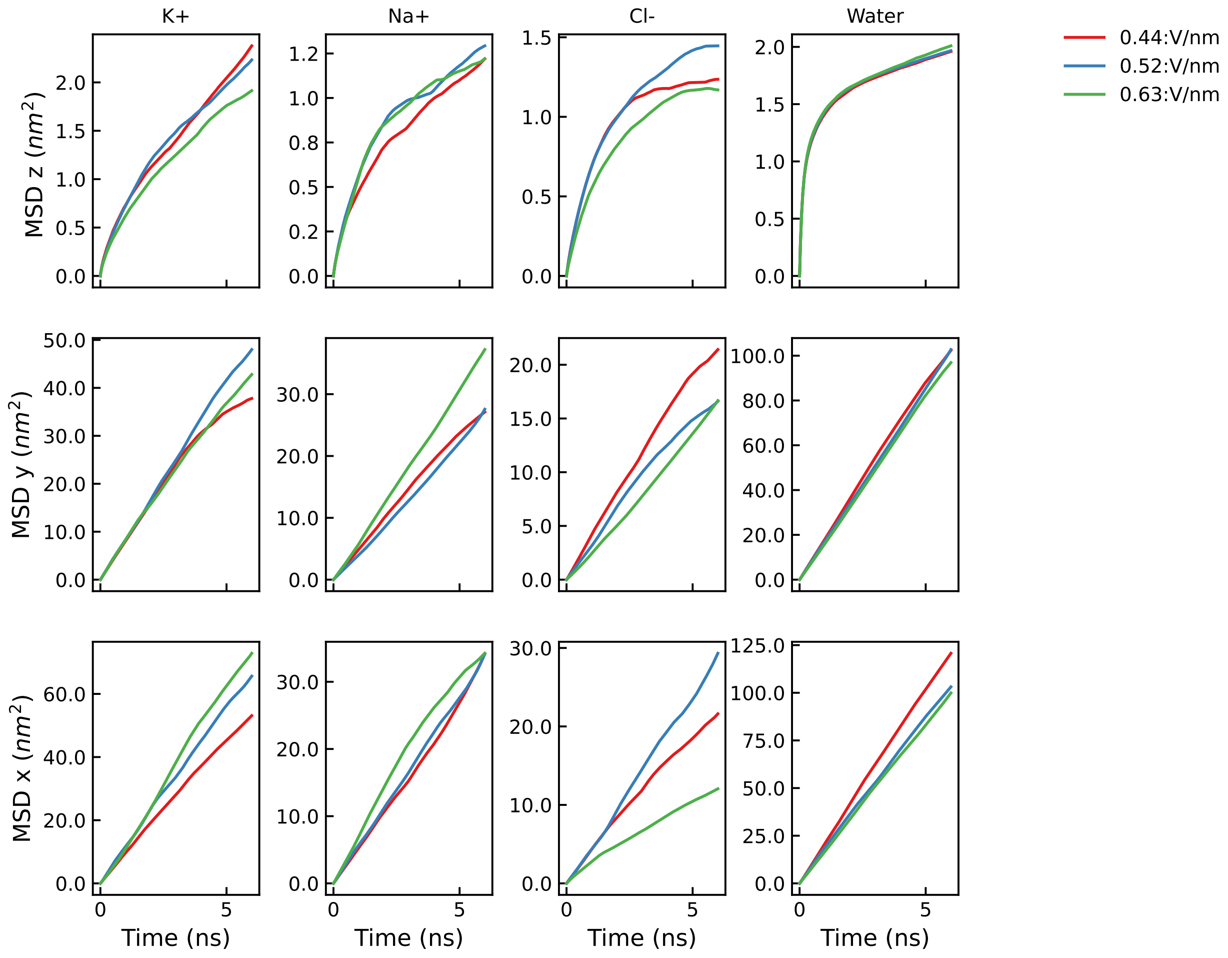

Figure S41: Mean square displacement (MSD) along the field direction (z, top panel), y (middle panel) and x (bottom panel) components averaged across a graphene membrane with a radii of 0.26 nm for K^+ (first column), Na^+ (second column), Cl^- (third column), and Oxygen atom in water (fourth column) molecules under electric fields.

Electrical mobility

The instantaneous drift velocity distributions along the direction of the electric field are compared between systems parametrized using the Loche *et al.* force field for ions, combined with either the *SPC/E* or *TIP3P* water models, as shown in Fig. S42

With the terminal drift velocity across the z-direction, V_z, the electrical mobility of each ion, μ_q, was computed as shown in Fig. ll Using the terminal drift velocity in the z-direction, V_z, the electrical mobility of each ion, μ_q, was computed, as shown in Fig. S43.

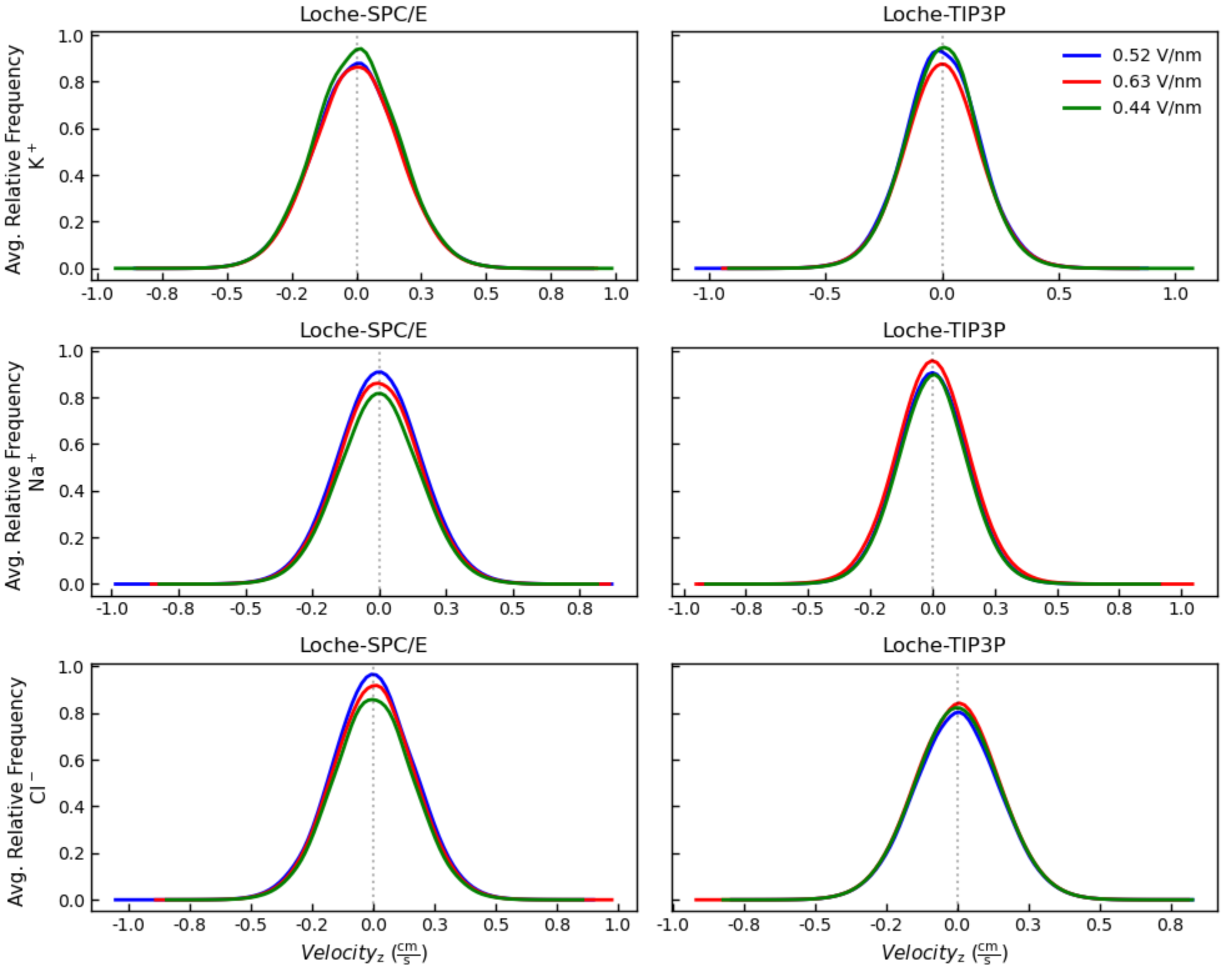

Figure S42: Terminal drift velocity distribution, V_z under external fields generated *via* different potential differences for K^+ (first row), Na^+ (second row), Cl^- (third row) across graphene membranes of nanopore radii 0.26 (first column), 0.28 (second column), and 0.32 (third column) nm. The left panel corresponds to simulations using the Loche *et al.* force field for ions and the *SPC/E* water model, while the right panel uses the Loche *et al.* force field with the *TIP3P* water model.

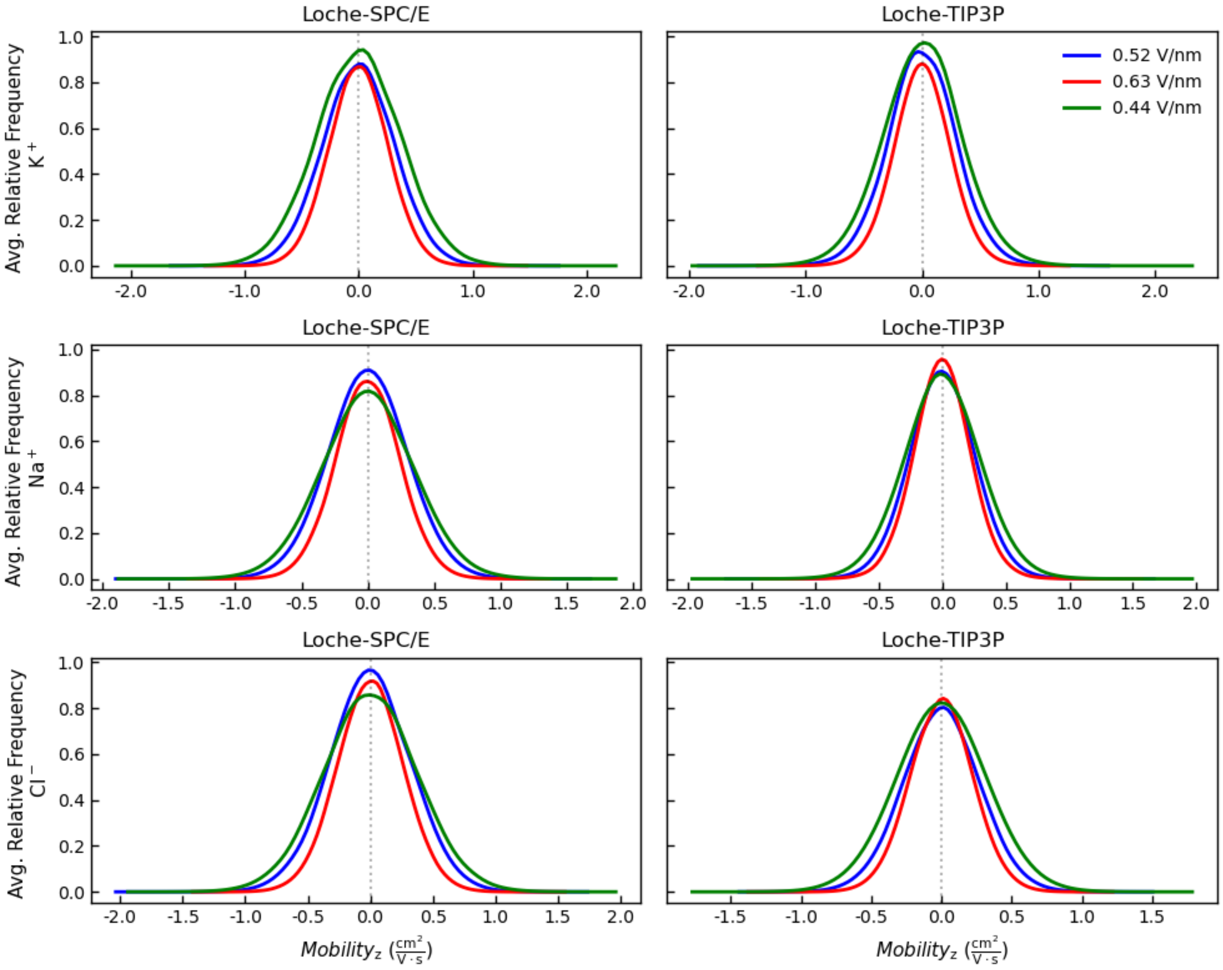

Figure S43: Electrical mobility distribution,μ_q, under external fields generated *via* different potential differences for K^+ (first row), Na^+ (second row), Cl^- (third row) across graphene membranes of nanopore radii 0.26 (first column), 0.28 (second column), and 0.32 (third column) nm.The left panel corresponds to simulations using the Loche *et al.* force field for ions and the *SPC/E* water model, while the right panel uses the Loche *et al.* force field with the *TIP3P* water model.

References

(1) Errington, J. R.; Panagiotopoulos, A. Z. A Fixed Point Charge Model for Water Optimized to the Vapor-Liquid Coexistence Properties. *J. Phys. Chem. B* **1998**, *102*, 7470–7475.

(2) Loche, P.; Steinbrunner, P.; Friedowitz, S.; Netz, R. R.; Bonthuis, D. J. Transferable Ion Force Fields in Water from a Simultaneous Optimization of Ion Solvation and Ion–Ion Interaction. *J. Phys. Chem. B* **2021**, *125*, 8581–8587.

(3) Zeron, I. M.; Abascal, J. L. F.; Vega, C. A Force Field of Li+, Na+, K+, Mg2+, Ca2+, Cl-, and So42- in Aqueous Solution Based on the Tip4p/2005 Water Model and Scaled Charges for the Ions. *J. Chem. Phys.* **2019**, *151*, 134504.

(4) Cole, M. W.; Klein, J. R. The Interaction Between Noble Gases and the Basal Plane Surface of Graphite. *Surf. Sci.* **1983**, *124*, 547–554.

(5) Abascal, J. L. F.; Vega, C. A General Purpose Model for the Condensed Phases of Water: Tip4p/2005. *J. Chem. Phys.* **2005**, *123*, 234505.

(6) Roux, B. The Calculation of the Potential of Mean Force Using Computer Simulations. *Comput. Phys. Commun.* **1995**, *91*, 275–282.

(7) Hub, J. S.; de Groot, B. L.; van der Spoel, D. G_wham– a Free Weighted Histogram Analysis Implementation Including Robust Error and Autocorrelation Estimates. *J. Chem. Theory Comput.* **2010**, *6*, 3713–3720.

(8) Lin, X.; Tee, S. R.; Searles, D. J.; Cummings, P. T. Molecular Insights on Optimizing Nanoporous Carbon-based Supercapacitors With Various Electrolytes. *Electrochim. Acta* **2024**, *474*, 143500.

(9) Bonakala, S.; Hasan, M. I. Comparative Study of External Electric Field and Potential Effects on Liquid Water Ions. *Mol. Phys.* **2022**, *120*, e1998689.

(10) Lai, R.; Dodds, E. D.; Li, H. Molecular Dynamics Simulation of Ion Mobility in Gases. *J. Chem. Phys.* **2018**, *148*, 064109.

(11) Lee, S. H.; Rasaiah, J. C. Molecular Dynamics Simulation of Ionic Mobility. I. Alkali Metal Cations in Water at 25°C. *J. Chem. Phys.* **1994**, *101*, 6964–6974.
